# A genome-wide survey of DNA methylation in hexaploid wheat

**DOI:** 10.1186/s13059-015-0838-3

**Published:** 2015-12-10

**Authors:** Laura-Jayne Gardiner, Mark Quinton-Tulloch, Lisa Olohan, Jonathan Price, Neil Hall, Anthony Hall

**Affiliations:** Institute of Integrative Biology, University of Liverpool, Crown Street, Liverpool, UK

**Keywords:** DNA methylation, *Triticum aestivum*, Polyploidy

## Abstract

**Background:**

DNA methylation is an important mechanism of epigenetic gene expression control that can be passed between generations. Here, we use sodium bisulfite treatment and targeted gene enrichment to study genome-wide methylation across the three sub-genomes of allohexaploid wheat.

**Results:**

While the majority of methylation is conserved across all three genomes we demonstrate that differential methylation exists between the sub-genomes in approximately equal proportions. We correlate sub-genome-specific promoter methylation with decreased expression levels and show that altered growing temperature has a small effect on methylation state, identifying a small but functionally relevant set of methylated genes. Finally, we demonstrate long-term methylation maintenance using a comparison between the D sub-genome of hexaploid wheat and its progenitor *Aegilops tauschii*.

**Conclusions:**

We show that tri-genome methylation is highly conserved with the diploid wheat progenitor while sub-genome-specific methylation shows more variation.

**Electronic supplementary material:**

The online version of this article (doi:10.1186/s13059-015-0838-3) contains supplementary material, which is available to authorized users.

## Background

The wheat genome is allohexaploid, 17 Gb in size and is derived from three diploid progenitor genomes (AABBDD). The A sub-genome originates from *Triticum urartu*, the B sub-genome an unknown species related to *Aegilops speltoides*, and the D sub-genome from *Aegilops tauschii*. AABB tetraploids (*Triticum turgidum*) appeared less than 0.5 million years ago, and bread wheat from a further hybridization with the D genome 10,000 years ago [1]. Its size, polyploidy and high repeat content (~80 %) have made sequencing and analysis challenging [2]. Recent sequencing efforts have utilized targeted capture re-sequencing to analyze the genic portion of hexaploid wheat [3, 4].

Cytosine methylation of DNA acts as a mechanism of gene expression control. In plants, it occurs typically at CpG residues but can also occur at CHG and CHH sites (where H represents adenine, cytosine or thymine) [5]. In *Arabidopsis* most methylation in the gene body occurs at CpG sites with non-CpG methylation seen at lower levels, whilst methylation elsewhere and in repetitive regions occurs at CpG, CHH and CHG sites [6, 7]. Methylation within gene promoter regions is thought to inhibit regulatory protein binding and repress transcription (and can also silence transposable elements), whereas methylation within introns and exons is correlated with highly expressed genes [6].

The potential for differential methylation of homoeologous genes in a polyploidy species such as wheat is an important question, where it could control gene dosage between the three sub-genomes. Differential or allele-specific methylation has been frequently observed in humans [8] and plants [9] and has been correlated with changes in gene expression. It is thought that differential methylation of a region rather than a single position is more likely to contribute to gene expression change. However, no detailed study exists comparing homoeologous genes in a complex polyploid.

Here, we develop an enrichment system to investigate methylation patterns across ~81 Mb of the genic regions of the allohexaploid wheat genome. This allows us for the first time to explore epigenetic variation in this large complex genome and open up a new level of genetic variation, which can be exploited by breeders. We implemented this genome-wide methyl-Seq approach to test a number of hypotheses in wheat: firstly, that differential methylation exists between the sub-genomes; secondly, that temperature is capable of altering the methylation state in both a sub-genome-specific and sub-genome-independent manner; thirdly, that it is this underlying methylation that correlates with both sub-genome-specific and temperature-dependent changes in gene expression; and finally, that methylation is maintained over long time periods but that differences in methylation accumulate over time.

## Results and discussion

### Development of a platform for genome-wide analysis of methylation patterns in wheat

To investigate genome-wide methylation patterns in the 17-Gb allohexaploid wheat genome, we used genomic enrichment followed by bisulfite treatment [10] (Agilent SureSelect Methyl-Seq) and Illumina HiSeq paired-end sequence generation. Based on DNA sequence, 50,000 120-mer RNA baits were each designed to capture a subset of wheat genes totaling 18 Mb; 6 Mb from each of the three sub-genomes of wheat (Figure S1 in Additional file 1). The use of paired-end Illumina libraries, which extend beyond the baits, necessitated a reference sequence that was constructed using the baits plus surrounding contiguous DNA sequence, based on assemblies from Brenchley et al. [2]. These extended reference contigs ranged from 121–8835 bp with a median length of 698 bp. The total size of the potential mapping reference was ~44 Mb per sub-genome.

To test the performance of our experimental pipeline; we investigated reproducibility between biological replicates, conversion rates of the bisulfite treatment and enrichment bias between the three sub-genomes. Total genomic DNA was extracted from 7-day-old Chinese Spring wheat seedlings; three were grown at 12 °C and three at 27 °C. Genomic DNA was enriched, bisulfite treated, sequenced and mapped to the reference sequences using Bismark [11]. Cytosine residues could then be classified as methylated or un-methylated.

The samples had an average sequencing depth of 102× across 96.3 % of the 6-Mb bait sequence (Table S1 in Additional file 1). The depth of coverage across the probe set was consistent (Figure S2 in Additional file 1) with less than 0.2 % of baits exceeding tenfold the average depth of coverage. Mapping statistics between the 12 °C and 27 °C replicates were therefore comparable and 99.7 % of single-nucleotide polymorphisms (SNPs) were conserved between sample replicates at positions that were mapped to a minimum depth of 15× per replicate. Furthermore, high conservation of methylation was seen between replicate samples using the software Methylkit [12] with <0.09 % of residues showing differential methylation (Note 1 and Figures S3 and S4 in Additional file 1); moreover, any sites that were considered differentially methylated between replicates were excluded from sample-wise comparisons.

An independent analysis was carried out on a subset of methylation and SNP calls for validation. Here, Sanger sequencing was utilized and ~83 % of SNPs analyzed from both samples were validated and ~90 % of methylation sites were confirmed in both samples (see "Materials and methods"; Table S2 and Figure S5 in Additional file 1).

Identifying a high concordance between replicate samples allows us to pool replicates for downstream analyses, thus increasing coverage to an average depth of 297.6× with 97.5 % of the 6-Mb capture probe sequences being mapped to (Table 1). Moreover, an average depth of coverage of 128× was observed with 27 Mb of the extended 44 Mb reference being mapped to by both replicate pools. Using homoeologous SNPs to distinguish the three sub-genomes, this 27 Mb translates to approximately 81 Mb of the allohexaploid genome that could be analyzed. In the 12 °C sample Bismark [11] identified 7,813,105 cytosine residues per sub-genome (methylated and un-methylated) and 8,069,906 in the 27 °C sample that could be interrogated to determine their methylation state; numbers vary between datasets due to slight differences in the coverage of the reference. Mapping reads to the non-methylated chloroplast genome was used to assess bisulfite conversion efficiency; using strict mapping parameters, 98.92 % of cytosine bases were successfully bisulfite converted. This is within the limits determined for successful bisulfite conversion in previous studies [13].

**Table 1:** Mapping statistics

Sample	Average percentage coverage per 120-mer probe	Average depth of coverage per 120-mer probe	Number of 120-mer probes mapped	Percentage of 120-mer probes mapped
12 °C	98.4	290.8	49,986	99
27 °C	98.5	304.4	49,982	99

The mapping output statistics for the two enriched and bisulfite-treated wheat DNA samples in relation to the 6-Mb capture probe base space

In our extended reference sequence 72,345 homoeologous SNP positions were identified for sub-genome discrimination using a combination of two methodologies: firstly, we mapped International Wheat Genome Sequencing Consortium (IWGSC) homoeologous SNPs to our 44-Mb reference (see "Materials and methods"). This left some regions of extended reference with no homoeologous SNP calls; therefore, we mapped polymorphic bases from the genomes of diploid wheat ancestors to our reference to generate additional homoeologous SNPs (see "Materials and methods"). Comparing overlapping SNPs between the two methods validated this second method; 80 % of SNP alleles were conserved, giving high confidence in these inferred SNPs.

The homoeologous SNP information was used to assign 7,813,105 and 8,069,906 cytosines in the 12 °C and 27 °C samples, respectively, to specific sub-genomes (outlined in Figure S6 in Additional file 1). This enabled us to identify 318,452 residues for the 12 °C sample and 324,227 for the 27 °C sample either where all three sub-genomes could each be identified at a depth of 5× or more (28,632 and 29,202 residues for the 12 °C and 27 °C samples) or where biallelic SNPs allowed categorization of reads at a position as A or BD, B or AD and D or AB genome-specific with a requirement of 5× coverage or more for a single genome and 10× or more for a genome pair (289,820 and 295,025 residues for the 12 °C and 27 °C samples). These residues mapped across the 27 Mb of the reference sequence and were annotated for exon, intron and predicted promoter regions using the MIPS gene models (v2.2): 8902 genes were represented partially or fully (per sub-genome), including 244 promoters, 6356 genes with one or more exon and 4450 genes with one or more intron; 3702 genes had both exons and introns represented.

Uniform enrichment was seen across the wheat sub-genomes with approximately one-third of sequencing reads mapping per genome. There was also no significant difference in efficacy of enrichment of methylated compared with non-methylated DNA sequence. Finally, there was negligible difference between the capture efficiency in exons/introns and non-transcribed regions. However, fewer homoeologous SNPs could be confidently identified in repetitive non-transcribed regions for sub-genome discrimination (Note 2 in Additional file 1).

### Global methylation patterns match other plant species

Methylation in plants is found in CpG, CHG and CHH contexts. In rice the percentages of methylated cytosines found at CpG, CHG and CHH sites were 54.7 %, 37.3 % and 12.0 %, respectively, while the average methylation levels (i.e., the proportion of reads showing a methylated cytosine among all reads covering the same cytosine site) in the three contexts were 44.5 %, 24.1 % and 4.7 % [14]. Here, looking at the 318,452 residues for the 12 °C sample, the percentages of methylated cytosines were 60.0 %, 4.58 % and 1.42 % across CpG, CHG and CHH sites, respectively. These figures were conserved across the wheat sub-genomes and in the 27 °C sample. The average methylation levels in wheat in the three sequence contexts were 53.3 %, 3.48 % and 1.41 % across the sub-genomes and the 12 °C and 27 °C samples. Similarly to rice and *Arabidopsis*, we see a tendency towards high-level methylation in the CpG context but low-level methylation in the CHH context. Notably, we also observe very low levels of both CHG and CHH methylation (Figure S7 in Additional file 1). Since our analysis mainly focuses on gene body methylation, with 80 % of the analyzed cytosine residues found in intron/exon sequences, this is an anticipated result, since in *Arabidopsis* and rice there is enrichment for CpG methylation in gene bodies [6, 7]. In predicted non-transcribed regions we see an increase in CHG methylation; average methylation levels in wheat in the three sequence contexts were 50.7 %, 10.4 % and 1.77 % across the sub-genomes and the two samples, which is more comparable to the patterns in rice and *Arabidopsis*.

To discriminate methylated CpG, CHG and CHH sites from non-methylated residues, we defined standard thresholds for each category based on published methodologies (see "Materials and methods"). These thresholds take into account the tendency for high-level CpG methylation and low-level non-CpG methylation. Using these thresholds over the cytosine residues where all three sub-genomes could be distinguished at a depth of 5× or more (318,452 residues for the 12 °C sample and 324,227 for the 27 °C), 11.9 % of the sites that were analyzed were methylated in one or more sub-genomes; 12.5 % of residues under analysis were CpG sites, with 62.0 % methylated in one or more sub-genomes; 20.5 % of residues were CHG sites, with 9.5 % methylated; and 67 % of residues were CHH sites with 3.4 % methylated.

Analyzing methylation distribution between CpG, CHH and CHG sites using these thresholds, we found that 71.4 % of methylated sites in transcribed regions were CpG sites, 18.1 % were CHH sites and 10.5 % were CHG sites; in un-transcribed regions, 46.3 % of methylated sites were CpG sites, 21.7 % were CHH sites and 32.0 % were CHG sites. In previous studies in plants such as *Arabidopsis* [6, 15] mostly methylated CpG sites were found in coding regions while CpG, CHG and CHH methylated sites were seen in non-coding regions. Here, unlike *Arabidopsis*, all types of methylation are a significant presence in both transcribed and non-transcribed regions, although an increase in CHH/CHG site methylation was observed in non-transcribed regions.

### Transposons are highly methylated

Off-target sequencing data were found to be unbiased carryover DNA, equivalent to low coverage shotgun sequencing of total wheat DNA, since they comprised mainly repetitive sequence and proportions of transposon types closely matched those seen in previous shotgun sequence data [2] (Table S3 in Additional file 1). It was noted that transposons in general were highly methylated in comparison with the enriched gene-rich regions. Hyper-methylation of repeats is consistent with other plant species and is associated with reducing transposon mobilization [5] (Note 3 in Additional file 1).

### Conserved methylation across all three genomes predominates but genome-specific methylation is significant

**Fig. 1:**
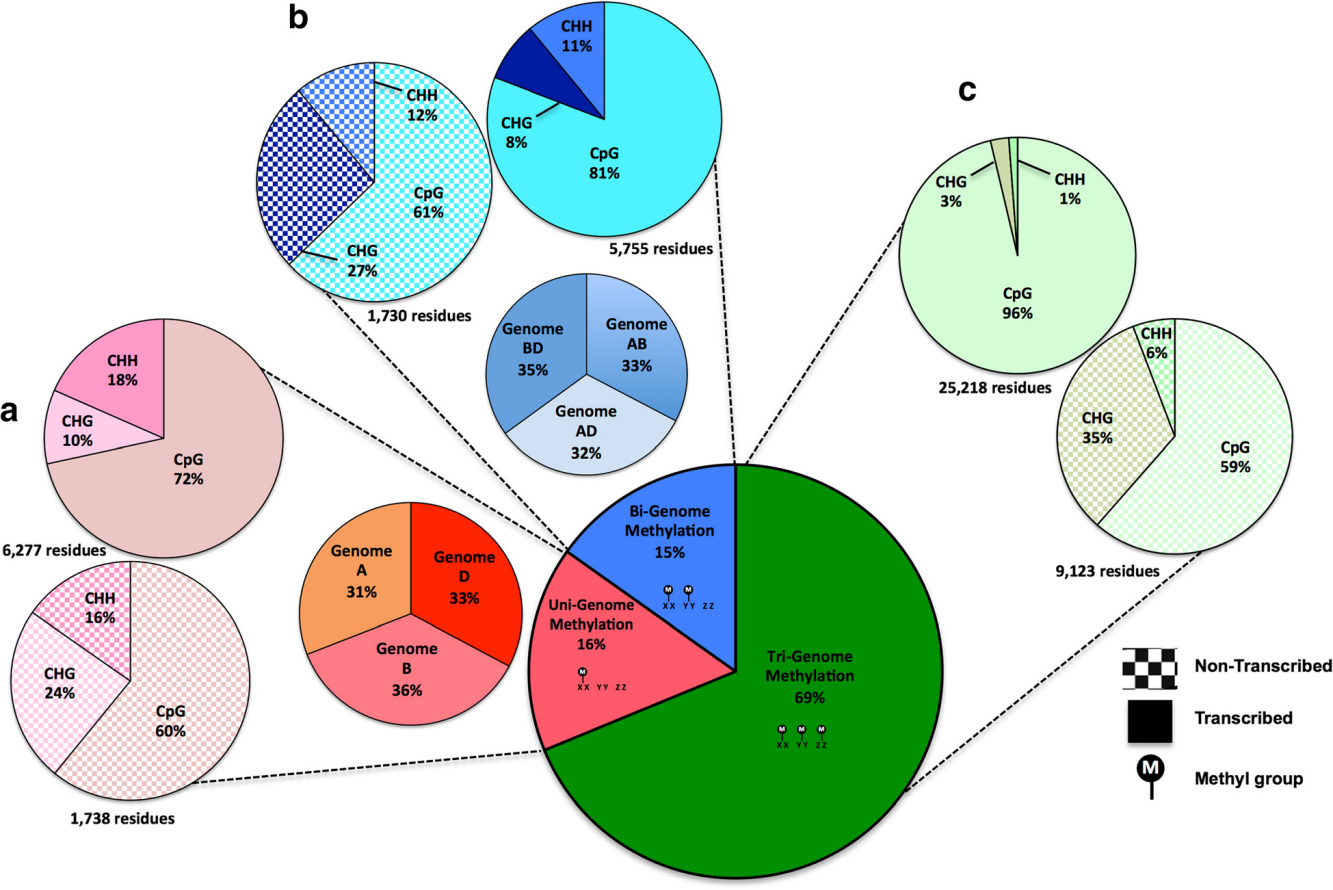
Categorizing observed methylation averaged across the 12 °C and 27 °C sample datasets. **a** Uni-genome methylation distribution between the three sub-genomes and an overview of its occurrence at CpG, CHG and CHH sites in transcribed and non-transcribed/promoter regions (averaged over all three sub-genomes due to high similarity). **b** Bi-genome methylation distribution between the three pairs of sub-genomes and an overview of its occurrence at CpG, CHG and CHH sites in transcribed and non-transcribed/promoter regions (averaged over all three pairings due to high similarity). **c** Tri-genome methylation and its occurrence at CpG, CHG and CHH sites in transcribed and non-transcribed/promoter regions

Methylation can be further classified as uni-genome (methylation of a single sub-genome at a site where the other two are non-methylated), bi-genome (methylation of two sub-genomes at a site where the other sub-genome is non-methylated) or tri-genome (methylation of all three sub-genomes). Tri-genome methylation was recorded using our standard thresholds. Differential (uni- and bi-genome) methylation between the A, B and D sub-genomes was recorded using the tool Methylkit [12] to identify a minimum difference of 50 % and q < 0.01 combined with our standard thresholds for identification of methylated regions (see "Materials and methods"). Using these thresholds, differential methylation between the sub-genomes was observed across the 12 °C and 27 °C samples at 2.4 % of all analyzed cytosine residues and 20 % of methylated cytosine residues; 45 % of methylated residues showed tri-genome methylation and the remaining 35 % showed intermediate level methylation (see "Materials and methods"). Table S4 in Additional file 1 details differentially and tri-genome methylated CpG, CHH or CHG sites and their transcription statuses for the 12 °C and 27 °C samples. Figure 1 presents these data averaged between the samples. The A, B and D sub-genomes show uni-genome methylation in similar proportions and methylation levels in the bi-genome methylation group were also conserved across the genome pairs and the two samples (Note 4 in Additional file 1).

The proportions of methylated CpG, CHH and CHG sites are conserved across the two samples, the three sub-genomes, and within transcribed and non-transcribed regions, although the ratios differ. In transcribed or gene body regions, uni-genome methylation is mostly at CpG sites (72 %), although CHH and CHG sites are still present at 18 % and 10 %, respectively. Bi-genome methylation follows a similar pattern with 81 % of methylation at CpG sites and 11 % and 8 % at CHH and CHG sites, respectively. In non-transcribed regions, the percentage of uni-genome methylation at CpG sites drops to 60 % and an increase in the number of methylated CHG sites is seen (24 %), with 16 % of sites in the CHH context. Bi-genome methylation again follows a similar pattern, with 61 % of methylation at CpG sites and 12 % and 27 % at CHH and CHG sites, respectively. In summary, across all sub-genomes for uni- and bi-genome methylation we see a drop in CpG and an increase in CHH/CHG methylation in non-transcribed regions, consistent with other plant genomes.

Across the 12 °C and 27 °C datasets 45 % of methylated residues showed tri-genome methylation. In the 12 °C and 27 °C samples, in transcribed regions, the tri-genome methylated residues are almost exclusively CpG sites (96 %); this differs from the observation for uni- and bi-genome associations where CHG and CHH methylation is still significant. In contrast, in non-transcribed regions, similar to differentially methylated sites, we observe a decrease in specificity for CpG sites (59 %) and an enrichment for CHG and CHH methylation (35 % and 6 %). It is possible that the presence of CHG/CHH uni- and bi-genome methylation in transcribed regions could associate with pseudo genes in wheat.

Gene Ontology (GO) analysis using GOEAST [16] highlighted common functional terms associated with the methylated sites that were specific to each of the A, B and D sub-genomes in the 12 °C sample (*p* <0.05; Table S5 in Additional file 1). The A sub-genome’s enriched genes relate to biosynthetic/metabolic processes and response to external stimulus. The B sub-genome showed enrichment for terms such as biosynthetic/metabolic processes, growth and membrane/cell wall association. It also showed enrichment for terms associated with histone binding and translation initiation. Finally, the D sub-genome’s enrichment profile contained terms associated with zinc ion trans-membrane transport and cellular homeostasis. The enriched GO terms for the 27 °C sample were conserved with the 12 °C sample, although in the 27 °C sample for sub-genome A we identified additional terms relating to stress response.

**Fig. 2:**
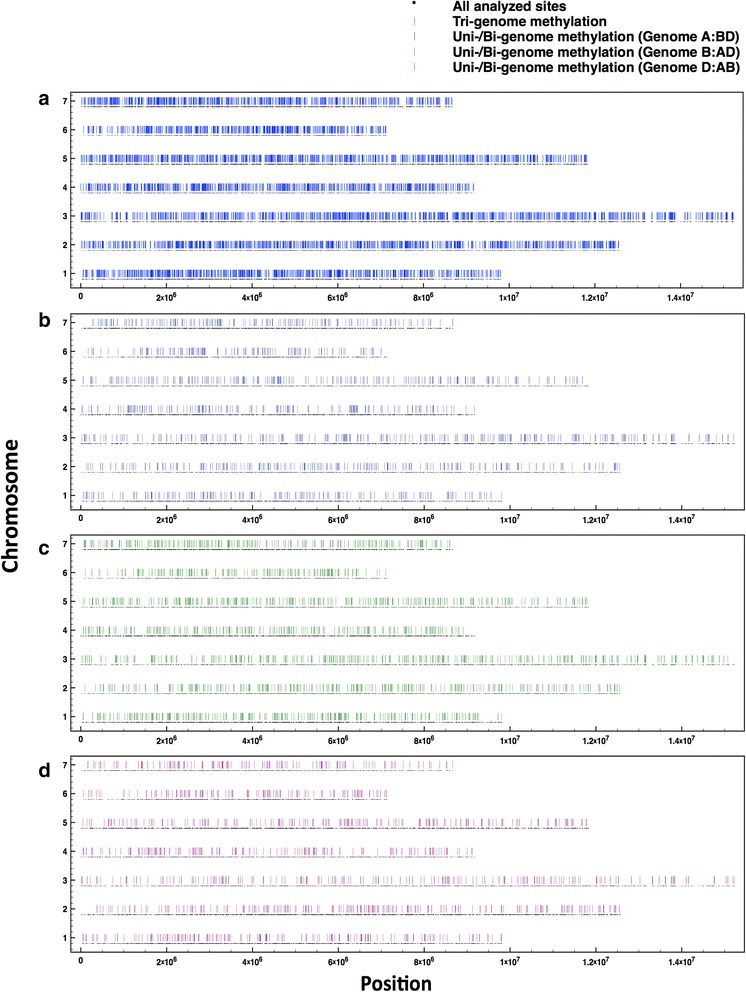
Positional information for methylation sites. Incidences of tri-genome methylation (**a**) and uni-/bi-genome methylation in genome A/BD (**b**), genome B/AD (**c**) and genome D/AB (**d**) in the 12 °C sample are detailed relative to all analyzed sites (methylated and non-methylated). Data are shown along each of the pseudo-chromosomes and methylation is classified using threshold values plus a 50 % difference and q < 0.01 for sites showing differential methylation between the sub-genomes

Plotting uni-, bi- and tri-genome methylation for the 12 °C sample onto pseudo-wheat chromosomes based on synteny with POPSEQ-based pseudomolecules [17, 18], an even distribution of methylation is observed across the genomes genic sequence, with no obvious hotspots or bias towards chromosome ends. Most gaps are due to missing information rather than a break in methylation (Fig. 2; Figure S8 in Additional file 1 for the 27 °C sample). Furthermore, uni-, bi- and tri-genome methylation does not appear to target transcribed/non-transcribed regions specifically; 73 % of tri-genome methylated sites are in transcribed regions, compared with 78 % showing uni-genome methylation and 77 % bi-genome methylation (Table S4 in Additional file 1). None of these three proportions deviate by more than 10 % from the 80 % of residues that were found to be in transcribed regions in the full dataset.

### Identification of differentially methylated regions

Gene expression changes are often associated with methylated regions rather than single methylated nucleotides. To survey differentially methylated regions (DMRs) across the genome it is preferable to analyze tiled windows across it, typically of 1000 bp [19]. As this analysis is based on fragmented gene regions rather than whole genome sequence, we summarized methylation per extended bait probe and compared these figures between the sub-genomes of wheat to identify DMRs. We used Methylkit for this analysis and focused only on regions that were identified between sub-genomes with a q value below 0.01 using the Fisher's exact test. The three sub-genomes were compared and a region selected if a single genome was at least 25 % more methylated than the other two. In the 12 °C sample 11 DMRs were found to be specific to genome A, 15 specific to genome B and 4 specific to genome D; these figures were 7, 18 and 5, respectively, for the 27 °C sample. Similar to the enriched GO terms for uni-genome methylation, sub-genome A’s densely methylated gene regions relate to metabolic processes, sub-genome B’s relate to metabolic processes, binding and membrane association and sub-genome D’s relate to zinc ion trans-membrane transport. Sixteen DMRs are conserved between the two temperatures. All DMRs are detailed in Table S6 in Additional file 1.

### Promoter methylation correlates with a decrease in gene expression

Using the same leaf material used for the methyl-Seq analysis, RNA-seq data were generated and analyzed using Bowtie2 [20] for mapping and BitSeq [21] for allelic expression level estimates to enable direct comparison with methylation patterns. Allelic expression levels are given as a percentage of the maximum expression that was observed. By aligning our extended baits to the wheat genome we estimate that they represent 22,696 (partial or full) wheat genes per sub-genome. For 87.8 % of wheat genes one or more of the three sub-genomes showed measurable expression in the 12 °C sample; this is similar to the 88 % found when Pfeifer et al. examined the expression of homoeologous gene triplets [22]. We may see a bias for expressed genes after enrichment due to the use of cDNA sequence for probe design. Analyzing the co-expression levels for expressed gene loci, 31.3 % showed closely conserved expression levels between the three sub-genomes (within 5 %); 46.2 % showed uni-genome expression increased by a minimum of 5 % above the other two sub-genomes; 14.7 % showed bi-genome expression increased by a minimum of 5 % above that of the other sub-genome. This leaves 7.8 % where expression levels differ between all three sub-genomes. Although our analysis and parameters differ, we can still see a similar pattern to that observed by Pfeifer et al. [22] where approximately one-third of homoeologous triplet genes show co-expression of all three genes, and co-expressed gene pairs are the most frequently encountered expression category and are relatively uniformly distributed across the genome pairs (28.3 %, 39.8 % and 31.9 % for sub-genomes BD, AD and AB, respectively).

To investigate correlation between CpG, CHH and CHG regional methylation and gene expression in the 12 °C sample, the percentage methylation per extended bait probe and per sub-genome was summarized and compared with the allelic expression for the same region (Figure S9 in Additional file 1). Here, methylation was categorized as promoter, non-transcribed or transcribed intron/exon and promoters/non-transcribed regions were assigned the expression of their neighboring gene. Methylation levels were largely conserved between transcribed and promoter regions with no significant differences found; however, average allelic expression levels were significantly lower when associated with promoter region methylation compared with transcribed region methylation in the associated gene (two-tailed *t* test, *p* < 0.05; Table S7 in Additional file 1). Correlation between single point CpG, CHH and CHG methylation level and allelic expression was also summarized in the same way for the 12 °C sample. Comparisons of this methylation across transcribed and promoter regions were made (Figure S10 in Additional file 1) but no correlation was observed at the single point level.

### Methylation of a single genome affects genome-specific gene expression

**Fig. 3:**
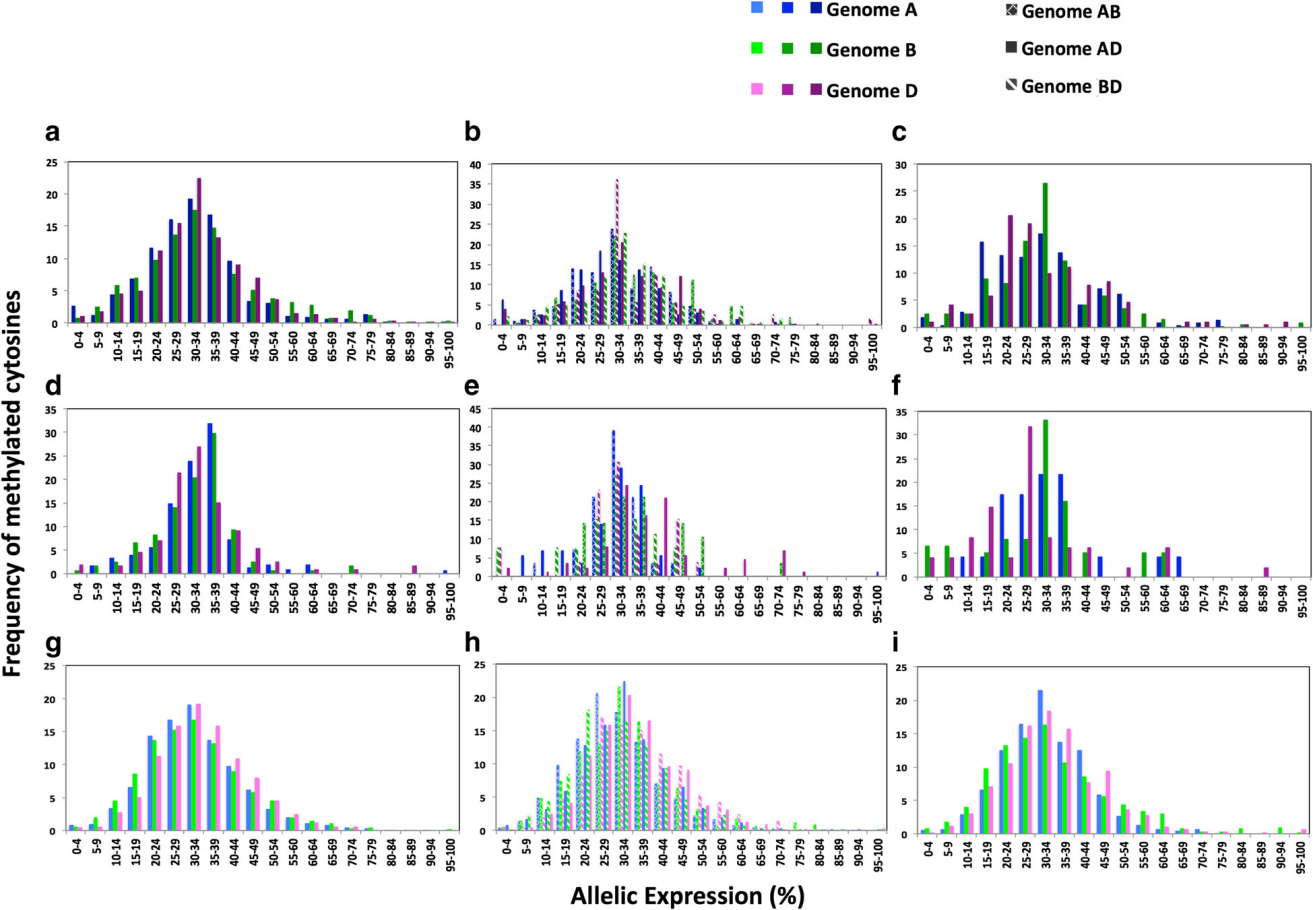
Histograms correlating gene expression with methylation. Allelic gene expression for each extended bait probe was normalized across the sub-genomes so that per site cumulative expression values for the individual A, B and D sub-genomes were all equal to 100 %. The *x*-axis is divided into 20 individual 5 % interval bins that correspond to allelic gene expression. The *y*-axis is the percentage of total counts for each respective bin, i.e., the frequency of methylated cytosine sites per sub-genome plotted against allelic gene expression for the extended bait sequence from which the site originated. This is shown for tri-methylated residues (**a, d, g**) bi-methylated residues (**b, e, h**) and uni-methylated residues (**c, f, i**) and data are further categorized as in non-transcribed regions associated closely with a gene (**a, b, c**), promoter regions (**d, e, f**) and transcribed regions (**g, h, i**)

To compare the impact of uni-, bi- and tri-genome methylation, we normalized allelic gene expression for each extended bait probe across the sub-genomes so that per site cumulative expression values for the individual A, B and D sub-genomes were all equal to 100 %, 0 % meaning a specific genome was not expressed at all and 100 % meaning that all expression for that region was from a single sub-genome. If the allelic expression was balanced between the three sub-genomes, we expect ~33 % expression. A histogram of methylated cytosine sites per sub-genome against allelic expression for the extended bait sequence that the site originated from is shown in Fig. 3. The data set was also subdivided into transcribed, promoter and non-transcribed regions. The distributions in Fig. 3 were tested to see if they fit a normal distribution using the Kolmogorov-Smirnov test (Table S8 in Additional file 1) and 94.4 % of the distributions fit this assumption (*p* < 0.01) with only AB bi-genome methylation being the exception (*p* = 0.200); the low number of residues in this dataset (28) may be responsible for this result.

In Fig. 3, for tri-genome methylated sites, the data uniformly tend towards a peak in the 30–35 % interval with an average allelic expression of 33.33 % (Fig. 3a, d, g). This shows a tendency towards equal contribution per sub-genome to overall expression at a position that is conserved across transcribed, non-transcribed and promoter regions. Notably, also in tri-genome methylated promoter regions (Fig. 3d) we see a loss of high allelic expression compared with transcribed regions with a 75^th^ percentile of expression of 37.84 versus 40.35 in transcribed regions and resultant a shift of the promoter-based expression distributions to be more leptokurtic (distribution kurtosis of 5.529 compared with 1.581 in transcribed regions). This loss of high expression is seen more markedly in uni-genome methylated promoter sites (Fig. 3f), where the 75^th^ percentile of expression is 35.73 versus 41.02 in transcribed regions. Looking further at promoter regions we see a significant drop in the average allelic expression correlating with uni- and bi-genome methylated genomes compared with tri-genome methylated genomes: 30.90 % (t = 2.4916, df = 1034, *p* = 0.0129) and 32.91 % (t = 0.5127, df = 1175, *p* = 0.6082), respectively. Therefore, uni-genome methylation in promoter regions correlates with a significant reduction in expression of the affected sub-genome (*p* < 0.05).

### Temperature has a small effect on gene methylation but is associated with stress responses

We could assay 293,076 cytosine residues across the three sub-genomes in both the 12 °C and 27 °C samples, of which 39,855 showed sub-genome methylation in one or both of the samples. Methylation changes could be identified at these sites between the two temperatures and differential methylation could be seen at 40 sites (0.1 %) in at least one sub-genome after sites that were considered differentially methylated between replicates were removed (Table S9 in Additional file 1). Only differential methylation between the samples that were conserved over the three replicate samples was considered. Of the 40 sites identified, two tri-genome differences, 11 bi-genome differences and 27 uni-genome differences were seen between the two samples. Variation in uni-genome methylation between the samples was most common, with four cases targeting sub-genome A, 13 targeting sub-genome B and 10 targeting sub-genome D. Twenty-seven sites could be directly associated with either temperature/stress sensitivity or methyltransferase activity, i.e., 77 % of those sites that could be annotated. Using the same method as previously described to identify DMRs, the two samples were compared and four DMRs were identified (minimum difference 25 %, q value < 0.01). Three DMRs were found between the samples in sub-genome B and one in genome D (Table S10 in Additional file 1).

RNA-seq data for the 12 °C and 27 °C samples was used for a comprehensive comparison of gene expression between the samples, i.e., to investigate if temperature-dependent, differential methylation correlated with gene expression. Following on from previous allelic expression level estimates, differential expression between the samples was quantified using probability of positive log ratio (PPLR) values calculated using BitSeq [21]. The PPLR values detail changes in expression levels between the 12 °C and 27 °C samples for each genome. A low PPLR indicates down-regulation of a sub-genome specific gene in the 27 °C sample compared with the 12 °C sample and a high PPLR value indicates up-regulation of the gene in the 27 °C sample compared with the 12 °C sample.

Here, 50,000 gene regions were analyzed per sub-genome and these were spread across 22,696 wheat genes. Comparing the two samples, 34,299 genome A gene regions, 34,595 genome B gene regions and 34,231 genome D gene regions were differentially expressed (69 % of analyzed regions), i.e., PPLR values deviated from the baseline 0.5 by ±20 % (≤0.4 or ≥0.6). These numbers are highly comparable between the sub-genomes: 1158 genome A, 1321 genome B and 1290 genome D genes were found to be highly differentially expressed, i.e., with a PPLR value <0.10 or >0.90.

Of the 40 sites that were differentially methylated between the 12 °C and 27 °C samples, 25 (63 %) from 20 gene regions were both differentially expressed and differentially methylated between the two samples; these show expression changes that could be linked to differential methylation (Table S11 in Additional file 1). In non-transcribed regions low methylation in the 27 °C sample resulted in a higher average expression than the 12 °C sample (18.02 % compared with 12.99 %, t = 0.6966, df = 12, *p* = 0.4994), while in transcribed regions it resulted in a lower average expression than the 12 °C sample (19.04 % versus 24.92 %, t = 0.9094, df = 10, *p* = 0.3845). Though these differences are not statistically significant, they fit the predicted model for decreased expression with methylation in non-transcribed regions versus increased expression with gene body methylation; therefore, methylation may have a role in gene expression control here but it is not solely responsible. Thirteen up-regulated genes in the 27 °C sample (Table S11 in Additional file 1) are linked to differential methylation and can be associated with GTP/ATPases, envelope proteins, small nuclear ribonucleoproteins and stress response, e.g., the MYB family proteins ATMYB1/2 [23] and promoter elements know to be involved in stress responses. Eight down-regulated genes in the 27 °C sample are linked to differential methylation and have been associated with stress and temperature sensitivity (Table S11 in Additional file 1), e.g., the two-component response regulator-like clock gene PRR95 known to effect clock function at high temperature [24].

Of the DMRs that were identified between the samples, 75 % could be linked to differential gene expression (Table S10 in Additional file 1); this is an increase on the 63 % of differentially methylated sites and the 69 % of regions in general showing gene expression changes demonstrating that DMRs are enriched for differential expression. These DMRs were a small group found in unknown/non-transcribed and transcribed regions only, although they could be indicative of larger DMRs in surrounding sequence that was not picked up by this analysis. Here the observed gene expression changes between samples are also relatively small, which is similar to the methylation changes that were observed and could be indicative of tissue specific methylation/gene expression change.

### Tri-genome methylation is highly conserved with a diploid wheat progenitor while sub-genome-specific methylation is more variable

To investigate the stability of methylation we investigated the methylation state of the likely diploid progenitor of the D sub-genome of wheat, *Ae. tauschii*, which is thought to have hybridized with the AABB progenitor approximately 10,000 years ago. We enriched, bisulfite-treated and sequenced *Ae. tauschii* DNA as described for Chinese Spring. After mapping, the *Ae. tauschii* sequence dataset had an average coverage of 27.9× across the extended bait reference sequence and mapped across 14 Mb. We sequenced 243,141 cytosine positions at 5× depth or more; 61,380 of these were in the 12 °C sample dataset, i.e., overlapping a homoeologous SNP that allows discrimination of sub-genome D for comparison in the hexaploid. *Ae. tauschii* was found to map to 3336 sites that were tri-genome methylated in the 12 °C hexaploid wheat sample. Looking at these positions, 4.8 % of the time *Ae. tauschii* methylation differed from that of hexaploid wheat. However, at the sites that showed uni-genome methylation in the 12 °C sample, and could be analyzed (247, 287 and 258 for sub-genomes A, B and D, respectively), we compared the methylation state of *Ae. tauschii.* We found 74.5 % of *Ae. tauschii* sites showed different methylation levels for sub-genome A-specific methylation, 73.9 % of sites did for sub-genome B-specific methylation and 13.6 % of sites did for sub-genome D-specific methylation. In summary, methylation that is conserved between the three sub-genomes is most highly conserved with the *Ae. tauschii* genome. However, while differences of only 4.8 % may seem small, if this was nucleotide variation, even this proportion would be considered large. Sub-genome D-specific methylation is also conserved with *Ae. tauschii*, at a lower level, and sub-genome A/B-specific methylation is largely non-conserved, although, surprisingly, a significant proportion of the A and B sub-genome-specific methylation is conserved with *Ae. tauschii*.

In addition to our analysis of methylation conservation, we also identified differences in methylation between hexaploid wheat and *Ae. tauschii*. Using the 61,380 cytosine sites that we mapped to *Ae. tauschii* and could also discriminate the sub-genomes in hexaploid wheat, we compared differences between each sub-genome and *Ae. tauschii.* For 7184 of these sites methylation was seen in one or more sub-genomes and/or *Ae. tauschii* (using standard thresholds)*.* For sub-genome D the methylation levels across these cytosine sites were compared with the profile for *Ae. tauschii* using Methylkit (minimum 50 % difference, q < 0.01). We identified 497 sites (6.9 % of those that were methylated) as having differences in methylation. Of these 497, 278 (55.9 %) showed a gain of methylation in sub-genome D and 219 (44.1 %) showed a loss of methylation in sub-genome D compared with *Ae. tauschii*. Additionally, sub-genome A- and B-specific methylation levels were compared with the profile for *Ae. tauschii* using the same methodology. More pronounced differential methylation was observed between the A and B sub-genomes of hexaploid wheat and *Ae. tauschii*: for sub-genome A, differences were seen at 1202 sites (16.7 %) with 51.8 % showing a gain of methylation in the A sub-genome and 48.2 % showing a loss; and for sub-genome B, differences were seen at 1180 sites (16.4 %) where 57.1 % showed a gain of methylation in the B sub-genome of hexaploid wheat and 42.9 % showed a loss. We see, as anticipated, that the methylation in *Ae. tauschii* is more closely conserved with sub-genome D than with sub-genome A or B. This is further highlighted by the Pearson correlation coefficient plots shown in Figure S11 in Additional file 1. GO terms that were enriched within the differentially methylated sites between sub-genome D and *Ae. tauschii* are detailed in Table S12 in Additional file 1, showing a focus on metabolism.

## Conclusions

The size and lack of reference sequence for the bread wheat genome has made it intractable to the comprehensive analysis of methylation patterns. Here we demonstrate the ability to analyze methylation across ~81 Mb of genic sequence, through a combination of sequence capture across the seven wheat chromosomes, bisulfite treatment and genome-specific assignment of reads using homoeologous SNPs. This allows us, for the first time, to address genome-wide methylation changes in this complex polyploid. We show that the degree and context of genome-wide methylation in wheat is comparable to that of rice, that methylated cytosines are evenly distributed along the chromosome’s genic sequence, that transposons are hyper-methylated and that the chloroplast genome is un-methylated. We identify sub-genome specific methylation and we demonstrate a correlation between promoter methylation and decreased expression. We found that the methylation conserved across the three sub-genomes of hexaploid wheat is most highly conserved in *Ae. tauschii* and that the methylation pattern in hexaploid wheat’s sub-genome D is more similar to that of *Ae. tauschii* than sub-genome A or B.

A key question in polyploids is whether genomes are differentially methylated. Allele-specific methylation has been observed in animals and plants and frequently linked to gene expression changes [8, 9]. Here, while the majority (45 %) of methylation we see in wheat is genome-independent, with all three genomes methylated, we do see considerable evidence for genome-specific methylation between the A, B and D sub-genomes with no one genome under selective methylation/de-methylation. We also show that transcribed regions had higher levels of sub-genome-specific non-CpG methylation than expected for diploid plants, suggesting a potential association of sub-genome specific methylation with pseudo-genes since this non-CpG methylation is more prevalent in non-transcribed regions.

In common with other plant polyploids, evidence for differential expression across the three sub-genomes has been seen in a number of studies, with as much as one-third of genes being ascribed to expression from a only one of the sub-genomes [25, 26]. Here, tri-genome methylation, which accounted for the majority of observed cytosine positions, correlates with equal expression at that position, i.e., equal expression levels across the three sub-genomes. Promoter regions show this tendency for equal contribution per sub-genome to overall expression at tri-genome methylated sites combined with a loss of top end allelic expression that is also seen at uni-genome methylated sites. We also found that uni-genome methylation correlated with a significant reduction in expression of the affected sub-genome compared with tri-genome methylated sites in promoter regions. In other organisms, links between allele-specific methylation and gene expression have been well documented [8, 9]. Genome-specific methylation accounts for 2.4 % of observed positions and 20 % of methylated cytosines and, as such, it may be important, although unlikely, to be responsible for all gene expression change. Furthermore, its influence may be masked when looking at genome-wide expression trends. Uni-genome expression was evenly distributed between the three sub-genomes, as was bi-genome expression, which, as an analysis across all plant tissues, is consistent with other investigations [22].

Population level variations in methylation patterns have been associated with disease [27] and environmental factors [28–30]. Here, we found only minor differences in methylation patterns between plants grown at 12 °C and 27 °C even though temperature had a dramatic effect on gene expression. However, in the few cases where we did see methylation, this was associated with small changes in gene expression and classes of genes that would be predicted to have a temperature-dependent expression profile, including heat shock and stress-related genes.

We found that tri-genome methylation sites are most highly conserved between hexaploid bread wheat and *Ae. tauschii*. Sub-genome D-specific methylation also showed similarity to *Ae. tauschii* with sub-genome A/B-specific methylation less conserved. This is anticipated since *Ae. tauschii* is the proposed donor of the D sub-genome. Interestingly, however, sub-genome D-specific methylation is conserved with *Ae. tauschii* at a lower level than tri-genome methylation, suggesting that tri-genome methylation is the most stable form of methylation. Furthermore, there was clear similarity between methylation patterns in *Ae. tauschii* and the A and B sub-genomes that was missing from the D sub-genome; this suggests that the hybridization between the AABB and D genome may have had significant impact on the A and B sub-genomes or that the A and B sub-genomes already had a similar methylation profile to *Ae. tauschii* that has since been lost from the D genome. It is clear that future methylome sequencing of the A and B sub-genome progenitors will be of great value.

We now have a technology to allow us to explore the role of methylation in an agriculturally important and complex polyploid genome. Wheat has a huge amount of genetic resources, including single genome progenitors, over 150,000 cultivars, phenotyped populations and modern elite cultivars with well-understood pedigrees. With the ability to characterize genome-wide patterns of methylation we can now address important biological questions, such as the role of epigenetics in the domestication of crops, the stability and long-term function of methylation in a polyploid genome and the interaction between epi-type and genotype.

## Materials and methods

### Design of the methylation enrichment system

The 6-Mb target sequence for this Agilent enrichment system was generated as per Figure S1 in Additional file 1. During selection of 120-mer RNA baits from the capture target sequence, previous mapping analyses of the wheat genic sequence were used to select fragments on the basis of previous good mapping coverage of the region, the presence of homoeologous SNPs and good genome-wide representation (determined using techniques and data derived from Winfield et al. [4] and Gardiner et al. [17]). The 120-mer baits were uploaded onto Agilent’s EArray (online custom microarray design tool) to allow submission for manufacture. Bait ‘boosting’ was selected to allow excess unused design space (less than 1 Mb in this case) to be filled with repeat sequences of baits that are predicted to perform less efficiently, i.e., those with an above average GC content are ‘boosted’ to ultimately gain even depth of sequence coverage across the target region.

### Preparation and mapping of bisulfite treated DNA samples

Six genomic DNA samples were enriched and sequenced. These samples were all extracted from the areal tissue of 7-day-old seedlings of the wheat variety Chinese Spring and included plants (B, C and D) grown at 12 °C and plants (B, C and D) grown at 27 °C. We sheared 3 μg of each sample for 6 × 60 s using a Covaris S2 focused-ultrasonicator (duty cycle 10 %, intensity 5, 200 cycles per burst using frequency sweeping). Fragmented DNA quality and quantity were assessed on a Bioanalyzer High Sensitivity DNA chip (Agilent) prior to purification using 1.8× Agencourt AMPure XP beads (Beckman Coulter). The six samples were enriched using custom SureSelect RNA oligomer baits. For this, end-repair, 3′ adenylation, methylated adapter ligation, hybridization, bisulfite conversion and PCR were carried out according to the SureSelect^XT^ Methyl-Seq Illumina Multiplexed Sequencing Protocol (version B, January 2013). Following bisulfite treatment, in order to determine the minimal number of PCR cycles required in the first amplification step, enriched libraries were quantified by quantitative PCR using the Agilent qPCR NGS Library Quantification Kit (for Illumina) on a Roche LightCycler 480 II System. Library concentrations were found to be between 200 and 400 pM and nine PCR cycles were therefore used. Amplified libraries were then indexed using six PCR cycles as per the standard protocol. Final libraries were pooled in equimolar amounts and sequencing was carried out on an Illumina HiSeq 2000, using version 3 chemistry, generating 2 × 100-bp paired-end reads.

The sequencing datasets for the samples were mapped to the extended bait sequence using Bismark, an aligner and methylation caller designed specifically for bisulfite-treated sequence data [11], a mismatch number of three was used and the non-directional nature of the library was specified. The Bismark methylation extractor tool was then used to identify all cytosine residues within the mapping and categorize the reads mapping to them as un-methylated or methylated at that position while also detailing which type of potential methylation site was present (CHH, CHG or CpG). The mapping results were also processed for SNP calling using the standard polyploid pipeline below.

### Standard mapping pipeline

For mapping analyses in this study, rather than mapping directly to the 6-Mb Agilent bait probe sequences, unless otherwise stated, data were mapped to the baits plus any surrounding contiguous DNA sequence that was available. This was because we used paired-end Illumina libraries which extended beyond the 120-mer baits. The total size of the mapping reference was ~44 Mb. All mapping analyses of non-bisulfite-treated samples were carried out using BWA [31] (version 0.7.4) using four mismatches per read. Mapping results were processed using SAMtools [32]; any non-uniquely mapping reads, unmapped reads, poor quality reads and duplicate reads were removed. SNP calling in diploid datasets was carried out using the GATK [33] Unified genotyper (after Indel realignment), which was used with a minimum quality of 50 and filtered using standard GATK recommended parameters, a minimum coverage of 5 and homozygous SNPs only selected. For polyploid datasets SAMtools mpileup was implemented with the SNP caller VarScan [34] to identify positions containing an alternative allele, with a minimum coverage of 5, an average mapping quality above 15 and a minor allele frequency (MAF) of greater than 0.1.

### Determining a reference homoeologous SNP list

Sequencing datasets representing the closest available diploid ancestor representatives for sub-genome A (*Triticum monococcum*), B (*Ae. speltoides*) and D (*Ae. tauschii*) [2, 35] were mapped individually to the extended sequence capture bait sequences to produce a list of positions at which all three sub-genome’s alleles were unambiguous and known, and at least one differed from the reference base, i.e., homoeologous SNPs. Sub-genome A data were generated on the Illumina GAIIx and the ~30-bp reads were mapped using the standard pipeline except only one mismatch per read was allowed. Sub-genome B data were generated on the Illumina GAIIx and the ~100-bp reads were mapped using the standard pipeline. Sub-genome D data were generated using SOLiD sequencing technology. The ~30-bp reads were mapped using the standard pipeline with use of parameters to allow mapping of reads in color-space. All mapping results were processed and SNP calling was carried out as per the standard diploid pipeline. Positions were identified at which all three sub-genomes' alleles were unambiguous and known, and at least one differed from the reference base and/or the other two sub-genomes, i.e., homoeologous SNPs. All C to T or G to A SNPs were excluded from this list to avoid future confusion between genuine SNP sites and C to T conversions of un-methylated cytosines as a result of the bisulfite conversion.

To complement this SNP list an additional homoeologous SNP list was generated. Three additional genomic DNA samples were extracted from the areal tissue of the same Chinese Spring 7-day-old seedlings that were grown at 12 °C. These were treated as per the previous DNA samples (i.e., enriched and sequenced), but bisulfite treatment was not used. The resultant sequencing reads were pooled for the three samples and mapped to the IWGSC wheat reference sequence using BWA [31]. Only reads that mapped perfectly (no mismatches or gaps) to one of the wheat sub-genomes were taken forward, i.e., genome assigned reads. Reads aligning perfectly to two genomes were also taken forward as bi-genome markers if both IWGSC contigs that they mapped to originated from the same chromosomal arm. These genome and bi-genome associated reads were then mapped to our extended bait sequence reference using BWA [31] and reads containing one mismatch but otherwise perfect alignment were selected. The genome assigned reads were thus aligned to this single reference sequence, which is representative of the three sub-genomes, allowing the discrimination of homoeologous SNP positions. SNP calling for polyploid datasets was carried out as previously described. Using genome assigned reads allowed us to match up the alleles at SNP locations with the contributing wheat sub-genome to define an additional homoeologous SNP list.

### Association of cytosine residues with the reference homoeologous SNP list

This technique is detailed in Figure S3 in Additional file 1. SNP positions were identified in the enriched hexaploid wheat bisulfite-treated sequencing dataset using the standard polyploid pipeline. Reads mapping to these SNP positions therefore have sufficient depth and average mapping quality overall and one or more alternative alleles present. Those positions that could also be found in the homoeologous SNP list were selected for further analysis, i.e., homoeologous SNPs within the treated data. Any sequencing read with a mapping quality over 20 and containing a cytosine residue methylation status calculated by Bismark plus a homoeologous SNP allele can be identified. Its SNP allele can be matched to a sub-genome, thus associating methylation status of that cytosine residue with a wheat sub-genome. For each cytosine position a summary of the number of reads hitting it for each sub-genome and whether or not these reads are methylated can be produced.

### Implementation of Methylkit

The software Methylkit was used to identify regions of differential methylation: our summary of each cytosine plus the number of reads hitting it per sub-genome and whether or not these reads are methylated can be used for such analysis. Firstly, variation was determined between the sample replicates to record ‘background methylation’ (e.g., between samples 12B, 12C, 12D and between samples 27B, 27C and 27D), i.e., sites containing noise that could easily be flagged and removed from downstream analyses. Replicates were used in pairwise comparisons and as such Fisher's exact test was used to discriminate statistically significant differences (q < 0.01 and methylation difference of ≥50 %). Methylkit was implemented in the same way, after replicate sample pooling, to define differential methylation between the sub-genomes of wheat, within and between samples, using pairwise comparisons. Differences were recorded between one genome and the other two (uni-genome methylation if the site also met the standard threshold for methylation) and vice versa (bi-genome methylation). Differences between all three genomes could then be determined, whereas, between samples, pairwise comparisons were between sub-genome A-A, B-B and D-D. Due to higher sample coverage after pooling, differences were called at q < 0.01 and a methylation difference of ≥25 %. Finally, after summarizing methylation per extended bait probe, Methylkit could be implemented with pairwise comparisons to define DMRs between the sub-genomes of wheat and between the two samples (q < 0.01 and methylation difference of ≥25 %).

MethylKit was used to define differential methylation between the ‘older’ Chinese Spring and the 12 °C sample using pairwise comparisons and a methylation difference of 50 % (q < 0.01) due to the lower coverage in the ‘older’ Chinese Spring dataset. The same method was used for the *Ae. tauschii* dataset.

### Validation of homoeologous SNP calls and methylation status from next generation sequencing data using Sanger sequencing

The DNA for the 12 °C and 27 °C samples was bisulfite-treated using the EZ DNA Methylation-Gold kit (Zymo research group). Twenty-three SNP sites were selected at random for validation and primers were designed to capture 150–400-bp regions surrounding the SNP sites in bisulfite-treated DNA. These regions contained a total of 337 analyzed cytosine sites (304 un-methylated and 33 partially or fully methylated) that could also be used for methylation site status validation. For primer design all Cs were treated as Ys (C/T) and no more than two Ys were included in a primer sequence. PCR amplification of the DNA followed using KAPA HiFi HotStart Uracil + ReadyMix and finally samples were sequenced using Sanger sequencing (Source Bioscience). If SNP alleles that had been seen in the next generation sequencing data could be seen in the Sanger sequencing data in approximately the same proportions (within ~20 %), they were said to be validated. Similarly, a methylation call was deemed to be correct if the Sanger sequencing data showed a proportion of methylation that was within approximately 20 % of that seen previously, i.e., 50 % methylation would mean approximately equal peaks of C and T in the Sanger sequencing data. All of the SNP validations for the 12 °C sample are shown in Figure S5 in Additional file 1. This SNP analysis was coupled to the analysis of the cytosine residues surrounding the SNP call. Table S2 in Additional file 1 details the positions of these residues and their expected and observed methylation statuses, demonstrating the high degree of accuracy of calls generated within this study.

### Calculation of bisulfite conversion rate

Bisulfite conversion rates can be measured by mapping reads to the chloroplast genome, which is un-methylated [36, 37]. While we did not enrich for chloroplast DNA, because we used total wheat DNA, a proportion of our off-target sequences mapped to the wheat chloroplast genome. With our off-target DNA we were able to map to 99.73 % of the chloroplast genome with an average coverage of ~114× and 98.92 % of the cytosine bases in the sequencing reads were bisulfite converted.

### Identifying transcribed and non-transcribed regions in the extended bait sequence

The 44-Mb extended bait sequence was used as a query in a BLAST [38] alignment against the IWGSC wheat reference sequence. Aligned regions with an E-value less then 1e-5, over 90 % sequence identity and a length of greater than 100 bp were taken forward. Of the 50,000 reference contigs, 46,756 (94 %) had hits and correlation of these hits with the MIPS gene annotations (v2.2) allowed identification of exon and intron or transcribed regions. Promoter-associated regions were predicted as sequence up to 2000 bp upstream of the 5’ untranslated region or transcription start site. If no gene association was found, regions were labeled as unknown/potentially non-transcribed.

### Setting of thresholds for identification of differential methylation

Within this study homoeologous SNPs were used to associate sequencing reads with individual wheat sub-genomes. Due to the density of SNPs, we have regions where we can define the three sub-genomes unambiguously. Individually, however, homoeologous SNPs typically define only two SNP alleles; e.g., a sub-genome A SNP would define reads for the A sub-genome and for the remaining two sub-genomes, B and D, together, although, proportionally, read numbers tend to double for the allele representing the two sub-genomes. This is expected since the bisulfite treatment renders the discrimination of C-to-T and G-to-A SNPs impossible. We can only confidently categorize reads that are an average of two sub-genomes into highly methylated (i.e., both genomes likely to be methylated) or low level methylation (i.e., both genomes likely to be un-methylated) and this formed the reasoning for developing our own thresholds to categorize methylation in wheat. However, it means that intermediate level methylation, which is likely to be associated with tissue-specific regulation, was not fully described and is beyond the scope of this study. We adjusted thresholds that were used in a genome-wide analysis of *Arabidopsis* where CpG sites were called methylated at 80–100 % methylation, CHG sites at over 25 % and CHH sites at levels over 10 % [7]. Thresholds of ≥75 % and ≤25 % methylation were used to categorize the CpG data as methylated or un-methylated. At CHG and CHH sites >95 % of methylation fell below the 10 % threshold and, as such, methylation exceeding a threshold of 10 % was likely to denote a highly methylated residue, or two methylated genomes at positions that are an average of two sub-genomes, with 0 % methylation denoting two un-methylated genomes. This scoring allows the analysis of positions that were assigned to a genome pair in addition to those that were assigned to a single genome.

At each cytosine residue site, where sub-genomes can be discriminated, the percentage of the reads mapping to each sub-genome that were methylated can be calculated using Bismark’s [11] categorization of sequencing reads as methylated or un-methylated at each cytosine residue. Under the same rationale differential methylation was identified between sub-genomes and/or samples at a minimum difference of 50 % to ensure elimination of replicate variance and the analysis of genuine methylation changes.

### Construction of pseudo-chromosomes from capture design contigs

We made use of 21 wheat chromosomal pseudo-molecules that were created by organizing and concatenating the IWGSC CSS assemblies using POPSEQ data [18]. BLASTN [38] was used to place the capture design contigs onto these chromosomal pseudo-molecules (E-value cutoff 1e-5, minimum sequence identity 90 and minimum length of 100 bp). Relative positions for the capture design contigs along the chromosomal pseudo-molecules could then be used to order them into our POPSEQ based pseudo-chromosomes. We desired seven POPSEQ based pseudo-chromosomes, as per our capture probe set, that were representative of the 21 wheat chromosomes. This would in effect align the three wheat genomes to allow comparison directly. Therefore, the order of the capture design contigs along genome B’s chromosomal pseudo-molecules 1–7 was preferentially utilized since the greatest number of contigs could be aligned to these sequences and therefore included (83 %).

### GOEAST analysis

The online tool GOEAST (Gene Ontology Enrichment Analysis Software Toolkit) was implemented (customized microarray analysis) using a background file of all methylated sites under analysis and their GO annotations compared individually with each of the files of uni-genome A, B and D methylation sites. Default parameters were used and enriched terms were filtered out if they had a *p* value <0.05. The previous alignment of the extended bait probes with the IWGSC reference sequence and MIPS gene annotations, including GO term annotation, allowed easy assignment of GO terms to methylation site regions.

### Generation of gene expression data for the 12 °C and 27 °C sample for differential gene expression analysis

A FASTA file of reference sequences was created from the extended bait sequences. The reference homoeologous SNP list was implemented to develop three sequences for each probe representing the A, B and D sub-genomes. Bowtie2 [20] was used to map the paired-end RNA-seq reads to this reference. These reads were generated for the same two wheat samples, 12 °C and 27 °C, using the Illumina HiSeq using largely the same methods that were implemented for the genomic DNA with the exception of the enrichment step. Reads were permitted to map to multiple locations (up to 101) and all other parameters were left as default. Allelic expression levels were estimated for each sample using stage one of BitSeq [21], without the assumption of uniform read distribution. The limitA parameter was set at 100 in the parseAlignment step, telling BitSeq to ignore any reads that had aligned more than 100 times. This parameter was set as one lower than the maximum number of alignments permitted when aligning reads. This was done due to the way Bowtie2 reports multiple alignments; it reports the first alignments found if it reaches the maximum number of alignments permitted, which do not necessarily include all of the best alignments. Stage two of BitSeq was then used to identify differential expression between plants grown at 12 °C and 27 °C, on the basis of PPLR values.

### Availability of supporting data

All sequencing datasets plus our constructed pseudo-genome are available (study PRJEB8762) from the European Nucleotide Archive (http://www.ebi.ac.uk/ena/data/view/PRJEB8762). Our constructed pseudo-genome is also available on request via iPLant (http://www.ebi.ac.uk/ena/data/view/PRJEB8762).

### Ethics approval

Ethics approval was not needed for this study.

## Additional file

Additional file 1:
**Supplementary data file includes Figures S1–S11, Tables S1–S12 and Notes 1–4.** (PDF 107087 kb)

## Abbreviations

bp: base pair
DMR: differentially methylated region
GO: Gene Ontology
IWGSC: International Wheat Genome Sequencing Consortium
PCR: polymerase chain reaction
PPLR: probability of positive log ratio
SNP: single-nucleotide polymorphism

## References

[1] Marcussen T, Sandve SR, Heier L, Spannagl M, Pfeifer M, International Wheat Genome Sequencing Consortium (2014). Ancient hybridizations among the ancestral genomes of bread wheat. Science..

[2] Brenchley R, Spannagl M, Pfeifer M, Barker GL, D'Amore R, Allen AM (2012). Analysis of the bread wheat genome using whole-genome shotgun sequencing. Nature..

[3] Saintenac C, Jiang D, Akhunov ED (2011). Targeted analysis of nucleotide and copy number variation by exon capture in allotetraploid wheat genome. Genome Biol..

[4] Winfield MO, Wilkinson PA, Allen AM, Barker GL, Coghill JA, Burridge A (2012). Targeted re-sequencing of the allohexaploid wheat exome. Plant Biotechnol J..

[5] Finnegan EJ, Genger RK, Peacock WJ, Dennis ES (1998). DNA methylation in plants. Annu Rev Plant Phys..

[6] Zhang X, Yazaki J, Sundaresan A, Cokus S, Chan SW, Chen H (2006). Genome-wide high-resolution mapping and functional analysis of DNA methylation in arabidopsis. Cell..

[7] Cokus SJ, Feng S, Zhang X, Chen Z, Merriman B, Haudenschild CD (2008). Shotgun bisulphite sequencing of the Arabidopsis genome reveals DNA methylation patterning. Nature..

[8] Fang F, Hodges E, Molaro A, Dean M, Hannon GJ, Smith AD (2012). Genomic landscape of human allele-specific DNA methylation. Proc Natl Acad Sci U S A..

[9] Chodavarapu RK, Feng S, Ding B, Simon SA, Lopez D, Jia Y (2012). Transcriptome and methylome interactions in rice hybrids. Proc Natl Acad Sci U S A..

[10] Darst RP, Pardo CE, Ai L, Brown KD, Kladde MP. Bisulfite sequencing of DNA*.* Wiley; 2001. doi:10.1002/0471142727.mb0709s91. 10.1002/0471142727.mb0709s91 PMC3214597 20583099

[11] Krueger F, Andrews SR (2011). Bismark: a flexible aligner and methylation caller for Bisulfite-Seq applications. Bioinformatics..

[12] Akalin A, Kormaksson M, Li S, Garrett-Bakelman FE, Figueroa ME, Melnick A (2012). methylKit: a comprehensive R package for the analysis of genome-wide DNA methylation profiles. Genome Biol.

[13] Genereux DP, Johnson WC, Burden AF, Stöger R, Laird CD (2008). Errors in the bisulfite conversion of DNA: modulating inappropriate- and failed-conversion frequencies. Nucleic Acids Res..

[14] Li X, Zhu J, Hu F, Ge S, Ye M, Xiang H (2012). Single-base resolution maps of cultivated and wild rice methylomes and regulatory roles of DNA methylation in plant gene expression. BMC Genomics..

[15] Widman N, Jacobsen SE, Pellegrini M (2009). Determining the conservation of DNA methylation in Arabidopsis. Epigenetics..

[16] Zheng Q, Wang XJ (2008). GOEAST: a web-based software toolkit for Gene Ontology enrichment analysis. Nucleic Acids Res..

[17] Gardiner L-J, Gawroński P, Olohan L, Schnurbusch T, Hall N, Hall A (2014). Using genic sequence capture in combination with a syntenic pseudo genome to map a deletion mutant in a wheat species. Plant J..

[18] Chapman JA, Mascher M, Buluç A, Barry K, Georganas E, Session A (2015). A whole-genome shotgun approach for assembling and anchoring the hexaploid bread wheat genome. Genome Biol..

[19] Stockwell PA, Chatterjee A, Rodger EJ, Morison IM (2014). DMAP: differential methylation analysis package for RRBS and WGBS data. Bioinformatics..

[20] Langmead B, Salzberg SL (2012). Fast gapped-read alignment with Bowtie 2. Nat Methods..

[21] Glaus P, Honkela A, Rattray M (2012). Identifying differentially expressed transcripts from RNA-seq data with biological variation. Bioinformatics..

[22] Pfeifer M, Kugler KG, Sandve SR, Zhan B, Rudi H, Hvidsten TR (2014). Genome interplay in the grain transcriptome of hexaploid bread wheat. Science..

[23] Urao T, Yamaguchi-Shinozaki K, Urao S, Shinozaki K (1993). An Arabidopsis myb homolog is induced by dehydration stress and its gene product binds to the conserved MYB recognition sequence. Plant Cell..

[24] Salomé PA, Weigel D, McClung CR (2010). The role of the Arabidopsis morning loop components CCA1, LHY, PRR7, and PRR9 in temperature compensation. Plant Cell..

[25] Leach LJ, Belfield EJ, Jiang C, Brown C, Mithani A, Harberd NP (2014). Patterns of homoeologous gene expression shown by RNA sequencing in hexaploid bread wheat. BMC Genomics..

[26] Akhunova AR, Matniyazov RT, Liang H, Akhunov ED (2010). Homoeolog-specific transcriptional bias in allopolyploid wheat. BMC Genomics..

[27] Langevin SM, Kelsey KT (2013). The fate is not always written in the genes: Epigenomics in epidemiologic studies. Environ Mol Mutagen..

[28] Feil R, Fraga MF (2012). Epigenetics and the environment: emerging patterns and implications. Nat Rev Genet..

[29] Meyer P. Epigenetic variation and environmental change. J Exp Bot. 2015. doi:10.1093/jxb/eru502. 10.1093/jxb/eru502 25694547

[30] Hashida SN (2006). The temperature-dependent change in methylation of the Antirrhinum transposon Tam3 is controlled by the activity of its transposase. Plant Cell..

[31] Li H, Durbin R (2009). Fast and accurate short read alignment with Burrows-Wheeler transform. Bioinformatics..

[32] Li H, Handsaker B, Wysoker A, Fennell T, Ruan J, Homer N (2009). The Sequence Alignment/Map format and SAMtools. Bioinformatics..

[33] McKenna A, Hanna M, Banks E, Sivachenko A, Cibulskis K, Kernytsky A (2010). The Genome Analysis Toolkit: A MapReduce framework for analyzing next-generation DNA sequencing data. Genome Res..

[34] Koboldt DC, Zhang Q, Larson DE, Shen D, McLellan MD, Lin L (2012). VarScan 2: Somatic mutation and copy number alteration discovery in cancer by exome sequencing. Genome Res..

[35] Wilkinson PA, Winfield MO, Barker GL, Allen AM, Burridge A, Coghill JA (2012). CerealsDB 2.0: an integrated resource for plant breeders and scientists. BMC Bioinformatics..

[36] Lister R, O'Malley RC, Tonti-Filippini J, Gregory BD, Berry CC, Millar AH (2008). Highly integrated single-base resolution maps of the epigenome in Arabidopsis. Cell..

[37] Fojtová M, Kovařík A, Matyášek R (2001). Cytosine methylation of plastid genome in higher plants. Fact or artefact?. Plant Sci.

[38] Altschul SF, Gish W, Miller W, Myers EW, Lipman DJ (1990). Basic local alignment search tool. J Mol Biol..

