# Supplementary material for: A genome-wide survey of DNA methylation in hexaploid wheat
Source: Genome Biol. 2015 Dec 10;16:273. doi: 10.1186/s13059-015-0838-3 (PMC4674939; doi:10.1186/s13059-015-0838-3)
Supplement: Additional file 1: — Supplementary data file includes Figures S1–S11, Tables S1–S12 and Notes 1–4. (PDF 107087 kb) [file 13059_2015_838_MOESM1_ESM.pdf]

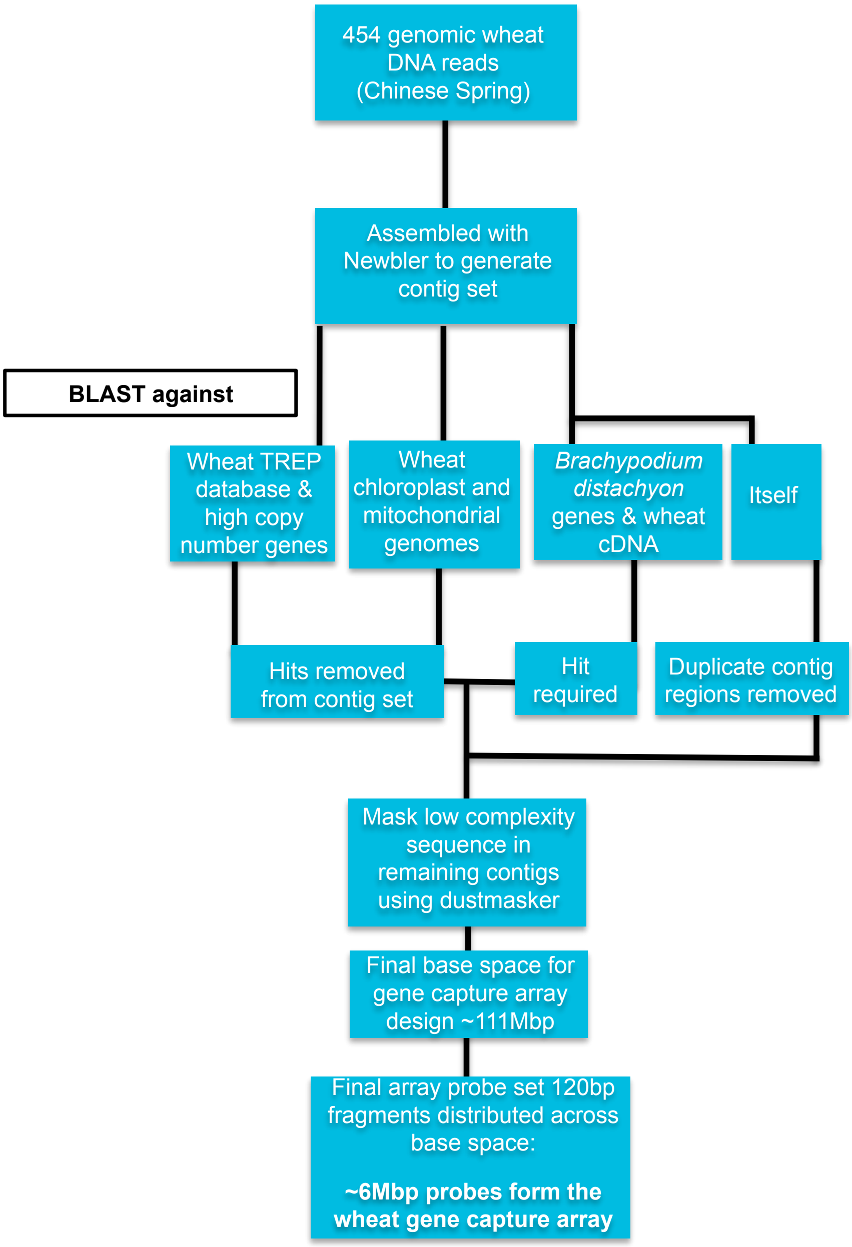

Figure 1. Design of the wheat gene capture array Sequencing reads were assembled into contigs using the software Newbler, contigs were selected that hit genes in the closely related species *Brachypodium distachyon* and that hit the wheat cDNA sequence when BLASTN[1] searches were conducted. These contigs were then processed to eliminate redundancy, chloroplast and mitochondrial sequence and repetitive sequence (TREP database). The remaining contigs were filtered for regions of low complexity and used as the design target sequence for the array (~111Mbp). The RNA baits for this SureSelect Methyl-Seq Target Enrichment system are all 120bp in length, unique, non-repetitive and are evenly placed across the available wheat genic target sequence.

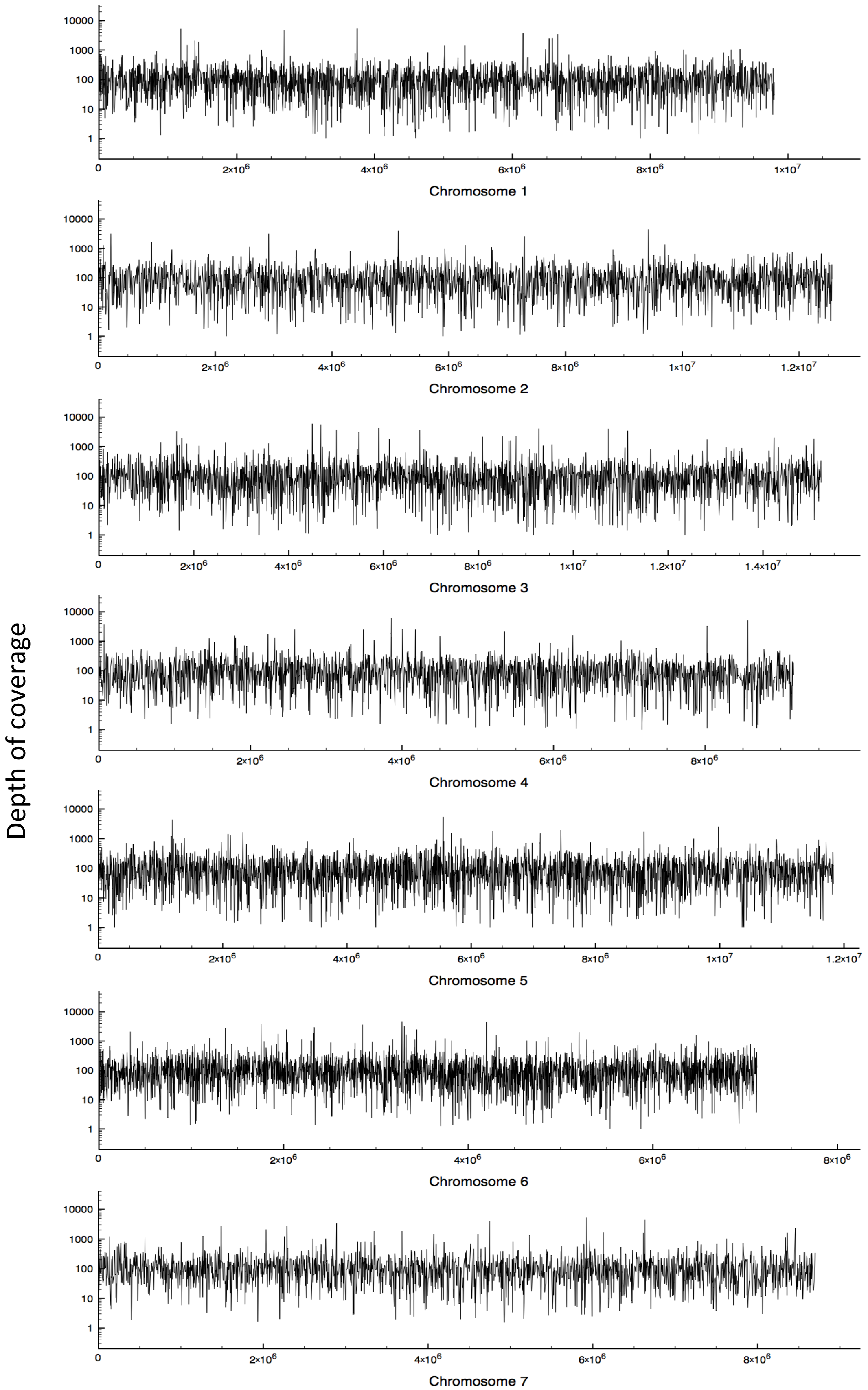

Figure 2. Average depth of coverage per bait probe plotted for the 12°C sample. Coverage plotted along each pseudo-chromosome construct after ordering and concatenation of extended capture array bait sequences (see methods).

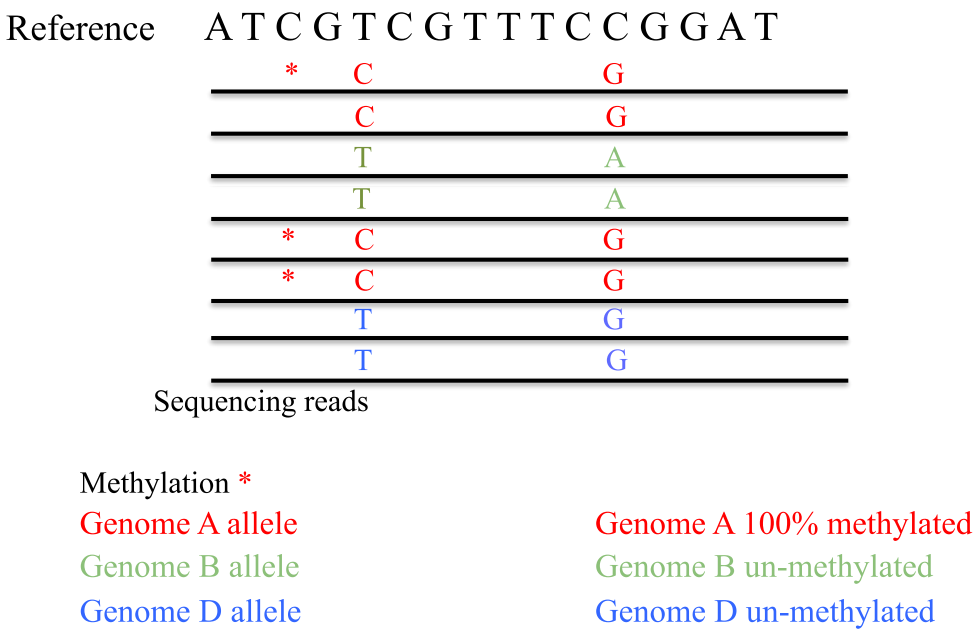

Figure 3. Association of methylation sites with the 3 wheat sub-genomes. Illustrating the use of association of two bi-allelic homoeologous SNPs: one C->T discriminating genome A from B and D and another A->G discriminating genome B from A and D. This ultimately allows discrimination of all three sub-genomes due to their combination of SNP alleles. Here a methylation site is shown within the same sequencing read on sub-genome A to outline the determination of its genome of origin.

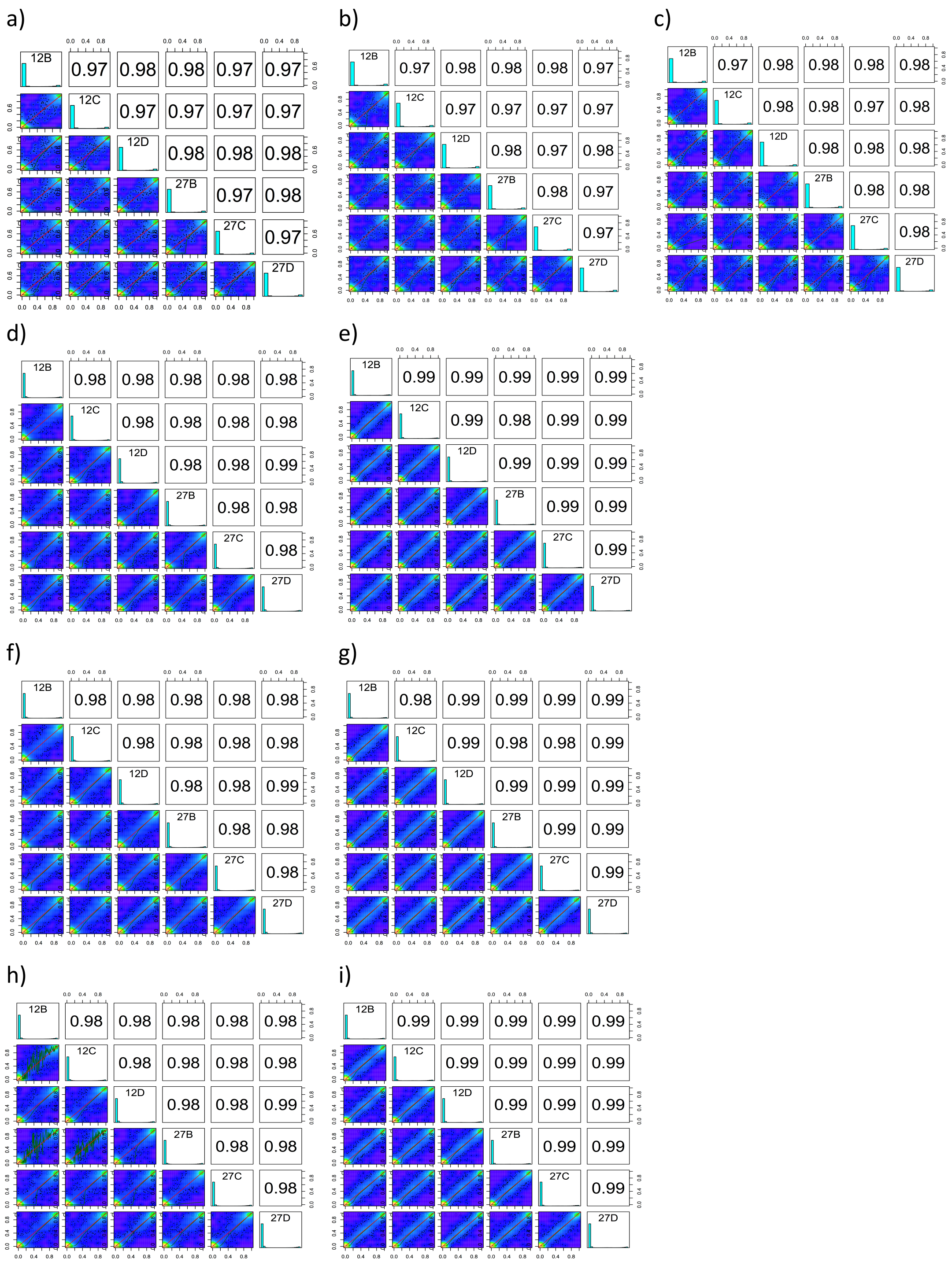

Figure 4. MethylKit[2] Pearson correlation coefficient computations to compare methylation across the three replicates (B, C and D) of the 12°C sample and the 27°C sample. Figures demonstrate comparisons of methylation levels across the respective samples at positions that have information that is associated with the a) A sub-genome, b) B sub-genome and the c) D sub genome directly. Where bi-allelic homoeologous SNPs have been used to assign methylation levels, at a position, to one genome or the other two the following is shown; information associated with d) A sub-genome and e) BD sub-genomes or f) B sub-genome and g) AD sub-genomes or h) D sub-genome and i) AB sub-genomes.

Sample 12: Validation of SNP and methylation sites in CONTIG318298_1-1640-953 between positions 951 and 1190

SNP position 1073
In NGS see; G:64% A:36%
We see here; ~100% G

SNP position 1049
In NGS see; ~100% T
We see here; ~100% T ✔

SNP position 982
In NGS see; G:83% A:17%
We see here; ~98% G negligible 1-2% A ✔

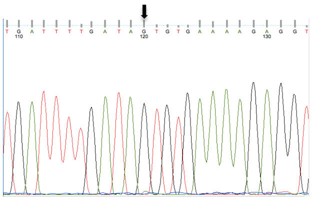

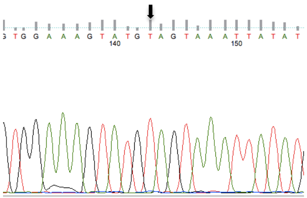

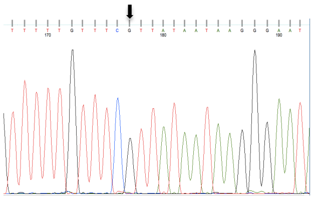

Sample 27: Validation of SNP and methylation sites in CONTIG318298_1-1640-953 between positions 951 and 1190

SNP position 1073
In NGS see; G:65% A:35%
We see here; ~90% G ~10% A

SNP position 1049
In NGS see; ~100% T
We see here; ~100% T ✔

SNP position 982
In NGS see; G:82% A:18%
We see here; ~98% G negligible 1-2% A ✔

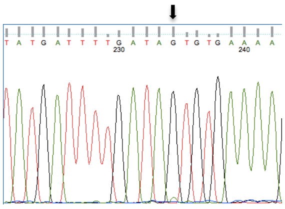

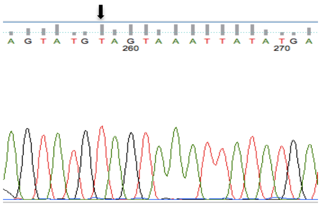

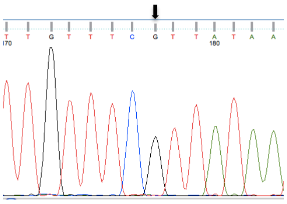

Sample 12: Validation of SNP and methylation sites in CONTIG44098_1040-2994-1412 between positions 1374 and 1684

SNP position 1420
In NGS see; T:70% C:30%
See here; T ~50% C ~50% ✔

SNP position 1528
In NGS see; C:57% T:43%
See here; C ~ 50% T ~ 50% ✔

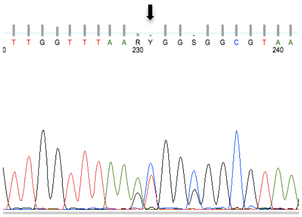

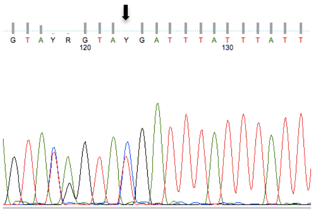

Sample 27: Validation of SNP and methylation sites in CONTIG44098_1040-2994-1412 between positions 1374 and 1684

SNP position 1420
In NGS see; T:82% C:18%
See here; T ~50% C ~50%

SNP position 1528
In NGS see; C:61% T:39%
See here; C ~ 50% T ~ 40% ✔

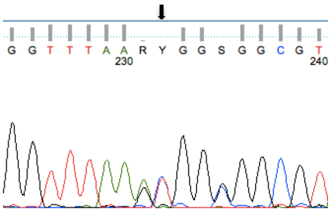

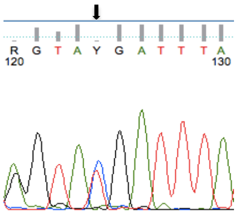

Sample 12: Validation of SNP and methylation sites in CONTIG248041_1-1793-1442 between positions 1387 and 1525

SNP position 1524
In NGS see; T:100%
We see here; ~100% T ✔

SNP position 1466
In NGS see; T:100%
We see here; ~100% T ✔

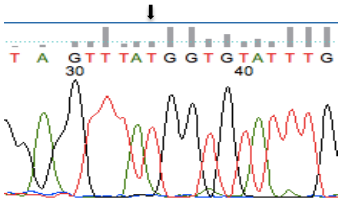

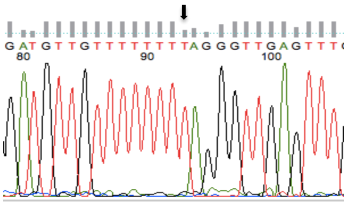

Sample 27: Validation of SNP and methylation sites in CONTIG248041_1-1793-1442 between positions 1387 and 1525

SNP position 1524
In NGS see; T:100%
We see here; ~100% T ✔

SNP position 1466
In NGS see; T:99% C:1%
We see here; ~100% T ✔

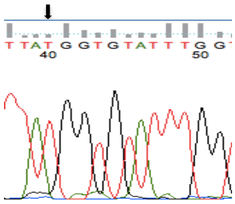

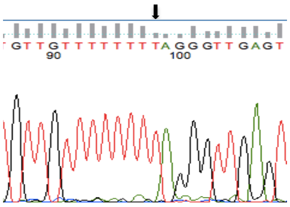

Sample 12: Validation of SNP and methylation sites in CONTIG174481_1-2021-596 between positions 668 and 887

SNP position 681
In NGS see; 92% T 8% A
We see here; ~100% T ✔

SNP position 716
In NGS see; 91% A 9% T
We see here; ~99% A ~1% T ✔

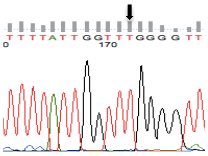

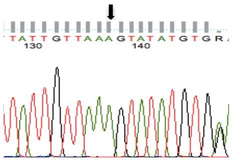

Sample 27: Validation of SNP and methylation sites in CONTIG174481_1-2021-596 between positions 668 and 887

SNP position 681
In NGS see; 92% T 8% A
We see here; ~100% T ✔

SNP position 716
In NGS see; 91% A 9% T
We see here; ~98% A ~2% T ✔

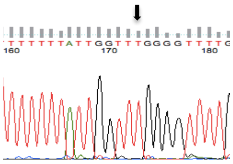

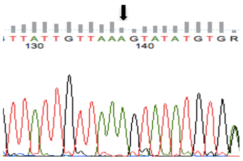

Sample 12: Validation of SNP and methylation sites in CONTIG381103_1-1538-1308 between positions 1145 and 1290

SNP position 1195
In NGS see; 83% A 17% T
We see here; ~80%A % ~20%T ✔

SNP position 1259
In NGS see; 46% T 54% C
We see here; ~50% T ~50% C ✔

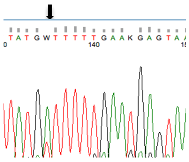

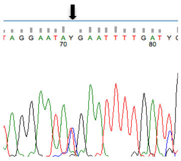

Sample 27: Validation of SNP and methylation sites in CONTIG381103_1-1538-1308 between positions 1145 and 1290

SNP position 1195
In NGS see; 66% A 34% T
We see here; ~70%A % ~30%T ✔

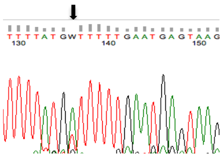

SNP position 1259
In NGS see; 42% T 58% C
We see here; ~50% T ~50% C ✔

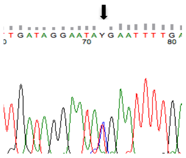

Sample 12: Validation of SNP and methylation sites in CONTIG256716_1-1768-36 between positions 79 and 423

SNP position 102
In NGS see; 29% A 71% G
We see here; ~30%A % ~70%G ✔

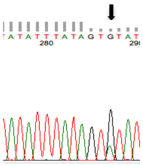

SNP position 125
In NGS see; 100% A
We see here; ~100% A ✔

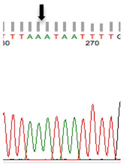

SNP position 139
In NGS see; 100% A
We see here; ~ 100% A ✔

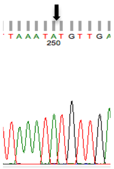

SNP position 215
In NGS see; 100% A
We see here; ~100% A ✔

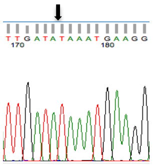

SNP position 306
In NGS see; 33% G 67% A
We see here; ~70% A ~30% G ✔

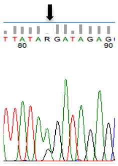

Sample 27: Validation of SNP and methylation sites in CONTIG256716_1-1768-36 between positions 79 and 423

SNP position 102
In NGS see; 29% A 71% G
We see here; ~30%A % ~70%G ✔

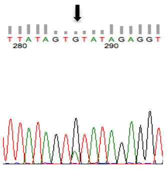

SNP position 125
In NGS see; 100% A
We see here; ~100% A ✔

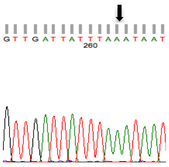

SNP position 139
In NGS see; 100% A
We see here; ~ 100% A ✔

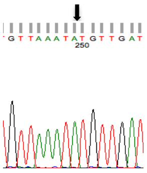

SNP position 215
In NGS see; 100% A
We see here; ~100% A ✔

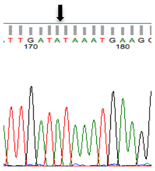

SNP position 306
In NGS see; 33% G 67% A
We see here; ~70% A ~30% G ✔

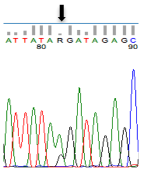

Sample 12: Validation of SNP and methylation sites in CONTIG1173474_1-941-195 between positions 217 and 503

SNP position 315
In NGS see; 69% A 31% T
We see here; ~100%A

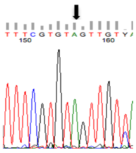

SNP position 275
In NGS see; 28% T 72% A
We see here; ~95% A ~5% T

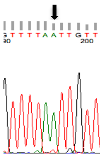

Sample 27: Validation of SNP and methylation sites in CONTIG1173474_1-941-195 between positions 217 and 503

SNP position 315
In NGS see; 69% A 31% T
Expect; ~69% A ~31%T
We see here; ~100%A

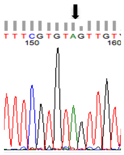

SNP position 275
In NGS see; 29% T 71% A
Expect; 29-0%G: 71-100% A:
We see here; ~95% A ~5% T

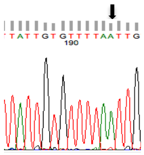

Sample 12: Validation of SNP and methylation sites in CONTIG796042_275-1136-650 between positions 436 and 806

SNP position 686
In NGS see; 25% A 75% T
We see here; ~95%T ~5% A (consistent C noise) ✔

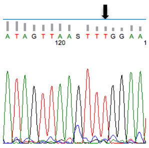

Sample 27: Validation of SNP and methylation sites in CONTIG796042_275-1136-650 between positions 436 and 806

SNP position 686
In NGS see; 36% A 64% T
We see here; ~90%T ~10% A (consistent C noise)

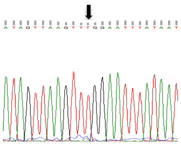

Sample 12: Validation of SNP and methylation sites in CONTIG462845_1-1429-894 between positions 893 and 1115

SNP position 930
In NGS see; 57% T 43% C
We see here; ~60%T ~40% C ✔

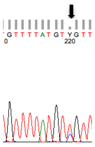

SNP position 1014
In NGS see; 33% T 67% A
We see here; ~70% A ~30% T ✔

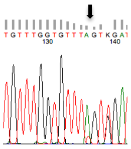

Sample 27: Validation of SNP and methylation sites in CONTIG462845_1-1429-894 between positions 893 and 1115

SNP position 930
In NGS see; 51% T 49% C
We see here; ~53%T ~47% C ✔

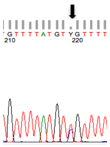

SNP position 1014
In NGS see; 30% T 70% A
We see here; ~70% A ~30% T ✔

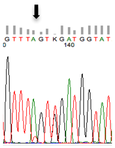

Sample 12: Validation of SNP and methylation sites in CONTIG643204_1-1170-857 between positions 678 and 761

SNP position 762
In NGS see; 69% A 31% G
We see here; ~50%A ~50% G ✔

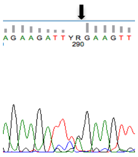

SNP position 715
In NGS see; 100% A
We see here; ~100% A ✔

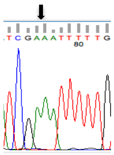

Sample 27: Validation of SNP and methylation sites in CONTIG643204_1-1170-857 between positions 678 and 761

SNP position 762
In NGS see; 57% A 43% G
We see here; ~50%A ~50% G ✔

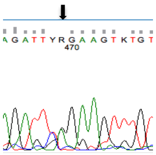

SNP position 715
In NGS see; 100% A
We see here; ~100% A ✔

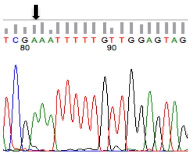

Figure 5. Sanger sequencing output traces. Raw Sanger sequencing outputs generated from bisulfite treated DNA for 23 SNPs in each of sample 12 and 27. SNPs were originally identified in the next generation sequencing data that was derived from enriched bisulfite treated DNA and sites extend across 10 of the methylation array extended bait probe sequences. This data outlines the validation of these SNP calls.

Stage	Feature	Proportion Transcribed (intron/exon)
Stage 1<br>Reference & SNP list	Reference sequence 44Mb<br><br>72,345 Homeologous SNPs	55%<br><br>78%
Stage 2<br>Within mapped sequencing data	7,813,105 cytosine residues (8,069,906)<br><br>33,650 Homeologous SNPs (33,912)	51% (51%)<br><br>79% (79%)
Stage 3<br>Associated data	1,001,620 cytosine residues associated with a homeologous SNP (1,018,531)	78% (78%)
Stage 4<br>Final dataset:<br>3 sub-genomes mapped 5x or more	318,452 cytosine residues (324,227)<br><br>28,632 with SNPs for A, B and D sub-genome (29,202)<br><br>289,820 with SNPs for A/BD, B/AD or D/AB (295,025)	80% (80%)<br><br>85% (85%)<br><br>80% (80%)

Figure 6. Determination of the subset of data that was available for detailed analysis in the 12°C sample. Corresponding numbers in the 27°C sample shown in brackets. At each stage the percentages of sites that are transcribed/non-transcribed are detailed. **Stage 1** describes the mapping reference and the reference homoeologous SNP list. **Stage 2** Shows all cytosine residues and reference homoeologous SNP positions within the mapped 12°C and 27°C sample datasets. To assign a cytosine residue to the A, B or D wheat sub-genome an individual sequencing read must contain the cytosine residue plus a homoeologous SNP allele; In **Stage 3** the outputs from **stage 2** are combined to identify all cytosines that could be associated with homoeologous SNP locations in each of the datasets. **Stage 4** outlines the final analyzable dataset; cytosine residues were selected if all three genomes could each be identified at a depth of 5X or more in the mapped reads.

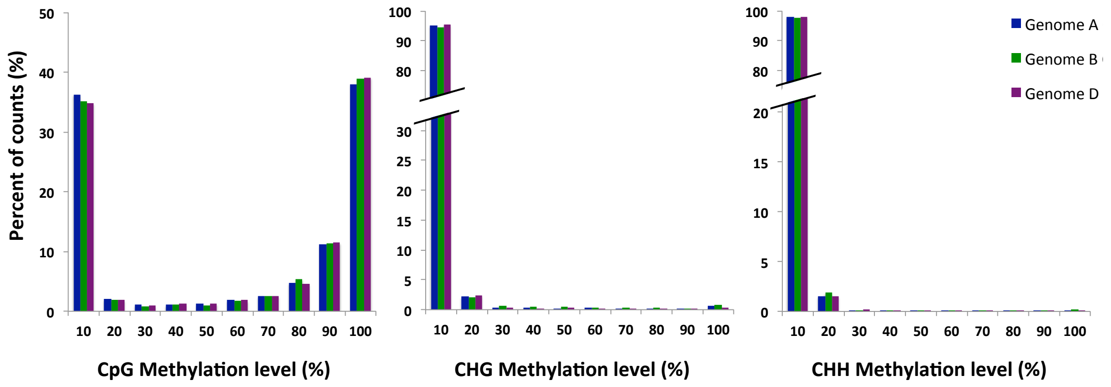

Figure 7. General patterns of methylation in the Wheat genome. a-c, Distribution of methylation percentage of (**a**) CpG, (**b**) CHG, and (**c**) CHH sites across the A, B and D sub-genomes of wheat. The *x*-axis is divided into 10 individual bins that correspond to methylation levels. The *y*-axis is the percentage of total counts for each respective bin. Here sequencing reads are only utilized if a SNP allele can assign them to a single genome only and at a minimum depth of 5X.

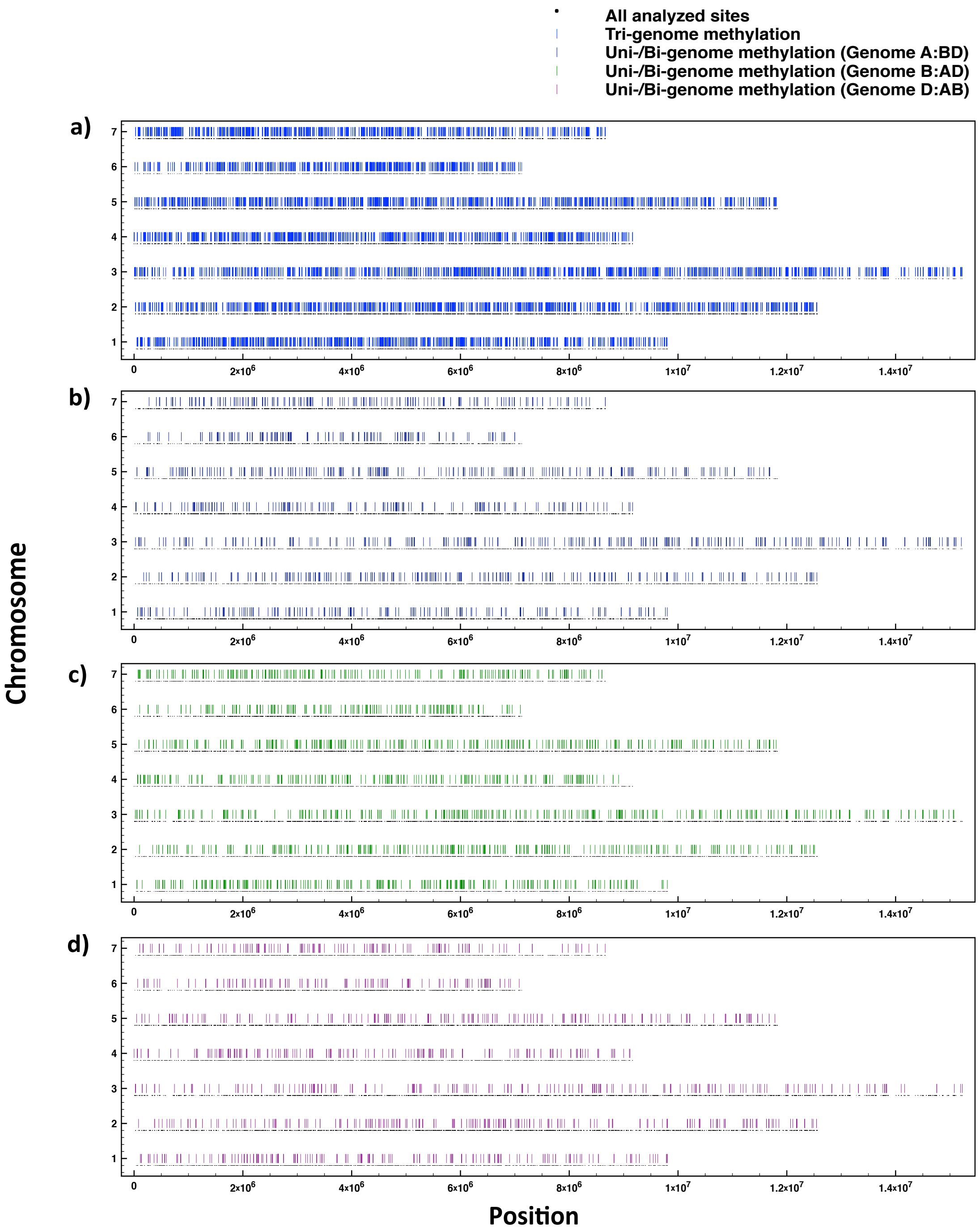

Figure 8. Positional information for methylation sites. Incidences of **a)** tri-genome methylation and uni-/bi-genome methylation in **b)** genome A/BD, **c)** genome B/AD and **d)** genome D/AB in the 27°C sample are detailed relative to all analyzed sites (methylated and non-methylated). Data is shown along each of the pseudo-chromosomes and methylation is classified using threshold values plus a 50% difference and q<0.01 for sites showing differential methylation between the sub-genomes.

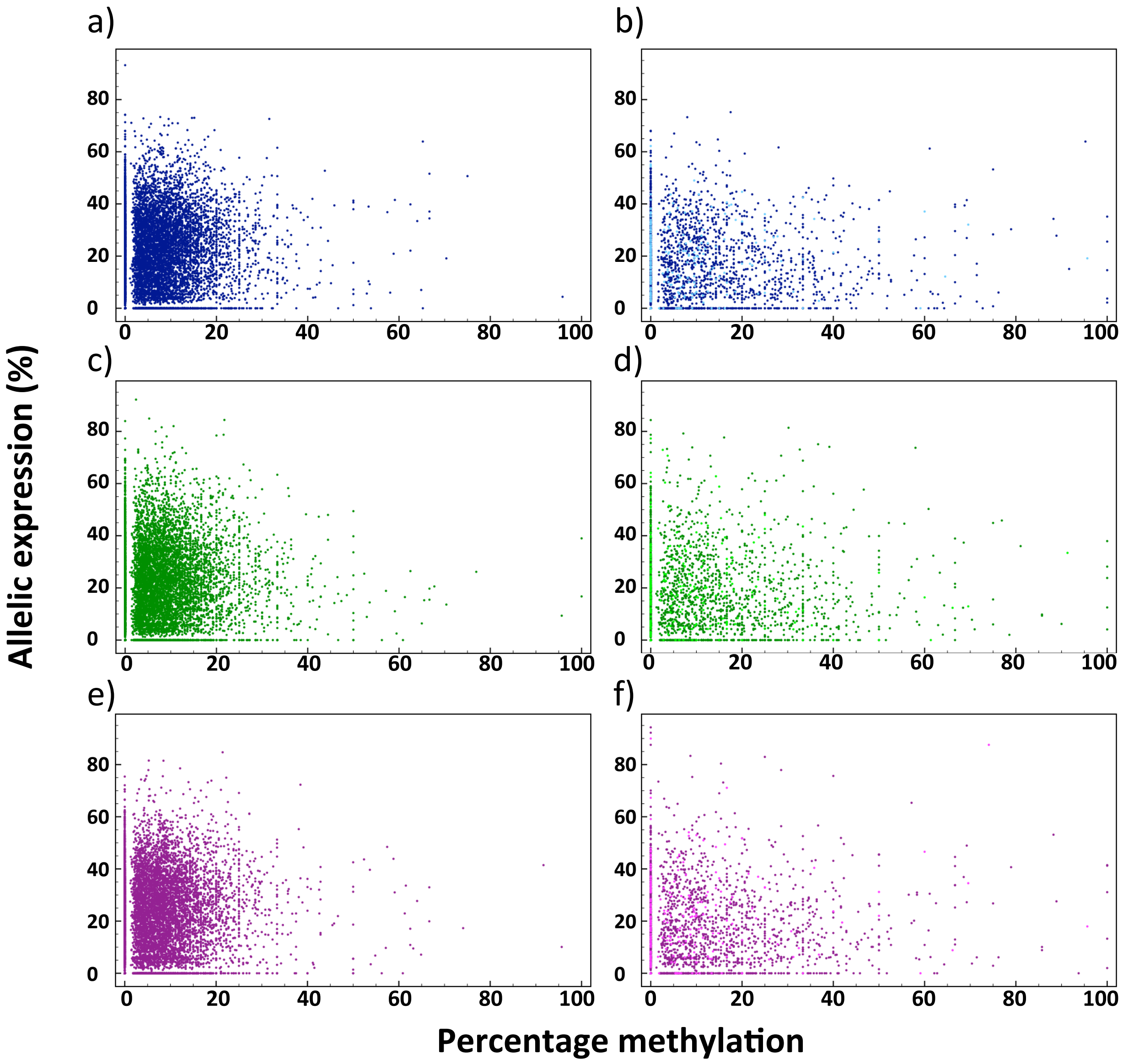

Figure 9. Association of methylated regions and gene expression. Percentage methylation per extended bait probe sequence versus percentage allelic gene expression for that sequence summarized for: a) b) sub-genome A, c) d) sub-genome B and e) f) sub-genome D. Data segregated according to transcription status: a) c) e) transcribed intron and exon regions and b) d) f) promoter regions (light colour) and remaining non-transcribed regions (dark colour). Percentage methylation per region (after division into exon/intron versus non-transcribed/promoter) calculated as the number of methylated cytosine residues (using standard thresholds) as a proportion of all cytosine residues in that gene region.

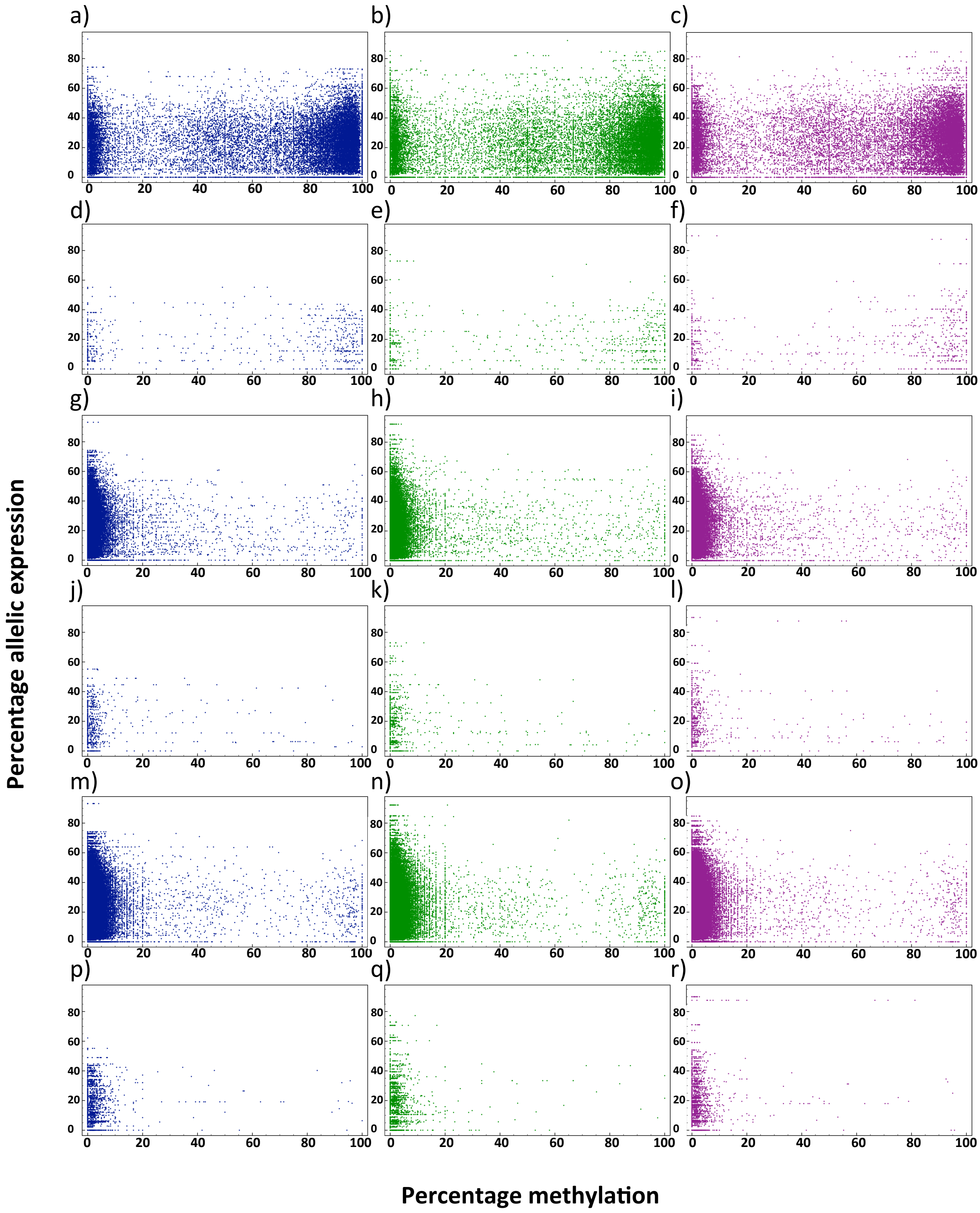

Figure 10. Association of single point methylation and gene expression. Percentage methylation at a single cytosine position versus percentage allelic gene expression for the extended bait sequence that the site originated from summarized for: a) d) g) j) m) p) sub-genome A, b) e) h) k) n) q) sub-genome B and c) f) i) l) o) r) sub-genome D. Data segregated according to transcription status: a) b) c) g) h) i) m) n) o) transcribed intron and exon regions and d) e) f) j) k) l) p) q) r) promoter regions. Data was further segregated by cytosine context: a-f) CpG context, g-l) CHG context and m-r) CHH context.

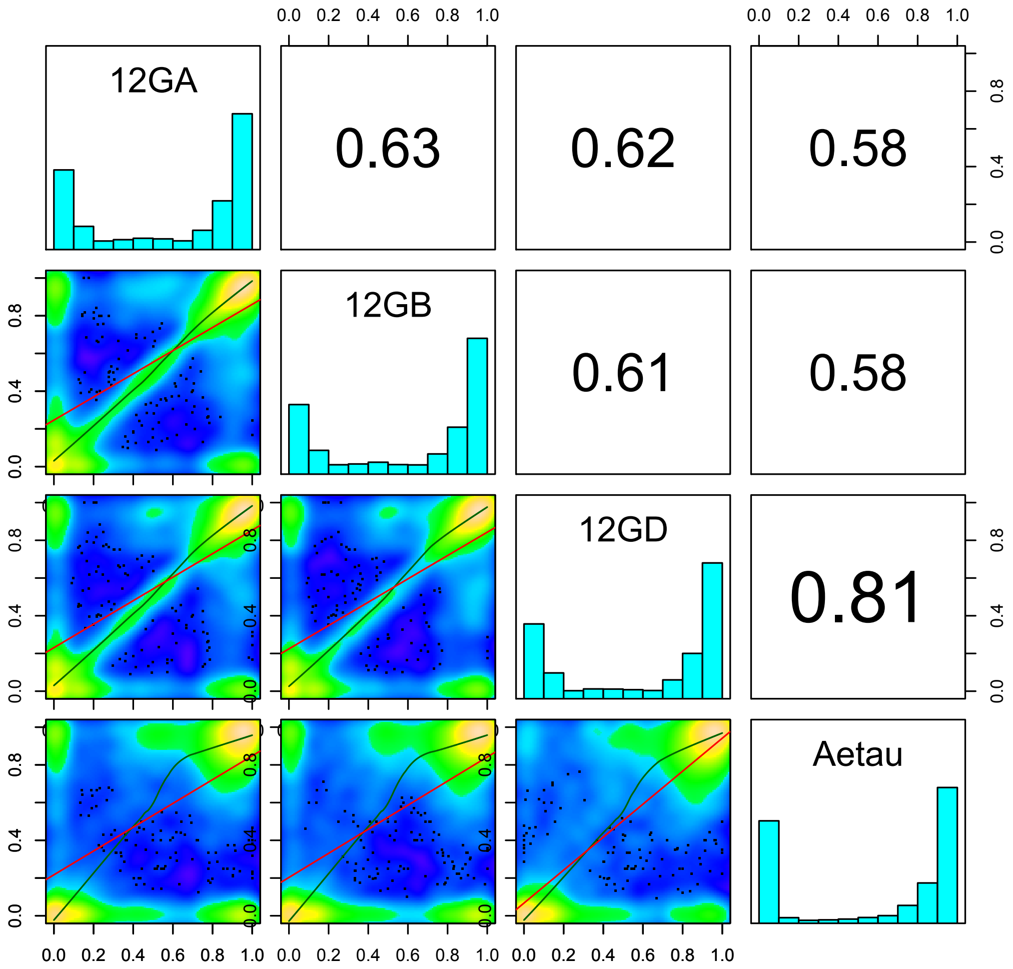

Figure 11. MethylKit[2] Pearson correlation coefficient computations to compare methylation between the sub-genome A, B and D of the 12°C sample and the D sub-genome progenitor *Ae. tauschii*. Figures demonstrate comparisons of methylation levels across the respective samples at positions that have information that is associated with the A sub-genome (12GA), the B sub-genome (12GB) and the D sub genome (12GD) of the 12°C sample and *Ae. Tauschii* (Aetau). Plots are opposite their respective correlation coefficients using a labeled horizontal axis and the intersection of two labels denotes their associated plots. Only cytosine positions where methylation been observed in 1 or more sub-genome of wheat and/or in *Ae. Tauschii* are compared. Where bi-allelic homoeologous SNPs have been used to assign methylation levels, information associated with two genomes will be shown in both plots.

Sample	Mean % coverage per 120-Mer probe	Mean depth of coverage per 120-Mer probe	Number of Probes mapped (50000 total)	% of reference 120-Mer probes mapped	Number of SNPs identified* (44Mb reference)
12B	97.2	89.8	49838	99	2,720,229
12C	96.9	70.4	49692	99	2,503835
12D	97.9	138.5	49928	99	3,381128
27B	97.1	80.9	49798	99	2,758078
27C	97.2	96.3	49807	99	2,836,307
27D	97.9	135.8	49917	99	3,320,338

Table 1. Mapping Statistics for six enriched and bisulfite treated wheat DNA samples. All mapping statistics in relation to the 6Mb array bait probe base space unless otherwise stated (*high numbers of SNP calls are likely for bisulfite treated samples due to un-methylated C→T conversion)

Table 2a

Methylation site Primer set 15 (668-800bp) CONTIG174481 _1-2021-596	Status in next generation sequencing data Sample 12	Peak in Sanger sequencing data Sample 12	Status in next generation sequencing data Sample 27	Status in Sanger sequencing data Sample 27
689	96.8% un-methylated	100% T (un-methylated)	97.6% un-methylated	100% T (un-methylated)
690	98.7% un-methylated	100% T (un-methylated)	99.6% un-methylated	100% T (un-methylated)
692	99.3% un-methylated	100% T (un-methylated)	98.5% un-methylated	100% T (un-methylated)
696	98.6% un-methylated	100% T (un-methylated)	98.6% un-methylated	100% T (un-methylated)
699	98.3% un-methylated	100% T (un-methylated)	98.2% un-methylated	100% T (un-methylated)
700	98.9% un-methylated	100% T (un-methylated)	99.5% un-methylated	100% T (un-methylated)
701	99.7% un-methylated	100% T (un-methylated)	99.9% un-methylated	100% T (un-methylated)
706	63.9% G/36.1% A (meth on F strand)	~50% A/50% G	64.4% G/35.6% A (meth on F strand)	~50% A/50% G
728	40% un-methylated	~60% C/40% T (un-methylated)	40% un-methylated	~60% C/40% T (un-methylated)
730	97.4% un-methylated	100% T (un-methylated)	99.0% un-methylated	100% T (un-methylated)
733	44.2% un-methylated	~60% C/40% T (un-methylated)	44.2% un-methylated	~60% C/40% T (un-methylated)
760	96.9% un-methylated	100% T (un-methylated)	98.1% un-methylated	100% T (un-methylated)
767	47.4% un-methylated	~55% C/45% T (un-methylated)	46.8% un-methylated	~55% C/45% T (un-methylated)
788	98.8% un-methylated	100% T (un-methylated)	98.9% un-methylated	100% T (un-methylated)
795	98.5% un-methylated	100% T (un-methylated)	97.9% un-methylated	100% T (un-methylated)

Table 2b

Methylation site Primer set 1 (951-1190bp) Contig318298_1-1640-953	Status in next generation sequencing data Sample 12	Peak in Sanger sequencing data Sample 12	Status in next generation sequencing data Sample 27	Status in Sanger sequencing data Sample 27
975	98.3% un-methylated	T (un-methylated)	99.1% un-methylated	T (un-methylated)
980	98% un-methylated	T (un-methylated)	96.9% un-methylated	T (un-methylated)
983	61% methylated	C (methylated)	63% methylated	C (methylated)
1000	99.2% un-methylated	T (un-methylated)	99.7%un-methylated	T (un-methylated)
1003	99% un-methylated	T (un-methylated)	99% un-methylated	T (un-methylated)
1006	99% un-methylated	T (un-methylated)	99% un-methylated	T (un-methylated)
1007	98% un-methylated	T (un-methylated)	98.6% un-methylated	T (un-methylated)
1009	99.5% un-methylated	T (un-methylated)	97.9% un-methylated	T (un-methylated)
1013	98.6% un-methylated	T (un-methylated)	99.1% un-methylated	T (un-methylated)
1033	99.2% un-methylated	T (un-methylated)	98.8% un-methylated	T (un-methylated)
1034	99.5% un-methylated	T (un-methylated)	99.1% un-methylated	T (un-methylated)
1041	99.5% un-methylated	T (un-methylated)	98.7% un-methylated	T (un-methylated)
1046	99.4% un-methylated	T (un-methylated)	98.1% un-methylated	T (un-methylated)
1053	98.1% un-methylated	T (un-methylated)	98.% un-methylated	T (un-methylated)
1080	98.4% un-methylated	T (un-methylated)	98.6% un-methylated	T (un-methylated)
1081	99.2% un-methylated	T (un-methylated)	98.8% un-methylated	T (un-methylated)
1086	99.2% un-methylated	T (un-methylated)	98.8% un-methylated	T (un-methylated)
1089	98.2% un-methylated	T (un-methylated)	98.3% un-methylated	T (un-methylated)
1090	99% un-methylated	T (un-methylated)	99.2% un-methylated	T (un-methylated)
1092	98.9% un-methylated	T (un-methylated)	98.3% un-methylated	T (un-methylated)
1093	99.3% un-methylated	T (un-methylated)	98.9% un-methylated	T (un-methylated)
1096	99.3% un-methylated	T (un-methylated)	99.5% un-methylated	T (un-methylated)
1103	96.9% un-methylated	T (un-methylated)	96.6% un-methylated	T (un-methylated)
1104	98.9% un-methylated	T (un-methylated)	98.6% un-methylated	T (un-methylated)
1107	100% un-methylated	T (un-methylated)	98.4% un-methylated	T (un-methylated)
1115	98.8 % un-methylated	T (un-methylated)	98.5% un-methylated	T (un-methylated)
1119	98.7% un-methylated	T (un-methylated)	99.6% un-methylated	T (un-methylated)
1125	96.6% un-methylated	T (un-methylated)	98.1% un-methylated	T (un-methylated)
1129	96.5% methylated	C (methylated)	96.5% methylated	C (methylated)
1138	98.5% methylated	C (methylated)	98.7% methylated	C (methylated)
1145	84% un-methylated	~20% C /~80% T	89% un-methylated	~20% C /~80% T
1146	99% un-methylated	T (un-methylated)	99.1% un-methylated	T(un-methylated)
1154	96.9% un-methylated	T (un-methylated)	95.9% un-methylated	T(un-methylated)

Table 2c

Methylation site<br>Primer set 15<br>(678-761bp)<br>CONTIG643204_1-1170-857	Status in next generation sequencing data<br>Sample 12	Peak in Sanger sequencing data Sample 12	Status in next generation sequencing data<br>Sample 27	Status in Sanger sequencing data<br>Sample 27
700	100% un-methylated	T (un-methylated)	100% un-methylated	T (un-methylated)
706	88.9% un-methylated	T (un-methylated)	100% un-methylated	T (un-methylated)
711	88.9% un-methylated	T (un-methylated)	100% un-methylated	T (un-methylated)
712	88.9% un-methylated	T (un-methylated)	95.5% un-methylated	T (un-methylated)
717	100% methylated	C (methylated)	84.6% methylated	C (methylated)
718	83.3% un-methylated	T (un-methylated)	92.0% un-methylated	T (un-methylated)
720	91.7% un-methylated	T (un-methylated)	92.3% un-methylated	T (un-methylated)
728	94.4% methylated	C (methylated)	85.7% methylated	C (methylated)
729	95.0% un-methylated	T (un-methylated)	97.3% un-methylated	T (un-methylated)
736	96.2% un-methylated	T (un-methylated)	95.2% un-methylated	T (un-methylated)
737	96.2% un-methylated	T (un-methylated)	97.6% un-methylated	T (un-methylated)
742	97.7% un-methylated	T (un-methylated)	98.0% un-methylated	T (un-methylated)
753	100% un-methylated	T (un-methylated)	98.5% un-methylated	T (un-methylated)
757	96.8% un-methylated	T (un-methylated)	98.6% un-methylated	T (un-methylated)

Table 2d

**Methylation site Primer set 2 (1374-1684bp) Contig44098_1040-2994-1412**	**Status in next generation sequencing data Sample 12**	**Peak in Sanger sequencing data Sample 12**	**Status in next generation sequencing data Sample 27**	**Status in Sanger sequencing data Sample 27**
1404	98.8% un-methylated	T (un-methylated)	99.1% un-methylated	T (un-methylated)
1407	97.9% un-methylated	T (un-methylated)	98.9% un-methylated	T (un-methylated)
1412	98.9% un-methylated	T (un-methylated)	98.1% un-methylated	T (un-methylated)
1413	100% methylated (G as F seq C)	G (methylated)	100% methylated	G (methylated)
1414	95.9% methylated	C (methylated)	88.5% methylated	C (methylated)
1417	92.9% methylated	~60% C (methylated) 40% G	87.9% methylated	~50% C (methylated) 50% G
1424	98.3% un-methylated	T (un-methylated)	99.1% un-methylated	T (un-methylated)
1426	99.8% un-methylated	T (un-methylated)	99.0% un-methylated	T (un-methylated)
1435	99.3% un-methylated	T (un-methylated)	99.8% un-methylated	T (un-methylated)
1445	98.7% un-methylated	T (un-methylated)	99.4% un-methylated	T (un-methylated)
1448	99.7% un-methylated	T (un-methylated)	98.4% un-methylated	T (un-methylated)
1459	99.9% un-methylated	T (un-methylated)	98.9% un-methylated	T (un-methylated)
1469	98.8% un-methylated	T (un-methylated)	98.2% un-methylated	T (un-methylated)
1470	98.6% un-methylated	T (un-methylated)	99.5% un-methylated	T (un-methylated)
1472	99.5% un-methylated	T (un-methylated)	99.0% un-methylated	T (un-methylated)
1473	98.0% un-methylated	T (un-methylated)	99.2% un-methylated	T (un-methylated)
1474	99.6% un-methylated	T (un-methylated)	99.2% un-methylated	T (un-methylated)
1476	85.5% methylated	C ~90%/T ~10%	84.4% methylated	C ~90%/T ~10%
1479	98.8% un-methylated	T (un-methylated)	98.9% un-methylated	T (un-methylated)
1480	99.3% un-methylated	T (un-methylated)	99.4% un-methylated	T (un-methylated)
1485	98.9% un-methylated	T (un-methylated)	98.9% un-methylated	T (un-methylated)
1489	99.3% un-methylated	T (un-methylated)	99.5% un-methylated	T (un-methylated)
1491	74.9% un-methylated	C ~1/3%/ T ~ 2/3%	70.2% un-methylated	C ~40% /T ~60%
1498	99.0% un-methylated	T (un-methylated)	99.0% un-methylated	T (un-methylated)
1503	99.4% un-methylated	T (un-methylated)	99.3% un-methylated	T (un-methylated)
1514	98.5% un-methylated	T (un-methylated)	98.5% un-methylated	T (un-methylated)
1515	99.1% un-methylated	T (un-methylated)	100% un-methylated	T (un-methylated)
1521	100% un-methylated	T (un-methylated)	99.4% un-methylated	T (un-methylated)
1528	56.5% methylated	C ~ 50%/T ~ 50%	59.0% methylated	C ~ 50%/T ~ 50%
1533	44.9% methylated	C ~ 50%/T ~ 50%	43.4% methylated	C ~ 50%/T ~ 50%
1535	99.4% un-methylated	T (un-methylated)	97.6% un-methylated	T (un-methylated)
1543	88.0% methylated	~90% C/ ~10% T	87.9% methylated	~90% C /~10% T
1544	99.3% un-methylated	T (un-methylated)	99.7% un-methylated	T(un-methylated)
1551	100% un-methylated	T (un-methylated)	99.6% un-methylated	T(un-methylated)
1555	92.9% un-methylated	T (un-methylated)/ ~10% C	90.6% un-methylated	T (un-methylated)/ ~10% C
1557	99.4% un-methylated	T (un-methylated)	99.5% un-methylated	T (un-methylated)
1558	99.4% un-methylated	T (un-methylated)	100% un-methylated	T (un-methylated)
1567	100% un-methylated	T (un-methylated)	99.2% un-methylated	T (un-methylated)
1574	95.9% un-methylated	T (un-methylated)	96.3% un-methylated	T (un-methylated)
1576	98.5% un-methylated	T (un-methylated)	97.3% un-methylated	T (un-methylated)

Table 2e

Methylation site<br>Primer set 3<br>(1387-1526bp)<br>CONTIG248041_1-<br>1793-1442	Status in next generation sequencing data<br>Sample 12	Peak in Sanger sequencing data<br>Sample 12	Status in next generation<br>sequencing data<br>Sample 27	Status in Sanger<br>sequencing data<br>Sample 27
1387	100% un-methylated	T (un-methylated)	100% un-methylated	T (un-methylated)
1389	98.6% un-methylated	T (un-methylated)	100% un-methylated	T (un-methylated)
1390	98.7% un-methylated	T (un-methylated)	97% un-methylated	T (un-methylated)
1391	98.8% un-methylated	T (un-methylated)	97% un-methylated	T (un-methylated)
1392	100% un-methylated	T (un-methylated)	93.9% un-methylated	T (un-methylated)
1395	94.7% un-methylated	T (un-methylated)	97% un-methylated	T (un-methylated)
1396	100% un-methylated	T (un-methylated)	93.9% un-methylated	T (un-methylated)
1399	99.0% un-methylated	T (un-methylated)	97% un-methylated	T (un-methylated)
1400	100% un-methylated	T (un-methylated)	94.1% un-methylated	T (un-methylated)
1403	100% un-methylated	T (un-methylated)	96.4% un-methylated	T (un-methylated)
1409	100% un-methylated	T (un-methylated)	96.9% un-methylated	T (un-methylated)
1421	96.2% un-methylated	T (un-methylated)	97.7% un-methylated	T (un-methylated)
1423	99.3% un-methylated	T (un-methylated)	98.9% un-methylated	T (un-methylated)
1428	100% un-methylated	T (un-methylated)	97.8% un-methylated	T (un-methylated)
1430	98.6% un-methylated	T (un-methylated)	97.9% un-methylated	T (un-methylated)
1436	99.4% un-methylated	T (un-methylated)	97.4% un-methylated	T (un-methylated)
1438	98.8% un-methylated	T (un-methylated)	99.1% un-methylated	T (un-methylated)
1440	99.4% un-methylated	T (un-methylated)	96.6% un-methylated	T (un-methylated)
1442	100% un-methylated	T (un-methylated)	94.6% un-methylated	T (un-methylated)
1444	97.8% un-methylated	T (un-methylated)	97.9% un-methylated	T (un-methylated)
1447	99.5% un-methylated	T (un-methylated)	99.4% un-methylated	T (un-methylated)
1454	98.5% un-methylated	T (un-methylated)	98.4% un-methylated	T (un-methylated)
1456	99.1% un-methylated	T (un-methylated)	98.9% un-methylated	T (un-methylated)
1461	98.2% un-methylated	T (un-methylated)	99.5% un-methylated	T (un-methylated)
1469	98.8% un-methylated	T (un-methylated)	98.5% un-methylated	T (un-methylated)
1471	98.8% un-methylated	T (un-methylated)	99.5% un-methylated	T (un-methylated)
1472	99.2% un-methylated	T (un-methylated)	98.5% un-methylated	T (un-methylated)
1473	99.2% un-methylated	T (un-methylated)	99.0% un-methylated	T (un-methylated)
1475	95.8% un-methylated	T (un-methylated)	97.0% un-methylated	T (un-methylated)
1476	98.9% un-methylated	T (un-methylated)	98.5% un-methylated	T (un-methylated)
1478	98.5% un-methylated	T (un-methylated)	97.6% un-methylated	T (un-methylated)
1482	99.6% un-methylated	T (un-methylated)	98.6% un-methylated	T (un-methylated)
1490	99.6% un-methylated	T (un-methylated)	99.1% un-methylated	T (un-methylated)
1496	99.3% un-methylated	T (un-methylated)	98.8% un-methylated	T (un-methylated)
1498	98.5% un-methylated	T (un-methylated)	97.9% un-methylated	T (un-methylated)
1503	99.3% un-methylated	T (un-methylated)	99.2% un-methylated	T (un-methylated)
1510	99.2% un-methylated	T (un-methylated)	99.1% un-methylated	T (un-methylated)
1512	99.6% un-methylated	T (un-methylated)	97.8% un-methylated	T (un-methylated)
1515	98.8% un-methylated	T (un-methylated)	98.6% un-methylated	T (un-methylated)
1516	99.2% un-methylated	T (un-methylated)	98.2% un-methylated	T (un-methylated)
1517	99.6% un-methylated	T (un-methylated)	97.3% un-methylated	T (un-methylated)
1521	99.6% un-methylated	T (un-methylated)	98.3% un-methylated	T (un-methylated)
1526	100% un-methylated	T (un-methylated)	99.6% un-methylated	T (un-methylated)

Table 2f

Methylation site Primer set 15 (79-423bp) CONTIG256716 _1-1768-36	Status in next generation sequencing data Sample 12	Peak in Sanger sequencing data Sample 12	Status in next generation sequencing data Sample 27	Status in Sanger sequencing data Sample 27
102	28.8% un-methylated (C on F)	~20% A (un-methylated)/~80% G	28.6% un-methylated (C on F)	~20% A (un-methylated)/~80% G
106	98.8% un-methylated	T (un-methylated)	98.7% un-methylated	T (un-methylated)
115	98.5% un-methylated	T (un-methylated)	98.8% un-methylated	T (un-methylated)
118	98.5% un-methylated	T (un-methylated)	99.6% un-methylated	T (un-methylated)
127	99% un-methylated	T (un-methylated)	98.8% un-methylated	T (un-methylated)
131	98.3% un-methylated	T (un-methylated)	97.6% un-methylated	T (un-methylated)
136	98.5% un-methylated	T (un-methylated)	98.5% un-methylated	T (un-methylated)
140	99.3% un-methylated	T (un-methylated)	99.3% un-methylated	T (un-methylated)
144	97.5% un-methylated	T (un-methylated)	97.5% un-methylated	T (un-methylated)
145	98.0% un-methylated	T (un-methylated)	98.5% un-methylated	T (un-methylated)
149	99.2% un-methylated	T (un-methylated)	98.2% un-methylated	T (un-methylated)
152	98.4% un-methylated	T (un-methylated)	97.7% un-methylated	T (un-methylated)
156	30.2% methylated (C on F)	~ 60% A (un-methylated)/ ~40% G	32.2% methylated (C on F)	~ 60% A (un-methylated)/ ~40% G
159	98.1% un-methylated	T (un-methylated)	98.5% un-methylated	T (un-methylated)
161	98.4% un-methylated	T (un-methylated)	98.0% un-methylated	T (un-methylated)
163	97.1% un-methylated	T (un-methylated)	97.1% un-methylated	T (un-methylated)
164	98.4% un-methylated	T (un-methylated)	98.2% un-methylated	T (un-methylated)
165	98.9% un-methylated	T (un-methylated)	98.5% un-methylated	T (un-methylated)
166	98.9% un-methylated	T (un-methylated)	98.8% un-methylated	T (un-methylated)
172	98.9% un-methylated	T (un-methylated)	98.7% un-methylated	T (un-methylated)
186	96.6% un-methylated	T (un-methylated)	98.1% un-methylated	T (un-methylated)
187	98.4% un-methylated	T (un-methylated)	98.4% un-methylated	T (un-methylated)
188	97.5% un-methylated	T (un-methylated)	99.0% un-methylated	T (un-methylated)
197	97.1% un-methylated	T (un-methylated)	97.7% un-methylated	T (un-methylated)
199	97.4% un-methylated	T (un-methylated)	97.6% un-methylated	T (un-methylated)
201	95.9% un-methylated	T (un-methylated)	96.1% un-methylated	T (un-methylated)
202	98.6% un-methylated	T (un-methylated)	99.0% un-methylated	T (un-methylated)
220	96% un-methylated	T (un-methylated)	97.4% un-methylated	T (un-methylated)
229	97.7% un-methylated	T (un-methylated)	98.1% un-methylated	T (un-methylated)
232	97.5% un-methylated	T (un-methylated)	97.0% un-methylated	T (un-methylated)
233	96.8% un-methylated	T (un-methylated)	98.0% un-methylated	T (un-methylated)
237	97.4% un-methylated	T (un-methylated)	96.9% un-methylated	T (un-methylated)
239	97.4% un-methylated	T (un-methylated)	99.5% un-methylated	T (un-methylated)
255	10.1% un-methylated	~10% un-methylated	8.7% un-methylated	~10% un-methylated
274	96.6% un-methylated	T (un-methylated)	92.8% un-methylated	T (un-methylated)
275	96.6% un-methylated	T (un-methylated)	98.8% un-methylated	T (un-methylated)
279	96.6% un-methylated	T (un-methylated)	98.8% un-methylated	T (un-methylated)
283	98.1% un-methylated	T (un-methylated)	98.7% un-methylated	T (un-methylated)
286	98.0% un-methylated	T (un-methylated)	97.3% un-methylated	T (un-methylated)
308	100% un-methylated	T (un-methylated)	97.1% un-methylated	T (un-methylated)
310	100% un-methylated	T (un-methylated)	90.9% un-methylated	T (un-methylated)
311	100% un-methylated	T (un-methylated)	100% un-methylated	T (un-methylated)
316	95.8% un-methylated	T (un-methylated)	100% un-methylated	T (un-methylated)

Table 2g

Methylation site Primer set 15 (893-1115bp) CONTIG462845_1 -1429-894	Status in next generation sequencing data Sample 12	Peak in Sanger sequencing data Sample 12	Methylation site	Status in next generation sequencing data Sample 12	Status in Sanger sequencing data Sample 12
894	98.4% un-methylated	T (un-methylated)	964	98.9% un-methylated	T (un-methylated)
899	100% un-methylated	T (un-methylated)	965	98.9% un-methylated	T (un-methylated)
900	99.7% un-methylated	T (un-methylated)	972	99.2% un-methylated	T (un-methylated)
901	99.3% un-methylated	T (un-methylated)	981	99.5% un-methylated	T (un-methylated)
906	99.7% un-methylated	T (un-methylated)	983	98.2% un-methylated	T (un-methylated)
907	99.3% un-methylated	T (un-methylated)	984	100% un-methylated	T (un-methylated)
912	97.3% un-methylated	T (un-methylated)	985	98.4% un-methylated	T (un-methylated)
924	98.7% un-methylated	T (un-methylated)	987	96.1% un-methylated	T (un-methylated)
926	99.4% un-methylated	T (un-methylated)	990	99.5% un-methylated	T (un-methylated)
928	98.7% un-methylated	T (un-methylated)	992	99.0% un-methylated	T (un-methylated)
930	56.6% un-methylated	~60% T (un-methylated)/ ~40% C	996	99.2% un-methylated	T (un-methylated)
931	99.4% un-methylated	T (un-methylated)	1003	98.3% un-methylated	T (un-methylated)
937	98.7% un-methylated	T (un-methylated)	1015	98.0% un-methylated	T (un-methylated)
940	95.7% un-methylated	T (un-methylated)	1017	98.4% un-methylated	T (un-methylated)
941	98.1% un-methylated	T (un-methylated)	1019	98.8% un-methylated	T (un-methylated)
942	99.1% un-methylated	T (un-methylated)	1024	98.4% un-methylated	T (un-methylated)
946	97.8% un-methylated	T (un-methylated)	1026	99.2% un-methylated	T (un-methylated)
949	99.8% un-methylated	T (un-methylated)	1036	98.1% un-methylated	T (un-methylated)
958	99.3% un-methylated	T (un-methylated)	1039	98.5% un-methylated	T (un-methylated)
960	97.8% un-methylated	T (un-methylated)	1048	98.2% un-methylated	T (un-methylated)
962	99.3% un-methylated	T (un-methylated)			

Table 2h

Methylation site Primer set 15 (1145-1290bp) CONTIG381103 _1-1538-1308	Status in next generation sequencing data Sample 12	Peak in Sanger sequencing data Sample 12	Status in next generation sequencing data Sample 27	Status in Sanger sequencing data Sample 27
1145	100% un-methylated	T (un-methylated)	100% un-methylated	T (un-methylated)
1147	100% un-methylated	T (un-methylated)	100% un-methylated	T (un-methylated)
1150	100% un-methylated	T (un-methylated)	100% un-methylated	T (un-methylated)
1153	100% un-methylated	T (un-methylated)	100% un-methylated	T (un-methylated)
1155	100% un-methylated	T (un-methylated)	100% un-methylated	T (un-methylated)
1162	100% un-methylated	T (un-methylated)	100% un-methylated	T (un-methylated)
1164	78.6% methylated	C (methylated)	84.6% methylated	C (methylated)
1167	86.7% un-methylated	~50% C/50% T	83.3% un-methylated	~50% C/50% T
1170	100% un-methylated	T (un-methylated)	100% un-methylated	T (un-methylated)
1171	100% un-methylated	T (un-methylated)	100% un-methylated	T (un-methylated)
1175	95.2% un-methylated	T (un-methylated)	100% un-methylated	T (un-methylated)
1176	100% un-methylated	T (un-methylated)	100% un-methylated	T (un-methylated)
1182	100% un-methylated	T (un-methylated)	100% un-methylated	T (un-methylated)
1193	100% un-methylated	T (un-methylated)	97.7% un-methylated	T (un-methylated)
1194	96.4% un-methylated	T (un-methylated)	97.7% un-methylated	T (un-methylated)
1199	97.1% un-methylated	T (un-methylated)	96.5% un-methylated	T (un-methylated)
1200	100% un-methylated	T (un-methylated)	100% un-methylated	T (un-methylated)
1201	100% un-methylated	T (un-methylated)	98.2% un-methylated	T (un-methylated)
1202	100% un-methylated	T (un-methylated)	100% un-methylated	T (un-methylated)
1205	100% un-methylated	T (un-methylated)	100% un-methylated	T (un-methylated)
1212	100% un-methylated	T (un-methylated)	100% un-methylated	T (un-methylated)
1218	100% un-methylated	T (un-methylated)	100% un-methylated	T (un-methylated)
1229	100% un-methylated	T (un-methylated)	98.6% un-methylated	T (un-methylated)
1240	99.2% un-methylated	T (un-methylated)	100% un-methylated	T (un-methylated)
1248	41.0% un-methylated	~40% C/ ~60% T (un-methylated)	44.4% un-methylated	~40% C/ ~60% T (un-methylated)
1253	97.0% un-methylated	T (un-methylated)	97.8% un-methylated	T (un-methylated)
1275	100% un-methylated	T (un-methylated)	100% un-methylated	T (un-methylated)
1277	98.8% un-methylated	T (un-methylated)	98.1% un-methylated	T (un-methylated)
1278	100% un-methylated	T (un-methylated)	100% un-methylated	T (un-methylated)
1283	99.4% un-methylated	T (un-methylated)	100% un-methylated	T (un-methylated)
1288	98.8% un-methylated	T (un-methylated)	100% un-methylated	T (un-methylated)
1290	99.4% un-methylated	T (un-methylated)	100% un-methylated	T (un-methylated)

Table 2i

Methylation site Primer set 15 (217-503bp) CONTIG117347 4_1-941-195	Status in next generation sequencing data Sample 12	Peak in Sanger sequencing data Sample 12	Status in next generation sequencing data Sample 27	Status in Sanger sequencing data Sample 27
252	71.8% un-methylated	T (un-methylated)	89.1% un-methylated	T (un-methylated)
271	100% un-methylated	T (un-methylated)	97.1% un-methylated	T (un-methylated)
275	27.7% methylated (C on F)	A (un-methylated)	29.1% methylated (C on F)	A (un-methylated)
278	98.2% un-methylated	T (un-methylated)	98.3% un-methylated	T (un-methylated)
287	94.9% un-methylated	T (un-methylated)	100% un-methylated	T (un-methylated)
299	97% methylated	C (methylated)	96.3% methylated	C (methylated)
309	80.9% un-methylated	~80% T (un-methylated)/~20% C	79.9% un-methylated	~70% T (un-methylated)/~30% C
323	99.3% un-methylated	T (un-methylated)	99.5% un-methylated	T (un-methylated)
339	98.9% un-methylated	T (un-methylated)	100% un-methylated	T (un-methylated)
342	94.4% un-methylated	T (un-methylated)	94.1% un-methylated	T (un-methylated)
345	94.4% un-methylated	T (un-methylated)	97.9% un-methylated	T (un-methylated)
346	97.2% un-methylated	T (un-methylated)	100% un-methylated	T (un-methylated)
351	89.8% un-methylated	~90% T (un-methylated)/~10% C	90.9% un-methylated	~90% T (un-methylated)/~10% C
359	97.8% un-methylated	T (un-methylated)	97.0% un-methylated	T (un-methylated)
364	98.2% un-methylated	T (un-methylated)	98.6% un-methylated	T (un-methylated)
368	98.3% methylated	C (methylated)	96.7% methylated	C(methylated)
370	98.1% un-methylated	T (un-methylated)	97.5% un-methylated	T (un-methylated)
374	97.8% un-methylated	T (un-methylated)	98.9% un-methylated	T (un-methylated)
376	97.0% un-methylated	T (un-methylated)	100% un-methylated	T (un-methylated)
381	94.4% un-methylated	T (un-methylated)	96.9% un-methylated	T (un-methylated)
388	98.0% un-methylated	T (un-methylated)	100% un-methylated	T (un-methylated)
390	99.0% un-methylated	T (un-methylated)	98.2% un-methylated	T (un-methylated)
393	94.1% un-methylated	T (un-methylated)	100% un-methylated	T (un-methylated)
403	95.9% un-methylated	T (un-methylated)	97.3% un-methylated	T (un-methylated)
409	83.3% un-methylated	~80% T (un-methylated)/ ~20% C	78.3% un-methylated	~80% T (un-methylated)/ ~20% C
410	98.3% un-methylated	T (un-methylated)	98.3% un-methylated	T (un-methylated)
414	98.2% un-methylated	T (un-methylated)	98.1% un-methylated	T (un-methylated)
429	92.9% un-methylated	T (un-methylated)	100% un-methylated	T (un-methylated)
430	100% un-methylated	T (un-methylated)	100% un-methylated	T (un-methylated)
435	100% un-methylated	T (un-methylated)	100% un-methylated	T (un-methylated)

Table 2j

Methylation site<br>Primer set 15<br>(893-1115bp)<br>CONTIG462845<br>_1-1429-894	Status in next generation sequencing data<br>Sample 12	Peak in Sanger sequencing data Sample 12	Status in next generation sequencing data<br>Sample 27	Status in Sanger sequencing data<br>Sample 27
894	98.4% un-methylated	T (un-methylated)	98.3% un-methylated	T (un-methylated)
899	100% un-methylated	T (un-methylated)	98.6% un-methylated	T (un-methylated)
900	99.7% un-methylated	T (un-methylated)	98.2% un-methylated	T (un-methylated)
901	99.3% un-methylated	T (un-methylated)	98.2% un-methylated	T (un-methylated)
906	99.7% un-methylated	T (un-methylated)	97.9% un-methylated	T (un-methylated)
907	99.3% un-methylated	T (un-methylated)	97.5% un-methylated	T (un-methylated)
912	97.3% un-methylated	T (un-methylated)	99.4% un-methylated	T (un-methylated)
924	98.7% un-methylated	T (un-methylated)	98.8% un-methylated	T (un-methylated)
926	99.4% un-methylated	T (un-methylated)	98.1% un-methylated	T (un-methylated)
928	98.7% un-methylated	T (un-methylated)	99.8% un-methylated	T (un-methylated)
930	56.6% un-methylated	~60% T (un-methylated)/ ~40% C	51.3% un-methylated	~55% T (un-methylated)/ ~45% C
931	99.4% un-methylated	T (un-methylated)	99.8% un-methylated	T (un-methylated)
937	98.7% un-methylated	T (un-methylated)	99.1% un-methylated	T (un-methylated)
940	95.7% un-methylated	T (un-methylated)	95.4% un-methylated	T (un-methylated)
941	98.1% un-methylated	T (un-methylated)	99.5% un-methylated	T (un-methylated)
942	99.1% un-methylated	T (un-methylated)	99.3% un-methylated	T (un-methylated)
946	97.8% un-methylated	T (un-methylated)	99.4% un-methylated	T (un-methylated)
949	99.8% un-methylated	T (un-methylated)	99.1% un-methylated	T (un-methylated)
958	99.3% un-methylated	T (un-methylated)	99.8% un-methylated	T (un-methylated)
960	97.8% un-methylated	T (un-methylated)	98.6% un-methylated	T (un-methylated)
962	99.3% un-methylated	T (un-methylated)	98.8% un-methylated	T (un-methylated)
964	98.9% un-methylated	T (un-methylated)	98.6% un-methylated	T (un-methylated)
965	98.9% un-methylated	T (un-methylated)	100% un-methylated	T (un-methylated)
972	99.2% un-methylated	T (un-methylated)	99.1% un-methylated	T (un-methylated)
981	99.5% un-methylated	T (un-methylated)	99.7% un-methylated	T (un-methylated)
983	98.2% un-methylated	T (un-methylated)	97.8% un-methylated	T (un-methylated)
984	100% un-methylated	T (un-methylated)	100% un-methylated	T (un-methylated)
985	98.4% un-methylated	T (un-methylated)	98.6% un-methylated	T (un-methylated)
987	96.1% un-methylated	T (un-methylated)	98.4% un-methylated	T (un-methylated)
990	99.5% un-methylated	T (un-methylated)	99.2% un-methylated	T (un-methylated)
992	99.0% un-methylated	T (un-methylated)	98.2% un-methylated	T (un-methylated)
996	99.2% un-methylated	T (un-methylated)	98.9% un-methylated	T (un-methylated)
1003	98.3% un-methylated	T (un-methylated)	96.9% un-methylated	T (un-methylated)
1015	98.0% un-methylated	T (un-methylated)	98.4% un-methylated	T (un-methylated)
1017	98.4% un-methylated	T (un-methylated)	99.6% un-methylated	T (un-methylated)
1019	98.8% un-methylated	T (un-methylated)	98.8% un-methylated	T (un-methylated)
1024	98.4% un-methylated	T (un-methylated)	99.1% un-methylated	T (un-methylated)
1026	99.2% un-methylated	T (un-methylated)	96.6% un-methylated	T (un-methylated)
1036	98.1% un-methylated	T (un-methylated)	99.5% un-methylated	T (un-methylated)
1039	98.5% un-methylated	T (un-methylated)	99.0% un-methylated	T (un-methylated)
1048	98.2% un-methylated	T (un-methylated)	95.7% un-methylated	T (un-methylated)
1051	96.9% un-methylated	T (un-methylated)	98.7% un-methylated	T (un-methylated)
1053	98.1% un-methylated	T (un-methylated)	98.7% un-methylated	T (un-methylated)
1054	98.1% un-methylated	T (un-methylated)	99.3% un-methylated	T (un-methylated)
1063	97.7% un-methylated	T (un-methylated)	100% un-methylated	T (un-methylated)
1066	97.5% un-methylated	T (un-methylated)	100% un-methylated	T (un-methylated)
1070	96.0% un-methylated	T (un-methylated)	100% un-methylated	T (un-methylated)
1072	98.0% un-methylated	T (un-methylated)	100% un-methylated	T (un-methylated)
1078	92.5% un-methylated	T (un-methylated)	100% un-methylated	T (un-methylated)
1080	93.7% un-methylated	T (un-methylated)	100% un-methylated	T (un-methylated)
1082	91.7% un-methylated	T (un-methylated)	100% un-methylated	T (un-methylated)
1085	90.9% un-methylated	T (un-methylated)	100% un-methylated	T (un-methylated)
1086	93.9% un-methylated	T (un-methylated)	100% un-methylated	T (un-methylated)

Table 2k

Methylation site Primer set 15 (436-806bp) CONTIG796042 _275-1136-650	Status in next generation sequencing data Sample 12	Peak in Sanger sequencing data Sample 12	Status in next generation sequencing data Sample 27	Status in Sanger sequencing data Sample 27
545	100% un-methylated	T (un-methylated)	100% un-methylated	T (un-methylated)
573	100% un-methylated	T (un-methylated)	100% un-methylated	T (un-methylated)
581	100% un-methylated	T (un-methylated)	100% un-methylated	T (un-methylated)
582	100% un-methylated	T (un-methylated)	100% un-methylated	T (un-methylated)
584	63.0% un-methylated	~50% T (un-methylated)/50% C	56% un-methylated	~50% T (un-methylated)/50% C
594	100% un-methylated	T (un-methylated)	96.2% un-methylated	T (un-methylated)
601	96.3% un-methylated	T (un-methylated)	100% un-methylated	T (un-methylated)
603	50.0% un-methylated	~50% T (un-methylated)/50% C	53.9% un-methylated	~50% T (un-methylated)/50% C
614	100% un-methylated	T (un-methylated)	100% un-methylated	T (un-methylated)
619	100% un-methylated	T (un-methylated)	100% un-methylated	T (un-methylated)
621	100% un-methylated	T (un-methylated)	100% un-methylated	T (un-methylated)
627	100% un-methylated	T (un-methylated)	100% un-methylated	T (un-methylated)
630	100% un-methylated	T (un-methylated)	100% un-methylated	T (un-methylated)
632	100% un-methylated	T (un-methylated)	100% un-methylated	T (un-methylated)
635	100% un-methylated	T (un-methylated)	100% un-methylated	T (un-methylated)
639	100% un-methylated	T (un-methylated)	100% un-methylated	T (un-methylated)
641	100% un-methylated	T (un-methylated)	100% un-methylated	T (un-methylated)
646	100% un-methylated	T (un-methylated)	100% un-methylated	T (un-methylated)
655	100% un-methylated	T (un-methylated)	100% un-methylated	T (un-methylated)
661	100% un-methylated	T (un-methylated)	100% un-methylated	T (un-methylated)
670	100% un-methylated	T (un-methylated)	83.3% un-methylated	T (un-methylated)
677	100% un-methylated	T (un-methylated)	100% un-methylated	T (un-methylated)
680	100% un-methylated	T (un-methylated)	100% un-methylated	T (un-methylated)
687	100% un-methylated	T (un-methylated)	100% un-methylated	T (un-methylated)
696	98.3% un-methylated	T (un-methylated)	98.4% un-methylated	T (un-methylated)
698	96.7% un-methylated	T (un-methylated)	96.9% un-methylated	T (un-methylated)
699	98.3% un-methylated	T (un-methylated)	98.5% un-methylated	T (un-methylated)
705	96.5% un-methylated	T (un-methylated)	100% un-methylated	T (un-methylated)
710	97.7% un-methylated	T (un-methylated)	95.5% un-methylated	T (un-methylated)
712	98.9% un-methylated	T (un-methylated)	98.9% un-methylated	T (un-methylated)
716	94.6% un-methylated	T (un-methylated)	100% un-methylated	T (un-methylated)
719	99.0% un-methylated	T (un-methylated)	100% un-methylated	T (un-methylated)
735	100% un-methylated	T (un-methylated)	99.3% un-methylated	T (un-methylated)
741	97.9% un-methylated	T (un-methylated)	99.3% un-methylated	T (un-methylated)

Table 2a-k. Methylation site validation data. Observations from the Sanger sequencing output generated for the 12°C sample (supplementary figure 5). These methylation sites were originally identified in the next generation sequencing data that was derived from enriched bisulfite treated DNA and sites extend across 10 of the methylation array extended bait probe sequences. This data outlines the validation of these methylation sites at individual cytosine residues.

Type	Number of reads	Percentage of total (%)	Brenchley *et al.* Percentage of total (%)	Percentage of sites methylated (%)
**DNA transposons**	**1408443**	**23.669**	**18.691**	**25.184**
Helitron	2319	0.039	0.303	22.549
TIR	1403070	23.579	18.311	25.249
HAT	1034	0.017	0.052	23.323
Harbinger	73350	1.233	0.427	27.952
Mariner	140677	2.364	0.128	20.533
CACTA	1082075	18.185	15.995	25.455
Mutator	105743	1.777	0.557	18.413
Unknown	191	0.003	0.011	41.791
Unknown	3054	0.051	0.077	19.403
**Retrotransposons**	**4235099**	**71.172**	**79.779**	**23.289**
SINE	151	0.003	0.005	0*
LINE	297416	4.998	1.026	21.152
LTR	3937532	66.172	78.748	23.452
Gypsy	2815602	47.317	44.034	23.312
Copia	940479	15.805	17.394	22.666
Unknown	181451	3.049	1.490	34.035
**Unknown**	**306946**	**5.158**	**1.530**	**33.443**
	**5950488**			

Table 3. Repeat composition of the 12°C sample's unmapped sequencing reads. Enriched sequencing reads from the 12°C sample that did not map to the reference sequence (non-bisulfite treated) were aligned to the TREP repeat database using BLASTN[1] (E-value lower than 1e-5) and the number of read matches are detailed here with the % of the total transposon database hit. The same analysis performed by Brenchley *et al.*[3] on non-enriched wheat is also shown. Enriched sequencing reads from the 12°C sample that did not map to the reference sequence (bisulfite treated) were mapped to the TREP repeat database using Bismark[4] to allow calculation of the average % methylation for each transposon type i.e. the proportion of mapped reads that showed methylation across all cytosine sites. *Less than 5X coverage for analysis.

	Sample 12			Sample 27		
**Uni-Genome Methylation**	**A**	**B**	**D**	**A**	**B**	**D**
Transcribed (%)	78	78	80	78	77	80
% CpG methylation	71.5	70.6	74.1	71.5	69.0	73.0
% CHH methylation	19.6	16.8	17.9	19.9	17.6	18.4
% CHG methylation	8.9	12.6	8.0	8.6	13.4	8.6
Not Transcribed (%)	22	22	20	22	23	20
% CpG methylation	61.2	54.4	66.7	58.2	53.1	68.6
% CHH methylation	16.8	20.3	10.1	17.5	21.3	9.5
% CHG methylation	22.0	25.3	23.2	24.3	25.6	21.9
**Bi-Genome Methylation**	**BD**	**AD**	**AB**	**BD**	**AD**	**AB**
Transcribed (%)	77	77	76	77	77	77
% CpG methylation	80.7	84.3	77.5	81.2	84.4	76.4
% CHH methylation	10.0	10.7	11.4	10.9	10.4	12.5
% CHG methylation	9.3	5.0	11.1	7.9	5.2	11.1
Not Transcribed (%)	23	23	24	23	23	23
% CpG methylation	63.2	61.6	57.4	66.5	61.6	58.0
% CHH methylation	8.6	15.0	11.5	8.3	15.6	10.5
% CHG methylation	28.2	23.4	31.1	25.2	22.8	31.5
**Genome Independent Methylation**						
Transcribed (%)		73			73	
% CpG methylation		96.3			96.3	
% CHH methylation		1.2			1.2	
% CHG methylation		2.5			2.5	
Not Transcribed (%)		27			27	
% CpG methylation		59.4			59.5	
% CHH methylation		6.0			5.4	
% CHG methylation		34.6			35.1	

Table 4. Summary of orientation of the methylation sites that were analyzed in the 12°C and 27°C samples. Breakdown of methylation into uni-, bi- and tri-genome sites; transcribed (exon/intron) and non-transcribed regions and finally CpG, CHH and CHG sites.

GOID	Description	Level	P value	GOID	Description	Level	P value
**Genome A**				**Genome A**			
GO:0009605	response to external stimulus	2	0.0026	GO:0006950	response to stress	2	0.018
GO:0009991	response to extracellular stimulus	3	0.0026	GO:0033554	cellular response to stress	4	0.020
GO:0031668	cellular response to extracellular stimulus	4	0.0026	GO:0009605	response to external stimulus	2	0.0022
GO:0071496	cellular response to external stimulus	3	0.0026	GO:0009991	response to extracellular stimulus	3	0.0007
GO:0000045	autophagic vacuole assembly	8	4.09e-05	GO:0031668	cellular response to extracellular stimulus	4	0.0007
GO:0006914	autophagy	4	0.0022	GO:0071496	cellular response to external stimulus	3	0.0007
GO:0007033	vacuole organization	4	4.09e-05	GO:0000045	autophagic vacuole assembly	8	5.77e-06
GO:0009267	cellular response to starvation	6	4.09e-05	GO:0006914	autophagy	4	0.001
GO:0016236	macroautophagy	7	4.09e-05	GO:0007033	vacuole organization	4	5.77e-06
GO:0031667	response to nutrient levels	4	4.09e-05	GO:0009267	cellular response to starvation	6	5.77e-06
GO:0031669	cellular response to nutrient levels	5	4.09e-05	GO:0016236	macroautophagy	7	5.77e-06
GO:0042594	response to starvation	5	4.09e-05	GO:0031667	response to nutrient levels	4	5.77e-06
GO:0070925	organelle assembly	4	0.005	GO:0031669	cellular response to nutrient levels	5	5.77e-06
GO:0003854	3-beta-hydroxy-delta5-steroid dehydrogenase activity	6	0.012	GO:0042594	response to starvation	5	5.77e-06
GO:0016229	steroid dehydrogenase activity	3	0.015	GO:0070925	organelle assembly	4	0.0006
GO:0033764	steroid dehydrogenase activity	5	0.015	GO:0017070	U6 snRNA binding	6	0.013
GO:0006694	steroid biosynthetic process	5	0.020	GO:0003755	peptidyl-prolyl cis-trans isomerase activity	4	0.016
GO:0008202	steroid metabolic process	4	0.034	GO:0016859	cis-trans isomerase activity	3	0.016
**Genome B**				**Genome B**			
GO:0001871	pattern binding	2	1.39e-05	GO:0005575	cellular_component	0	0.009
GO:0003824	catalytic activity	1	0.0004	GO:0003824	catalytic activity	1	0.003
GO:0004028	3-chloroallyl aldehyde dehydrogenase activity	5	0.002	GO:0017057	6-phosphogluconolactonase activity	5	2.20e-18
GO:0004029	aldehyde dehydrogenase (NAD) activity	5	2.45e-05	GO:0052689	carboxylic ester hydrolase activity	4	0.007
GO:0004030	aldehyde dehydrogenase [NAD(P)+] activity	5	2.71e-05	GO:0009719	response to endogenous stimulus	2	0.0002
GO:0004812	aminoacyl-tRNA ligase activity	5	0.017	GO:0009725	response to hormone stimulus	4	0.0002
GO:0004830	tryptophan-tRNA ligase activity	6	2.73e-05	GO:0010033	response to organic substance	3	0.0002
GO:0006418	tRNA aminoacylation for protein translation	10	0.050	GO:0042221	response to chemical stimulus	2	0.0024
GO:0006436	tryptophanyl-tRNA aminoacylation	11	2.73e-05	GO:0006810	transport	3	0.0009
GO:0006633	fatty acid biosynthetic process	9	0.043	GO:0051179	localization	1	0.0006
GO:0008375	acetylglucosaminyltransferase activity	5	0.0069	GO:0051234	establishment of localization	2	0.0009
GO:0008802	betaine-aldehyde dehydrogenase activity	6	0.022	GO:0000178	exosome (RNase complex)	5	0.026
GO:0008974	phosphoribulokinase activity	5	0.050	GO:0004812	aminoacyl-tRNA ligase activity	5	0.005
GO:0009832	plant-type cell wall biogenesis	5	0.035	GO:0004830	tryptophan-tRNA ligase activity	6	0.0008
GO:0009976	tocopherol cyclase activity	3	0.050	GO:0016875	ligase activity, forming carbon-oxygen bonds	3	0.005
GO:0010215	cellulose microfibril organization	7	0.035	GO:0016876	ligase activity, forming aminoacyl-tRNA and related compounds	4	0.005
GO:0016021	integral to membrane	4	0.0003	GO:0006082	organic acid metabolic process	4	0.016
GO:0016049	cell growth	3	0.035	GO:0006418	tRNA aminoacylation for protein translation	10	0.0087
GO:0016875	ligase activity, forming carbon-oxygen bonds	3	0.017	GO:0006436	tryptophanyl-tRNA aminoacylation	11	0.0008
GO:0016876	ligase activity, forming aminoacyl-tRNA and related compounds	4	0.017	GO:0019752	carboxylic acid metabolic process	6	0.016
GO:0017057	6-phosphogluconolactonase activity	5	6.49e-12	GO:0043038	amino acid activation	8	0.010
GO:0030198	extracellular matrix organization	4	0.035	GO:0043039	tRNA aminoacylation	9	0.010
GO:0030247	polysaccharide binding	3	1.39e-05	GO:0043436	oxoacid metabolic process	5	0.016
GO:0031224	intrinsic to membrane	3	5.17e-06	GO:0044281	small molecule metabolic process	3	0.025
GO:0031225	anchored to membrane	4	0.045	GO:0016020	membrane	1	2.01e-06
GO:0031369	translation initiation factor binding	3	0.0065	GO:0016021	integral to membrane	4	1.14e-09
GO:0040007	growth	1	0.035	GO:0031224	intrinsic to membrane	3	4.98e-11
GO:0043062	extracellular structure organization	3	0.035	GO:0044425	membrane part	2	3.51e-07
GO:0044425	membrane part	2	0.0099	GO:0008375	acetylglucosaminyltransferase activity	5	7.22e-05
GO:0044765	single-organism transport	4	0.0029	GO:0044765	single-organism transport	4	2.02e-07
GO:0050269	coniferyl-aldehyde dehydrogenase activity	6	0.022	GO:0055085	transmembrane transport	5	1.51e-07
GO:0055085	transmembrane transport	5	0.004	GO:0005509	calcium ion binding	5	0.008
GO:0070726	cell wall assembly	5	0.035	GO:0022857	transmembrane transporter activity	2	0.019
GO:0071668	plant-type cell wall assembly	6	0.035	GO:0001871	pattern binding	2	0.016
GO:0072330	monocarboxylic acid biosynthetic process	8	0.0003	GO:0030247	polysaccharide binding	3	0.016
GO:0009672	auxin:hydrogen symporter activity	7	0.011	GO:0009672	auxin:hydrogen symporter activity	7	1.23e-

							05
GO:0042393	histone binding	3	0.049	GO:0022804	active transmembrane transporter activity	3	0.0009
GO:0006631	fatty acid metabolic process	8	0.043	GO:0080161	auxin transmembrane transporter activity	4	0.002
GO:0072351	tricarboxylic acid biosynthetic process	8	0.043	GO:0015399	primary active transmembrane transporter activity	4	0.025
GO:0042401	cellular biogenic amine biosynthetic process	7	0.043	GO:0015405	P-P-bond-hydrolysis-driven transmembrane transporter activity	5	0.025
GO:0032787	monocarboxylic acid metabolic process	7	0.013	GO:0003743	translation initiation factor activity	6	0.0024
GO:0030417	nicotianamine metabolic process	8	0.043	GO:0031369	translation initiation factor binding	3	0.0002
GO:0030418	nicotianamine biosynthetic process	9	0.043	GO:0080014	thalianol hydroxylase activity	4	0.044
GO:0009309	amine biosynthetic process	5	0.027	GO:0016763	transferase activity, transferring pentosyl groups	4	0.0012
GO:0030410	nicotianamine synthase activity	4	0.043	GO:0030410	nicotianamine synthase activity	4	0.0009
GO:0006082	organic acid metabolic process	4	0.013	GO:0009309	amine biosynthetic process	5	0.0025
GO:0019752	carboxylic acid metabolic process	6	0.013	GO:0030417	nicotianamine metabolic process	8	0.0009
GO:0043038	amino acid activation	8	0.013	GO:0030418	nicotianamine biosynthetic process	9	0.0009
GO:0043039	tRNA aminoacylation	9	0.013	GO:0032787	monocarboxylic acid metabolic process	7	0.0054
GO:0043436	oxoacid metabolic process	5	0.013	GO:0042401	cellular biogenic amine biosynthetic process	7	0.0054
GO:0044281	small molecule metabolic process	3	0.011	GO:0072330	monocarboxylic acid biosynthetic process	8	1.37e-05
GO:0016020	Membrane	1	0.011	GO:0072350	tricarboxylic acid metabolic process	7	0.00997
GO:0009664	plant-type cell wall organization	5	0.024	GO:0072351	tricarboxylic acid biosynthetic process	8	0.0009
GO:0022607	cellular component assembly	3	0.023	GO:0006631	fatty acid metabolic process	8	0.027
GO:0071669	plant-type cell wall organization or biogenesis	4	0.024	GO:0006633	fatty acid biosynthetic process	9	0.0050
GO:0003743	translation initiation factor activity	6	0.011	GO:0009976	tocopherol cyclase activity	3	0.0094
GO:0080014	thalianol hydroxylase activity	4	0.023				
**Genome D**				**Genome D**			
GO:0004470	malic enzyme activity	5	0.041	GO:0008661	1-deoxy-D-xylulose-5-phosphate synthase activity	4	0.043
GO:0005385	zinc ion transmembrane transporter activity	8	2.07e-06	GO:0016744	transferase activity, transferring aldehyde or ketonic groups	3	0.043
GO:0006829	zinc ion transport	9	2.07e-06	GO:0005385	zinc ion transmembrane transporter activity	8	0.015
GO:0008641	small protein activating enzyme activity	2	0.038	GO:0006829	zinc ion transport	9	0.0001
GO:0008661	1-deoxy-D-xylulose-5-phosphate synthase activity	4	0.0008	GO:0071577	zinc ion transmembrane transport	10	0.0001
GO:0009070	serine family amino acid biosynthetic process	9	0.049	GO:0008107	galactoside 2-alpha-L-fucosyltransferase activity	7	0.00496
GO:0016619	malate dehydrogenase (oxaloacetate-decarboxylating) activity	6	0.041	GO:0031127	alpha-(1,2)-fucosyltransferase activity	6	0.00496
GO:0016744	transferase activity, transferring aldehyde or ketonic groups	3	0.0008				
GO:0019725	cellular homeostasis	4	0.041				
GO:0045454	cell redox homeostasis	5	0.035				
GO:0055085	transmembrane transport	5	0.035				
GO:0071577	zinc ion transmembrane transport	10	2.07e-06				
GO:0019948	SUMO activating enzyme activity	3	0.031				
GO:0015036	disulfide oxidoreductase activity	4	0.038				
GO:0034220	ion transmembrane transport	6	0.050				
GO:0004473	malate dehydrogenase (oxaloacetate-decarboxylating) (NADP+) activity	6	0.031				
GO:0016652	oxidoreductase activity, acting on NAD(P)H, NAD(P) as acceptor	4	0.031				

Table 5. GO enrichment analysis of the uni-genome specific methylated sites in the 12°C and 27°C sample. Detailing only hits with a p value below 0.05. This analysis was carried out using GOEAST[5] default parameters and uni-genome methylated sites.

Sample 12

Contig	Start	End	P-value	q-value	Meth diff	Annotation	Allelic expression (%)
**A specific**							
CONTIG49504_435-2661-96	1	2227	2.73E-07	8.24E-07	-28.92473118	-	43.53
CONTIG877188_586-1075-0	1	490	1.29E-30	1.90E-29	-38.90009323	-	20.5
CONTIG63925_1458-2011-3	1	554	1.75E-20	1.55E-19	-25.03384615	-	21.38
CONTIG4081708_1-354-206	1	354	0.005155	0.008763	-37.23554302		17.28
CONTIG3633726_1-451-211	1	451	3.65E-167	7.97E-165	-27.79594131	-	32.78
CONTIG249042_123-339-5	1	217	6.46E-73	3.37E-71	-44.31627082	-	0
CONTIG215709_279-1230-809	1	952	3.19E-16	2.03E-15	-30.50964187	*T. aestivum* cytosolic acetyl-CoA carboxylase (Acc-2) GO:0006084	27.26
CONTIG1858325_1-731-245	1	731	9.84E-126	1.36E-123	-28.30525544	*B. distachyon* phospholipase D alpha 2-like GO:0006629	26.44
CONTIG1597600_1-795-228	1	795	2.07E-14	1.17E-13	-28.85330579	*B. distachyon* translation factor GUF1 homolog, GO:0044238	0
CONTIG1115628_3-966-261	1	964	1.73E-07	5.34E-07	-50.71428571	B. distachyon chaperone protein ClpB1-like GO:0019538	0
CONTIG108019_1166-2348-58	1	1183	0	0	-36.07309364	-	10.91
**B specific**							
CONTIG00003_659-1045-148	1	387	3.92E-12	1.82E-11	-32.31442577	-	44.62
CONTIG138549_1886-2081-70	1	196	3.67E-50	9.57E-49	-62.74428117	-	24.72
CONTIG196191_1067-1943-293	1	877	2.60E-05	6.19E-05	-26.35820896	*B. distachyon* GDSL esterase/lipase At5g45910-like GO:0008152	38.9
CONTIG2087988_1-680-139	1	680	1.06E-11	4.74E-11	-43.78571429	-	45.65
CONTIG26480_1-3308-1357	1	3308	2.57E-06	7.03E-06	-34.16149068	*B. distachyon* probable WRKY TF2 GO:0043565	39.17
CONTIG2973374_1-533-8	1	533	1.99E-59	7.05E-58	-25.49531202	-	17.94
CONTIG3001146_161-528-21	1	368	7.87E-45	1.75E-43	-46.64797862	*B. distachyon* protochlorophyllide-dependent translocon component 52, chloroplastic GO:0016020	38.87
CONTIG319969_1148-1500-157	1	353	1.93E-14	1.06E-13	-29.98905619	*T. aestivum* cytosolic acetyl-CoA carboxylase (Acc-2) GO:0006084	25.82
CONTIG3486724_1-470-37	1	470	0	0	-25.42447689	-	51.68
CONTIG373148_620-1553-214	1	934	3.32E-178	8.26E-176	-29.81705025	-	0
CONTIG428214_513-1473-246	1	961	1.65E-12	7.90E-12	-30.57077142	*A. tauschii* chr 1D prolamin gene	31.22
CONTIG673866_387-1225-49	1	839	3.05E-08	1.04E-07	-28.07017544	*B. distachyon* probable AMP deaminase GO:0046033	24.5
CONTIG757329_1-1158-925	1	1158	2.76E-65	1.07E-63	-26.7148409	-	16.48
CONTIG813109_1-1124-479	1	1124	1.53E-58	5.29E-57	-33.67509943	*B. distachyon* glucan endo-1,3-beta-glucosidase 13-like GO:0005975	35.14
CONTIG824282_1-1115-596	1	1115	7.02E-51	1.92E-49	-37.78481013	*B. distachyon* probable ADP,ATP carrier protein At5g56450 GO:0016020	17.93
**D Specific**							
CONTIG1193128_1-935-683	1	935	1.06E-254	1.17E-251	29.74587245	*H. vulgare* GDP dissociation inhibitor protein GO:0015031	62.99
CONTIG1751784_353-562-86	1	210	4.08E-197	1.64E-194	36.00680107	*B. distachyon* 14 kDa zinc-binding protein GO:0008270	52.09
CONTIG375096_1213-1553-0	1	341	5.06E-29	6.98E-28	27.98136646	-	22.71
CONTIG614988_638-844-39	1	207	5.47E-38	1.12E-36	47.59872751	-	33.59

Sample 27

Contig	Start	End	P-value	q-value	Meth diff	Annotation	Allelic expression (%)
**A specific**							
CONTIG108019_1166-2348-58	1	1183	0	0	-34.89162823	-	12.11
CONTIG1597600_1-795-228	1	795	3.57E-13	1.89E-12	-33.38197147	*B. distachyon* translation factor GUF1 homolog, GO:0044238	29.0
CONTIG1719967_1-764-545	1	764	0.004379	0.00742	-52.74193548	*B. distachyon* type I inositol 1,4,5-trisphosphate 5-phosphatase CVP2-like GO:0044238	16.8
CONTIG249042_123-339-5	1	217	3.03E-181	1.13E-178	-38.79016651	-	0
CONTIG3633726_1-451-211	1	451	1.73E-173	5.55E-171	-26.35552405	-	36.69
CONTIG4421068_1-276-58	1	276	1.56E-52	4.89E-51	-27.60867817	-	38.6
CONTIG877188_586-1075-0	1	490	1.47E-45	3.52E-44	-39.41975601	-	33.35
**B specific**							
CONTIG00003_659-1045-148	1	387	9.77E-18	6.99E-17	-37.29165498	-	42.6
CONTIG02453_4291-4580-91	1	290	9.71E-28	1.13E-26	-34.62911286	*A. tauschii* chr 1D prolamin gene	46.50
CONTIG02614_251-513-95	1	263	2.33E-11	1.11E-10	-67.19298246	T. turgidum subsp. durum ethylene response factor 1 GO:0003677	8.9

CONTIG144730_1-2152-1688	1	2152	4.45E-26	4.89E-25	-31.83300079	B. distachyon protein argonaute 16 GO:0003676	37.51
CONTIG2150797_1-584-52	1	584	0.004420	0.00809	-31.20292341	-	24.95
CONTIG26480_1-3308-1357	1	3308	2.48E-06	7.38E-06	-32.27029438	*B. distachyon* probable WRKY TF2 GO:0043565	39.15
CONTIG277654_1158-1728-159	1	571	5.57E-13	2.98E-12	-25.11293434	-	51.18
CONTIG2857336_1-546-308	1	546	2.15E-11	1.03E-10	-25.60346719	*H. vulgare* mRNA for alpha-amylase (AMY4) GO:0008152	36.80
CONTIG2973374_1-533-8	1	533	6.98E-87	4.54E-85	-25.9856896	-	25.05
CONTIG3001146_161-528-21	1	368	2.55E-66	1.03E-64	-50.67333671	*B. distachyon* protochlorophyllide-dependent translocon component 52, chloroplastic GO:0016020	35.52
CONTIG319969_1148-1500-157	1	353	7.65E-24	7.46E-23	-35.74198918	*T. aestivum* cytosolic acetyl-CoA carboxylase (Acc-2) GO:0006084	37.06
CONTIG323708_1-1634-424	1	1634	4.55E-14	2.64E-13	-32.30392157	*B. distachyon* HUA2-like protein 3	33.28
CONTIG373148_620-1553-214	1	934	2.69E-172	6.51E-170	-31.58419025	-	32.43
CONTIG428214_513-1473-246	1	961	2.89E-15	1.82E-14	-26.96503903	*A. tauschii* chr 1D prolamin gene	41.06
CONTIG4457809_1-269-105	1	269	1.64E-13	9.15E-13	-47.82608696	-	31.9
CONTIG813109_1-1124-479	1	1124	1.55E-53	4.57E-52	-30.74169514	*B. distachyon* glucan endo-1,3-beta-glucosidase 13-like GO:0005975	37.08
CONTIG824282_1-1115-596	1	1115	9.10E-54	2.71E-52	-30.07764939	*B. distachyon* ADP,ATP carrier protein At5g56450 GO:0016020	26.96
CONTIG84713_1604-1852-0	1	249	8.91E-21	7.61E-20	-27.58804963	*B. distachyon* GDSL esterase/lipase GO:0008152	24.77
**D specific**							
CONTIG00797_3966-6492-400	1	2527	2.90E-06	7.97E-06	70.58823529	-	31.47
CONTIG1193128_1-935-683	1	935	2.17E-244	1.39E-241	29.65690412	*H. vulgare* GDP dissociation inhibitor protein GO:0015031	55.66
CONTIG1751784_353-562-86	1	210	6.59E-267	9.84E-264	45.388214	*B. distachyon* 14 kDa zinc-binding protein GO:0008270	52.59
CONTIG2340265_1-633-13	1	633	7.84E-86	6.27E-84	29.48601399	-	55.56
CONTIG614988_638-844-39	1	207	7.75E-59	3.02E-57	51.82180958	-	41.41

Table 6. Differentially methylated regions identified per extended bait probe sequence in the 12°C and 27°C samples. Detailing only regions that were identified by methylkit[2] with a q value <0.01 using the Fishers exact test. The three sub-genomes of wheat were compared and regions were selected if hypermethylated by a minimum of 25% in a single genome when compared to the other two.

**Genome A average allelic expression**	**T value**	**DF (degrees of freedom)**	**P value**
Transcribed versus Promoter	5.6207	8370	<0.0001
22.33 17.12			
**Genome B average allelic expression**			
Transcribed versus Promoter	2.4488	8370	0.0144
22.25 19.87			
**Genome D average allelic expression**			
Transcribed versus Promoter	3.1696	8370	0.0015
23.46 20.39			
**Genome A average methylation**			
Transcribed versus Promoter	0.5798	8370	0.5621
7.65 7.95			
**Genome B average methylation**			
Transcribed versus Promoter	1.6314	8370	0.1028
7.85 8.73			
**Genome D average methylation**			
Transcribed versus Promoter	1.7829	8370	0.0746
7.62 8.55			

Table 7. Comparing methylation and gene expression between promoter and transcribed regions. Percentage methylation per extended bait probe sequence was summarized for each sub-genome and for transcribed and promoter regions separately (calculated as number of methylated cytosine residues at standard thresholds as a proportion of all cytosine residues in that gene region). This methylation was correlated with the percentage allelic gene expression for that sequence. For all transcribed sites, and separately promoter regions, the average percentage methylation and the average allelic gene expression was calculated. Using the two-tailed t test these average values were then compared between transcribed and promoter regions per sub-genome.

One-Sample Kolmogorov-Smirnov Test

		Non-transcribed Genome A GS	Non-transcribed Genome B GS	Non-transcribed Genome D GS	Transcribed Genome A GS	Transcribed Genome B GS
N		209	308	189	824	1295
Normal Parameters[a,b]	Mean	31.89039021119	32.76789366048	32.29609930114	32.98216318007	34.08905409478
	Std. Deviation	14.040896103150	14.755807346036	15.268315946523	11.588226295934	15.950353309142
Test Statistic		.080	.130	.098	.047	.090
Asymp. Sig. (2-tailed)		.002[c]	.000[c]	.000[c]	.000[c]	.000[c]

		Transcribed Genome D GS	Promoter Genome A GS	Promoter Genome B GS	Promoter Genome D GS
N		775	23	75	47
Normal Parameters[a,b]	Mean	34.61251229354	32.9186207652	31.34310351073	28.44383925681
	Std. Deviation	13.752502671752	12.74987733965	14.130415328627	16.470166484047
Test Statistic		.081	.226	.139	.184
Asymp. Sig. (2-tailed)		.000[c]	.004[c]	.001[c]	.000[c]

		Non-transcribed Genome A GI	Non-transcribed Genome B GI	Non-transcribed Genome D GI	Transcribed Genome A GI	Transcribed Genome B GI
N		3961	3961	3961	11765	11765
Normal Parameters[a,b]	Mean	32.1205	34.6708	33.2086	32.8588	32.6132
	Std. Deviation	13.58926	16.05794	13.22647	12.44179	13.35565
Test Statistic		.085	.096	.073	.056	.058
Asymp. Sig. (2-tailed)		.000[c]	.000[c]	.000[c]	.000[c]	.000[c]

		Transcribed Genome D GI	Promoter Genome A GI	Promoter Genome B GI	Promoter Genome D GI
N		11765	297	297	297
Normal Parameters[a,b]	Mean	34.5280	33.6948	32.5757	33.7295
	Std. Deviation	12.00605	11.02886	10.80612	12.66500
Test Statistic		.048	.164	.107	.153
Asymp. Sig. (2-tailed)		.000[c]	.000[c]	.000[c]	.000[c]

		Non-transcribed Genome A of AB BI	Non-transcribed Genome B of AB BI	Non-transcribed Genome A of AD BI	Non-transcribed Genome D of AD BI	Non-transcribed Genome B of BD BI
N		257	257	289	289	263
Normal Parameters[a,b]	Mean	26.3140	30.1308	26.0618	29.5408	30.4244
	Std. Deviation	16.45025	19.27955	16.16357	17.22703	18.02270

Test Statistic	.147	.139	.118	.088	.110
Asymp. Sig. (2-tailed)	.000[c]	.000[c]	.000[c]	.000[c]	.000[c]

	Non-transcribed Genome D of BD BI	Transcribed Genome A of AB BI	Transcribed Genome B of AB BI	Transcribed Genome A of AD BI	Transcribed Genome D of AD BI
N	263	733	733	1172	1172
Normal Parameters[a,b] Mean	31.2345	29.5347	31.5945	30.8400	33.0460
Std. Deviation	18.89392	12.94182	14.77129	14.04560	14.32645
Test Statistic	.111	.053	.078	.065	.069
Asymp. Sig. (2-tailed)	.000[c]	.000[c]	.000[c]	.000[c]	.000[c]

		Transcribed Genome B of BD BI	Transcribed Genome D of BD BI	Promoter Genome A of AB BI	Promoter Genome B of AB BI	Promoter Genome A of AD BI
N		949	949	28	28	87
Normal Parameters[a,b]	Mean	28.3654	33.0541	31.5594	37.1885	31.0379
	Std. Deviation	15.28704	16.35365	6.75352	11.64101	12.63657
Test Statistic		.065	.080	.120	.128	.163
Asymp. Sig. (2-tailed)		.000[c]	.000[c]	.200[c,d]	.200[c,d]	.000[c]

		Promoter Genome D of AD BI	Promoter Genome B of BD BI	Promoter Genome D of BD BI
N		87	28	28
Normal Parameters[a,b]	Mean	38.8841	28.2124	30.6353
	Std. Deviation	15.38425	14.19112	14.79252
Test Statistic		.224	.184	.192
Asymp. Sig. (2-tailed)		.000[c]	.016[c]	.010[c]

a. Test distribution is Normal.
b. Calculated from data.
c. Lilliefors Significance Correction.
d. This is a lower bound of the true significance.

Table 8. Testing gene expression distributions for normality. Using the One-Sample Kolmogorov-Smirnov Test to test if distributions are normal in the raw data used in the plots from figure 3 i.e. allelic gene expression for each extended bait probe for tri-methylated (GI), bi-methylated (BI) and uni-methylated residues (GS). Data is further categorized as non-transcribed, promoter and transcribed regions.

Methylation array extended bait probe	Position	Gene annotation	Further annotation	Transcription Factor binding sites	Methylation difference (>=25%)
CONTIG137059_838-2184-342<br>IWGSC_3B_10607568 (1186-2539)	336<br>CpG<br>Non-transcribed	-	-	ABRE-like sequence (early responsive to dehydration)	39.06250<br>S27 GA Increase Methylation<br>S27 GA expression increase (0.688)
CONTIG2568272_412-593-49<br>IWGSC_3B_10690222 (1248-1429)	81<br>CpG<br>exon	GO:0016021<br>GO:0055085<br>Bradi2g55130.1	probable anion transporter 3	"EEC"; Consensus motif of enhancer elements, EE-1 and EE-2 in the promoter region of the Chlamydomonas Cah1 (periplasmic carbonic anhydrase); Binding site of Myb transcription factor LCR1	28.23529<br>S27 GA Increase Methylation<br>S27 GA expression decrease (0.345)
CONTIG4841059_1-183-8<br>IWGSC_7BL_6749288 (1267-1449)	47<br>CpG<br>exon	Bradi1g31800.2<br>GO:0005975<br>GO:0042132<br>GO:0042578	Hordeum vulgare mRNA for ROP binding kinases 1 (rbk1 gene)<br>fructose 1,6-bisphosphate 1-phosphatase activity<br>phosphoric ester hydrolase activity	CAAT promoter consensus sequence	32.60870<br>S27 GA Increase Methylation<br>S27 GA expression decrease (0.40)
CONTIG92594_726-1100-179<br>IWGSC_3B_10607627 (2039-2413)	212<br>CHG<br>Non-transcribed	-	hypothetical protein VITISV_035985	"amylase box"; Conserved sequence found in 5'-upstream region of alpha-amylase gene of wheat<br>Binding site for at least two plant MYB proteins ATMYB1 and ATMYB2 (stress response)	28.50409<br>S27 GA Increase Methylation<br>S27 GA expression increase (0.683)
CONTIG102481_1825-2368-378<br>IWGSC_2AL_6436497 (9073-9610)	397<br>CpG<br>exon	Bradi5g10580.2<br>phosphoinositide 5-phosphatase activity	Oryza brachyantha ultraviolet-B receptor UVR8-like<br>Zea mays putative regulator of chromosome condensation (RCC1)	-	67.38095<br>S27 GB Increase Methylation<br>S27 GB expression no significant change (0.578)
CONTIG152583_1-2108-119<br>IWGSC_3AL_3468167 (1-678)	150<br>CHG<br>promoter	Bradi3g44310.2	nuclear hormone receptor binding, transcription coactivator activity<br>hydroxyproline-rich glycoprotein-like	Binding site of rice WRKY71, a transcriptional repressor of the gibberellin signaling pathway (defense signaling);<br>"W-box" found in promoter of Arabidopsis thaliana (response to environmental stress)	80.00000<br>S27 GB Decrease Methylation<br>S27 GB expression increase (0.602)
CONTIG2068663_2-684-0<br>IWGSC_2AS_5290061 (3656-4339)	159<br>CpG<br>Non-transcribed	Bradi1g58790.1<br>(F-box)	Triticum aestivum sucrose phosphate synthase II 3A gene<br>F-box/FBD/LRR-repeat protein At1g16930-like isoform X1	Consensus sequence of PRE (plastid response element) in the promoters of HSP70A in Chlamydomonas; Involved in induction of<br>HSP70A gene	96.29630<br>S27 GB Increase Methylation<br>S27 GB expression increase (0.651)
CONTIG2168894_1-665-369<br>IWGSC_6AS_4378555 (4847-5317)	313<br>328<br>CHG<br>Non-transcribed	Bradi3g05740.1	probable small nuclear ribonucleoprotein F	-	91.11111<br>94.11765<br>S27 GB Increase Methylation<br>S27 GB expression increase (0.608)
CONTIG2436882_1-615-227<br>IWGSC_1DL_107119 (1926-2542)	257<br>CHG<br>294	Bradi3g29420.1	Avena barbata isolate bar_19L_29 floricaula/leafy (FL) gene<br>Triticum aestivum Wknox1d gene for KN1 homeobox	"GTGA motif" found in the promoter of the tobacco (N.t.) late pollen gene g10 which shows homology to pectate lyase<br>Binding consensus sequence of Arabidopsis (A.t.) transcription<br>factor, RAV1; RAV1 specifically binds to DNA with bipartite sequence motifs of RAV1-A (CAACA) and RAV1-B (CACCTG); RAV1 protein contain AP2-like and B3-like domains	32.21477<br>28.57143<br>S27 GB Decrease Methylation<br>S27 GB expression

	CHH<br><br>Non-transcribed		protein<br><br>vacuolar protein sorting-associated protein 52 A-like isoform X3		increase (0.679)
CONTIG3442468_1-478-72<br><br>IWGSC_5DL_4577968 (3584-4062)	79<br><br>CpG<br><br>exon	GO:0008168 GO:0032259 GO:0001510<br><br>Bradi1g03600.1 rRNA (uridine-2'-O-)-methyltransferase activity	Brachypodium distachyon rRNA methyltransferase 2	-	63.63636<br><br>S27 GB Decrease Methylation<br><br>S27 GB expression no significant change (0.581)
CONTIG3940060_1-385-81<br><br>IWGSC_6BL_4386534 (4994-5382)	114<br><br>CpG<br><br>Non-transcribed	Bradi5g03070.1<br><br>WAK receptor-like protein kinase	Wall-associated receptor kinase 1	-	42.59259<br><br>S27 GB Decrease Methylation<br><br>S27 GB expression increase (0.664)
CONTIG4492671_1-260-87<br><br>IWGSC_7DL_3395927 (9706-9963)	188<br><br>CpG<br><br>exon	GO:0003677 GO:0003682	Triticum aestivum clone TaMYB64 MYB-related protein mRNA	"CGACG element" found in the GC-rich regions of the rice (O.s.) Amy3D and Amy3E amylase genes, required for expression during sugar starvation	29.46429<br><br>S27 GB Decrease Methylation<br><br>S27 GB expression no significant change (0.459)
CONTIG662333_155-1228-878<br><br>IWGSC_5BL_10899008 (1-1074)	919<br><br>CHG<br><br>Non-transcribed	-	probable metal-nicotianamine transporter YSL3	Consensus sequence of PRE (plastid response element) in the promoters of HSP70A in Chlamydomonas; enhancer within the Chlamydomonas HSP70A promoter	37.87831<br><br>S27 GB Increase Methylation<br><br>S27 GB expression no significant change (0.475)
CONTIG777882_204-523-0<br><br>IWGSC_7DL_3364770 (2562-2881)	125<br><br>CpG<br><br>exon	Bradi1g16030.1	Brachypodium distachyon transmembrane protein 230-like	"GTGA motif" found in the promoter of the tobacco (N.t.) late pollen gene g10 which shows homology to pectate lyase<br>hexamer motif of Arabidopsis thaliana (A.t.) histone H4 promoter	36.67814<br><br>S27 GB Increase Methylation<br><br>S27 GB expression no significant change (0.438)
CONTIG931153_1-1056-47<br><br>IWGSC_5AL_2746920 (5556-6612)	156<br><br>CpG<br><br>Non-transcribed	-	-	ACGT sequence (from -155 to -152) required for etiolation-induced expression of erd1 (early responsive to dehydration) in Arabidopsis<br>"CGACG element" found in the GC-rich regions of the rice (O.s.) Amy3D and Amy3E amylase genes, expression during sugar starvation	37.19149<br><br>S27 GB Increase Methylation<br><br>S27 GB expression no significant change (0.55)
CONTIG214056_1-1389-1044<br><br>IWGSC_4AL_7177172 (58-788)	1065<br><br>CHH<br><br>exon	Bradi1g00710.1	-	-	90.72039<br><br>S27 GD Decrease Methylation<br><br>S27 GD expression no significant change (0.532)
CONTIG223810_196-1857-1014<br><br>IWGSC_1AL_3870255 (1389-1635)	1066<br><br>CHG<br><br>exon	GO:0005515<br><br>Bradi2g20420.1	Setaria italica MATH domain-containing protein At5g43560-like	-	28.37838<br><br>S27 GD Increase Methylation<br><br>S27 GD expression no significant change (0.423)
CONTIG2367995_1-627-196<br><br>IWGSC_5BL_10875833 (11339-11962)	222<br><br>CpG<br><br>intron	GO:0005515<br><br>Bradi3g59200.5<br><br>microtubule-severing ATPase activity	transducin family protein-like	-	72.02737<br><br>S27 GD Decrease Methylation<br><br>S27 GD expression increase (0.623)

CONTIG2384711_304-626-85<br>IWGSC_3DL_6941321 (4426-4748)	175<br>CpG<br>exon	GO:0003677<br>GO:0003682<br>Bradi3g57300.1<br>MYB-related transcription factor	Triticum aestivum clone TaMYB48 MYB-related protein mRNA	-	80.66378<br>S27 GD Decrease Methylation<br>S27 GD expression decrease (0.331)
CONTIG3689890_1-445-186<br>IWGSC_6AL_5759538 (4304-4748)	330<br>CpG<br>exon	Bradi3g46380.1	nuclear hormone receptor binding	Motif (IVD) found in the Chlamydomonas (C.R.) Nia1 gene promoter; Located between -51 and -42; Involved in Nia1 transcription repression;	98.76543<br>S27 GD Decrease Methylation<br>S27 GD expression no significant change (0.551)
CONTIG4491143_1-259-65<br>IWGSC_4BS_4961544 (10329-10587)	152<br>CpG<br>exon	GO:0005515<br>GO:0000156<br>GO:0000160<br>GO:0006355<br>Bradi4g24970.1	Two-component response regulator-like PRR95	"GATA box"; GATA motif in CaMV 35S promoter; Binding with ASF-2;<br>Three GATA box repeats were found in the promoter of Petunia (P.h.) chlorophyll a/b binding protein, Cab22 gene; Required for high level, light regulated, and tissue specific expression; Conserved in the promoter of all LHCII type I Cab genes;	99.14530<br>S27 GD Decrease Methylation<br>S27 GD expression decrease (0.39)
CONTIG571468_1-251-70<br>IWGSC_7DL_3362089 (12358-12610)	136<br>CHG<br>170<br>CpG<br>Non-transcribed	-	-	ACGT sequence (from -155 to -152) required for etiolation-induced expression of erd1 (early responsive to dehydration) in Arabidopsis;<br>"ASF-1 binding site" in CaMV 35S promoter; ASF-1 binds to two TGACG motifs; See S000023 (AS1); Found in HBP-1 binding site of wheat histone H3 gene; TGACG motifs are found in many promoters and are involved in transcriptional activation of several genes;<br>Binding site of tobacco TGA1a; TGA1a and b show homology to CREB; TGA6 is a new member of the TGA family; Abiotic and biotic stress differentially stimulate "as-1 element" activity;<br>MYC recognition site found in the promoters of the dehydration-responsive gene rd22 and many other genes in Arabidopsis; MYC recognition sequence in CBF3 promoter; Binding site of ICE1 (inducer of CBF expression 1) that regulates the transcription of CBF/DREB1 genes in the cold in Arabidopsis;	30.34483<br>31.79567<br>S27 GD Decrease Methylation<br>S27 GD expression no significant change (0.484)
CONTIG70475_1-2644-366<br>IWGSC_4BL_7034792 (3065-5681)	519<br>CpG<br>Non-transcribed	GO:0005515<br>Bradi1g69350.1	Oryza brachyantha conserved oligomeric Golgi complex subunit 6-like	-	43.19338<br>S27 GD Decrease Methylation<br>S27 GD expression decrease (0.251)
CONTIG125368_377-2245-335<br>IWGSC_5DL_4586742 (5929-7798)	391<br>CpG<br>exon	GO:0005515<br>Bradi4g35300.2	Brachypodium distachyon C2 and GRAM domain-containing protein At1g03370-like	-	66.70455<br>S27 GBD Increase Methylation<br>S27 GBD expression decrease (0.432,0.363)
CONTIG4026253_1-366-203<br>IWGSC_3AS_1032975 (2392-2760)	227<br>CpG<br>exon	Bradi2g09180.1	Brachypodium distachyon zinc finger CCCH domain-containing protein 4	"EEC"; Consensus motif of the two enhancer elements, EE-1 and EE-2, both found in the promoter region of the Chlamydomonas Cah1 (encoding a periplasmic carbonic anhydrase); Binding site of Myb transcription factor LCR1	33.70536<br>S27 GBD Decrease Methylation<br>S27 GBD expression no significant change (0.582,0.576)
CONTIG1084855_1-982-10<br>IWGS_2DL_9902343 (3242-4223)	91<br>CpG<br>intron	-	Brachypodium distachyon cell division control protein 48 homolog B	"ASF-1 binding site" in CaMV 35S promoter; ASF-1 binds to two TGACG motifs; Found in HBP-1 binding site of wheat histone H3 gene; TGACG motifs are found in many promoters and are involved in transcriptional activation of several genes by auxin and/or salicylic acid; Binding site of tobacco TGA1a; TGA1a and b show homology to CREB; TGA6 is a new member of the TGA family; Abiotic DE and biotic stress differentially stimulate "as-1 element" activity;	43.13697<br>S27 GAD Decrease Methylation<br>S27 GAD expression increase A, decrease D (0.654,0.298)
CONTIG2057528_1-683-348<br>IWGSC_7BS_3135887 (223-909)	403<br>408<br>CpG<br>exon	Bradi1g46200.2	-	-	69.04762<br>67.07317<br>S27 GAD Increase Methylation<br>S27 GAD expression no significant change (0.599,0.455)

CONTIG3738166_1-430-225<br><br>IWGSC_2AL_1438962 (2343-2418)	120<br><br>123<br><br>189<br><br>192<br><br>CHH<br><br>Non-transcribed	Bradi5g16520.2<br><br>GTPase activity	Brachypodium distachyon RNA-binding protein 25	"GATA box"; GATA motif in CaMV 35S promoter; Binding with ASF-2; Three GATA box repeats were found in the promoter of Petunia (P.h.) chlorophyll a/b binding protein, Cab22 gene; Required for high level, light regulated, and tissue specific expression; Conserved in the promoter of all LHCII type I Cab genes;<br>"I box"; "I-box"; Conserved sequence upstream of light-regulated genes; Conserved sequence upstream of light-regulated genes of both monocots and dicots;	27.06560<br><br>31.93486<br><br>26.44538<br><br>25.22903<br><br>S27 GAD Increase Methylation<br><br>S27 GAD expression decrease A, no change D (0.391,0.593)
CONTIG3871251_1-404-195<br><br>IWGSC_6DL_3261298 (13492-13896)	266<br><br>CpG<br><br>intron	-	Oryza brachyantha outer envelope protein 64	-	53.59053<br><br>S27 GAD Decrease Methylation<br><br>S27 GAD expression increase (0.65,0.647)
CONTIG1291249_1-890-554<br><br>IWGSC_5DS_2746720 (3146-4036)	649<br><br>CpG<br><br>Non-transcribed	-	-	-	96.33508<br><br>S27 GD Decrease methylation<br><br>S27 GD expression no significant change (0.461)
CONTIG01055_4511-5766-1087<br><br>IWGSC_6AL_5772087 (1642-2897)	1111<br><br>CpG<br><br>exon	Bradi1g57700.1<br><br>lectin-like receptor kinase, putative, subfamily L-LEC	Prunus mume L-type lectin-domain containing receptor kinase IV.1-like	Core site required for binding of Dof proteins in maize (Z.m.); Dof proteins are DNA binding proteins, with presumably only one zinc finger, and are unique to plants; Four cDNAs encoding Dof proteins, Dof1, Dof2, Dof3 and PBF, have been isolated from maize; PBF is an endosperm specific Dof protein that binds to prolamin box; Maize Dof1 enhances transcription from the promoters of both cytosolic orthophosphate kinase (CyPPDK) and a non-photosynthetic PEPC gene; Maize Dof2 supressed the C4PEPC promoter;	28.91156<br><br>S27 GAB Decrease Methylation<br><br>S27 GAB expression increase A, no change B (0.687,0.569)
CONTIG143939_1-546-4<br><br>IWGSC_7AS_4242229 (1366-1911)	42<br><br>CpG<br><br>intron	Bradi1g44440.1	GTPase activity	-	93.45794<br><br>S27 GABD Increase Methylation<br><br>S27 GABD expression no change AB, increase D (0.592,0.528,0.634)
CONTIG972406_1-1026-744<br><br>IWGSC_6AS_4362220 (4607-5638)	762<br><br>CpG<br><br>Non-transcribed	-	-	-	93.33333<br><br>S27 GABD Decrease Methylation<br><br>S27 GABD expression increase (0.621,0.704,0.821)

Table 9. Differentially methylated sites between the 12°C and 27°C samples. Detailing every site where a >= 25% difference in methylation has been recorded in 1 or more sub-genomes between the 2 samples

Contig	Start	End	P-value	q-value	Meth diff s27	Expression s27	Composition	Annotation
**B specific**								
CONTIG2390219_1-624-493	1	624	9.86e-05	4.15e-03	-31.70	Decreased 0.229	Unknown/ Non-transcribed	-
CONTIG705587_1-1199-151	1	1199	9.70e-06	5.02e-04	-65.67	Increased 0.718	Transcribed	*B. distachyon* acyltransferase-like protein
CONTIG800659_1-1122-96	1	1122	4.47e-30	2.57e-027	-28.86	Increased 0.667	Unknown/Non-transcribed	*Ae. tauschii* chromosome 1Ds prolamin gene locus
**D Specific**								
CONTIG138549_1886-2081-70	1	196	1.25e-46	9.53e-44	-58.12	No change 0.49	Transcribed	B. distachyon 54S ribosomal protein L51

Table 10. Differentially methylated regions identified per extended bait probe sequence between the 12°C and 27°C samples. Detailing only regions that were identified by methylkit[2] with a q value <0.01 and a >= 25% difference in methylation using the Fishers exact test. The three sub-genomes of wheat were compared between the 12°C and 27°C samples and regions were selected if differentially methylated by a minimum of 25% in a single genome when compared to the other sample.

Extended bait probe	Position	Gene annotation	PPLR value	Gene expression phenotype (sample 27)	Methylation status (sample 27)	Transcribed/ Non-transcribed
CONTIG137059_838-2184-342	336	ABRE-like sequence (early responsive to dehydration) TF	0.688	Up-regulation	Hyper-methylated genome A	Non-transcribed
CONTIG2568272_412-593-49	81	GO:0016021 GO:0055085<br>Bradi2g55130.1 probable anion transporter 3<br>Binding site of Myb transcription factor LCR1	0.345	Down-regulation	Hyper-methylated genome A	Transcribed
CONTIG4841059_1-183-8	47	Bradi1g31800.2<br>GO:0005975 GO:0042132 GO:0042578 fructose 1,6-bisphosphate 1-phosphatase activity	0.40	Down-regulation	Hyper-methylated genome A	Transcribed
CONTIG92594_726-1100-179	212	"amylase box"; Conserved sequence found in 5'-upstream region of alpha-amylase gene of wheat<br>Binding site for at least two plant MYB proteins ATMYB1 and ATMYB2 (stress response)	0.683	Up-regulation	Hyper-methylated genome A	Non-transcribed
CONTIG152583_1-2108-119	150	Bradi3g44310.2<br>Binding site of rice WRKY71, a transcriptional repressor of the gibberellin signaling pathway (defense signaling); "W-box" found in promoter of Arabidopsis thaliana	0.602	Up-regulation	Hypo-methylated genome B	Promoter
CONTIG2068663_2-684-0	159	Bradi1g58790.1<br>(F-box)<br>F-box/FBD/LRR-repeat protein At1g16930-like isoform X1	0.651	Up-regulation	Hyper-methylated genome B	Non-transcribed
CONTIG2168894_1-665-369	313<br>328	Bradi3g05740.1<br>probable small nuclear ribonucleoprotein F	0.608	Up-regulation	Hyper-methylated genome B	Non-transcribed
CONTIG2436882_1-615-227	257<br>294	Bradi3g29420.1<br>Avena barbata isolate bar_19L_29 floricaula/leafy gene	0.679	Up-regulation	Hypo-methylated genome B	Non-transcribed
CONTIG3940060_1-385-81	114	Bradi5g03070.1<br>WAK receptor-like protein kinase	0.664	Up-regulation	Hypo-methylated genome B	Non-transcribed
CONTIG2367995_1-627-196	222	GO:0005515<br>Bradi3g59200.5<br>microtubule-severing ATPase activity<br>transducin family protein-like	0.623	Up-regulation	Hypo-methylated genome D	Transcribed
CONTIG2384711_304-626-85	175	GO:0003677 GO:0003682<br>Bradi3g57300.1<br>MYB-related transcription factor<br>Triticum aestivum clone TaMYB48 MYB-related protein mRNA	0.331	Down-regulation	Hypo-methylated genome D	Transcribed
CONTIG4491143_1-259-65	152	GO:0005515 GO:0000156<br>GO:0000160 GO:0006355<br>Bradi4g24970.1<br>Two-component response regulator-like PRR95	0.39	Down-regulation	Hypo-methylated genome D	Transcribed
CONTIG70475_1-2644-366	519	GO:0005515<br>Bradi1g69350.1<br>Oryza brachyantha conserved oligomeric Golgi complex subunit 6-like	0.251	Down-regulation	Hypo-methylated genome D	Non-transcribed
CONTIG125368_377-2245-335	391	GO:0005515<br>Bradi4g35300.2<br>Brachypodium distachyon C2 and GRAM domain-containing protein At1g03370-like	0.363	Down-regulation	Hyper-methylated genome D	Transcribed
CONTIG1084855_1-982-10	91	Brachypodium distachyon cell division control protein 48 homolog B<br>ASF-1 binding site" in CaMV 35S promoter; ASF-1 binds to two TGACG motifs; Found in HBP-1 binding site of wheat histone H3 gene; TGACG motifs are found in many promoters and are involved in transcriptional activation of several genes by auxin and/or salicylic acid; Binding site of tobacco TGA1a; TGA1a and b show homology to CREB; TGA6 is a new member of the TGA family; Abiotic and biotic stress differentially stimulate "as-1 element" activity;	0.654,0.298	Up-regulation genome A down-regulation genome D	Hypo-methylation genome AD	Transcribed
CONTIG3738166_1-430-225	120<br>123<br>189<br>192	Bradi5g16520.2<br>GTPase activity<br>Brachypodium distachyon RNA-binding protein 25	0.391	Down-regulation	Hyper-methylation genome A	Non-transcribed
CONTIG3871251_1-404-195	266	-Oryza brachyantha outer envelope protein 64	0.65,0.647	Up-regulation	Hypo-methylation genome AD	Transcribed
CONTIG01055_4511-5766-1087	1111	Bradi1g57700.1<br>lectin-like receptor kinase, putative, subfamily L-LEC<br>Prunus mume L-type lectin-domain containing receptor kinase IV.1-like	0.687	Up-regulation	Hypo-methylation genome A	Transcribed
CONTIG143939_1-546-4	42	Bradi1g44440.1<br>GTPase activity	0.634	Up-regulation	Hyper-methylation genome D	Transcribed
CONTIG972406_1-1026-744	762	-	0.621,0.704, 0.821	Up-regulation	Hypo-methylation genome ABD	Non-transcribed

Table 11. Annotation of differentially methylated sites. *Brachypodium* genes and GO annotations that have been associated with each of the contigs containing a differential methylation site (>= 25%) between the 12°C and 27°C samples plus a PPLR (Probability of Positive Log Ratio) value for the sub-genome that was differentially methylated that deviated from the baseline 0.5 by +/- 20%.

GOID	Description	Level	P value	GOID	Description	Level	P value
**Sub-Genome D Gain/Loss**				**Sub-Genome D Gain/Loss**			
GO:0016860	intramolecular oxidoreductase activity	3	0.003	GO:0000910	cytokinesis	5	0.042
GO:0006164	purine nucleotide biosynthetic process	9	0.004	GO:0009045	xylose isomerase activity	5	0.003
GO:0009152	purine ribonucleotide biosynthetic process	10	0.003	GO:0016861	intramolecular oxidoreductase activity, interconverting aldoses and ketoses	4	0.011
GO:0009165	nucleotide biosynthetic process	8	0.02	GO:0003843	1,3-beta-D-glucan synthase activity	7	0.003
GO:0009260	ribonucleotide biosynthetic process	9	0.004	GO:0000148	1,3-beta-D-glucan synthase complex	6	0.003
GO:0046390	ribose phosphate biosynthetic process	6	0.004	GO:0005886	plasma membrane	4	0.007
GO:0072522	purine-containing compound biosynthetic process	5	0.004	GO:0044459	plasma membrane part	5	0.005
GO:1901293	nucleoside phosphate biosynthetic process	7	0.027	GO:0071944	cell periphery	3	0.007
GO:0033925	mannosyl-glycoprotein endo-beta-N-acetylglucosaminidase activity	5	0.009	GO:0006074	(1->3)-beta-D-glucan metabolic process	8	0.003
GO:0006200	ATP catabolic process	11	0.0001	GO:0006075	(1->3)-beta-D-glucan biosynthetic process	10	0.003
GO:0046034	ATP metabolic process	10	0.003	GO:1901659	glycosyl compound biosynthetic process	4	0.014
GO:0006754	ATP biosynthetic process	11	0.003	GO:0008553	hydrogen-exporting ATPase activity, phosphorylative mechanism	13	0.003
GO:0009142	nucleoside triphosphate biosynthetic process	8	0.003	GO:0015662	ATPase activity, coupled to transmembrane movement of ions, phosphorylative mechanism	12	0.011
GO:0009145	purine nucleoside triphosphate biosynthetic process	9	0.003	GO:0042451	purine nucleoside biosynthetic process	8	0.003
GO:0009163	nucleoside biosynthetic process	7	0.014	GO:0042455	ribonucleoside biosynthetic process	8	0.014
GO:0009201	ribonucleoside triphosphate biosynthetic process	9	0.003	GO:0046129	purine ribonucleoside biosynthetic process	9	0.003
GO:0009206	purine ribonucleoside triphosphate biosynthetic process	10	0.003				

Table 12. GOTERM enrichment for differentially methylated sites between the sub-genome D of the 12°C sample and *Ae. tauschii*. Detailing only hits with a p value below 0.05. This analysis was carried out using GOEAST[5] default parameters.

Supplementary notes

1. Assessment of methylation conservation between sample replicates

To assess methylation conservation at positions that were assayed across the three replicates: firstly, homoeologous SNPs were used to distinguish the 3 sub-genomes (Supplementary figure 3, additional file 1). These allow categorization of reads at a position as A, B or D genome-specific or otherwise bi-allelic SNPs allow categorization of reads at a position as A or BD, B or AD and D or AB genome-specific. This enabled the percentage methylation per sub-genome or sub-genome pair to be calculated and compared between sample replicates. For all analyzed cytosine sites, the software MethylKit[2] was used to assess differential methylation between the 12°C and 27°C sample replicates, for each genome/genome-pair, by calculating Pearson correlation coefficients (Supplementary figure 4, additional file 1); samples were highly similar with all comparisons yielding coefficients >=0.97. High methylation conservation was also demonstrated between the two conditions, although when methylation changes are summarized across all cytosine sites they are likely to be comparatively small as the majority of cytosines are thought to be un-methylated. As such, Methylkit was also implemented for a pairwise comparison of sample replicates using the Fisher exact test; across all positions the average standard deviation between replicates in the 12°C and 27°C samples was 1.96 and 1.85 and <0.09% residues were identified as differentially methylated between the sample replicates ($P<0.01$). These values indicate high conservation of methylation between replicate samples; moreover, any sites that were considered differentially methylated between replicates were excluded from sample-wise comparisons.

2. Investigating the uniformity of enrichment

To assess if uniform enrichment was seen from all three of the wheat sub-genomes the 28,632 and 29,202 cytosine sites for the 12°C and 27°C samples respectively, where all three sub-genomes could each be identified at a depth of 5X or more, were analyzed and the percentage of reads per sub-genome were calculated; 33.3% for sub-genome A, 33.3% for sub-genome B and 33.4% for sub-genome D. Therefore, enrichment was largely unbiased between the three sub-genomes. To investigate if methylation state affected enrichment; at sites where only one of the A, B and D sub-genomes showed methylation the average depth of coverage of the methylated sub-genome across the dataset was calculated and compared to the average coverage of the other two non-methylated sub-genomes at each position. This analysis was conducted across the 2 samples. The mean and standard deviation of coverage per sub-genome in the non-methylated group (mean=56.6, SD=71.0) were compared to those of the methylated group (mean=70.7, SD=89.5). There was a large overlap in the datasets and no significant difference was found between them ($p=0.8775$, two-tailed t test). Therefore, we were able to conclude there was no bias towards enrichment of methylated or non-methylated DNA sequence.

Efficient enrichment of the three sub-genomes was seen in non-transcribed regions when homoeologous SNPs were available for genome discrimination. When it was possible to discriminate the three sub-genomes at a minimum depth of 5X, there was no apparent difference between the capture efficiency of transcribed and non-transcribed regions: In transcribed exon/intron regions, in the 12°C sample, the percentage of reads mapping to each of the A, B and D sub-genomes was 33.2%, 33.3% and 33.5% respectively; In un-transcribed regions the percentages were 34.0%, 33.3% and 32.7% respectively; both tend towards the average across all regions and thus to ~33% of reads mapping to each sub-genome A, B and D.

Of the 7,813,105 and 8,069,906 cytosines, that were identified in the 12°C and 27°C samples respectively (Supplementary figure 6, stage 2); 49% are in regions that are predicted to be non-transcribed i.e. not hitting intron/exon sequence. Such proportions are predictable considering that ~45% of the full 44Mb mapping reference is predicted to be non-transcribed. Conversely, when we identify homoeologous SNPs within the mapping reference 22% are predicted to be un-transcribed and following on from this similar proportions of un-transcribed residues are seen for the 318,452 and 324,227 analyzed cytosine residues for the 12°C and 27°C samples with ~20% un-transcribed i.e. residues that could be associated with these homoeologous SNPs.

The ~20% of the 318,452 and 324,227 analyzed residues for the 12°C and 27°C samples that were in non-transcribed regions (supplementary figure 5, Stage 4) is in line with the percentage of available non-transcribed homoeologous SNPs for sub-genome discrimination (~22%). However this proportion deviates significantly from the initial 44 Mb mapping reference where ~45% is un-transcribed; analysis of those sites that could be associated with homoeologous SNPs results in a skew towards analysis of sites in transcribed sequence as proportionally fewer homoeologous SNPs could be confidently identified in the non-transcribed regions. This most likely reflects the targeting of our 120-Mer probe sequences to transcribed sequence and as such we see the highest depth of coverage in these transcribed probe regions. Since the enriched sequencing reads were used to define homoeologous SNPs by mapping to the IWGSC reference sequence this may explain the bias towards homoeologous SNPs in transcribed sequence. While this effect is un-desirable, stifling the identification of homoeologous SNPs in non-transcribed regions, here its effect has been minimal. As such, ~1/5 of the final dataset of cytosine residues for analysis (Figure 6, Stage 4) are found in un-transcribed regions and have been successfully associated with homoeologous SNPs.

3. Transposon methylation state assessment through the analysis of off target sequence

Over the 2 samples on average 31,939,028 (~20%) sequencing reads in each case were mapped to the reference sequence. It is estimated that ~63% of off target sequencing reads matched repetitive sequence. Sequencing data for the 12°C sample, that could not be mapped to the reference sequence and had been enriched but not bisulfite treated, was aligned to the wheat TREP database using BLASTN[1] (E-value less than 1e-5) to see if repetitive sequence could be

identified/categorized. This off-target sequence should be unbiased carryover DNA and equivalent to shotgun sequencing total wheat DNA at low coverage. To test this we compared the proportions of transposon types in the enriched data and found that they closely matched the proportions seen in previous shotgun sequence data[3] (see supplementary table 3). It is also possible that the transposon sample may by biased to transposons closely associated with genic portions of the genome, to test this we investigated the proportion of paired end reads mapped to the extended bait reference sequence at one end of the pair and transposon sequence at the other; we identified only 1.35% of mapped reads with a paired end of this type.

The unmapped enriched bisulfite treated sequencing reads were also mapped to the Triticeae Repeat Sequence database (TREP) sequences using Bismark[4] to allow the study of transposon/repetitive DNA methylation. For each transposon type, mapped reads were analyzed across all of the cytosine residues, the percentage of mapped reads overall showing methylation was calculated. It was noted that transposons in general were hyper-methylated, 24% of reads showed methylation across all cytosine sites, in comparison to the same analysis of the mapped reads across the enriched gene rich regions where we see such methylation in ~12.3% of the reads. The retro transposon group SINE was the only exception to this where no methylation was seen, however this group gained less than 5X coverage and as such this may not be a reliable result.

4. Conserved proportions of uni-genome and bi-genome methylation across the respective genomes/genome-pairs

In the 12°C sample; 30.0% of uni-genome methylation was from sub-genome A, 37.1% from sub-genome B and 32.9% from sub-genome D. In the 27°C sample; 31.9% of the uni-genome methylation was from sub-genome A, 35.4% from sub-genome B and 32.7% from sub-genome D. In both cases the 3 percentages were found not to be significantly different to the expected percentage of 1/3 (Sample 12; $X^2 = 3.847$, $p = 0.1461$ and Sample 27; $X^2 = 0.988$, p 0.6103). For these calculations residues were only used if all three sub-genomes could be identified independently each at a depth of 5X; this avoids bias for uni-genome methylation that could be introduced by using uneven proportions of biallelic homoeologous SNPs i.e. distinguishing genome A:BD, B:AD and D:AB. In the 12°C sample; 34.5% of bi-genome methylation was from sub-genomes B and D, 32.9% from sub-genomes A and D and 32.6% from sub-genomes A and B. In the 27°C sample; 35.5% of bi-genome methylation was from sub-genomes B and D, 31.8% from sub-genomes A and D and 32.7% from sub-genomes A and B. In both cases the 3 percentages were found not to be significantly different to the expected percentage of 1/3 (Sample 12; $X^2 = 0.892$, $p = 0.6403$ and Sample 27; $X^2 = 0.275$, p 0.8714).

References

1. Altschul, S. F., Gish, W., Miller, W., Myers, E. W. and Lipman, D. J. Basic local alignment search tool. *J. Mol. Biol.*, **215,** 403-410 (1990)

2. Akalin, A. *et al*. methylKit: a comprehensive R package for the analysis of genome-wide DNA methylation profiles. *Genome Biol* **13,** R87 (2012).

3. Brenchley, R. *et al.* Analysis of the bread wheat genome using whole-genome shotgun sequencing. *Nature,* **491,** 705-710 (2012)

4. Krueger, F. and Andrews, S. R. Bismark: a flexible aligner and methylation caller for Bisulfite-Seq applications. *Bioinformatics,* **27,** 11:1571-1572 (2011)

5. Zheng, Q. & Wang, X. J. GOEAST: a web-based software toolkit for Gene Ontology enrichment analysis. *Nucleic Acids Res* **36,** W358–W363 (2008).
